# The sirtuin family in health and disease

**DOI:** 10.1038/s41392-022-01257-8

**Published:** 2022-12-29

**Authors:** Qi-Jun Wu, Tie-Ning Zhang, Huan-Huan Chen, Xue-Fei Yu, Jia-Le Lv, Yu-Yang Liu, Ya-Shu Liu, Gang Zheng, Jun-Qi Zhao, Yi-Fan Wei, Jing-Yi Guo, Fang-Hua Liu, Qing Chang, Yi-Xiao Zhang, Cai-Gang Liu, Yu-Hong Zhao

**Affiliations:** 1 Liaoning Key Laboratory of Precision Medical Research on Major Chronic Disease, Shengjing Hospital of China Medical University, Shenyang, China; 2 Department of Clinical Epidemiology, Shengjing Hospital of China Medical University, Shenyang, China; 3 Department of Obstetrics and Gynecology, Shengjing Hospital of China Medical University, Shenyang, China; 4 Clinical Research Center, Shengjing Hospital of China Medical University, Shenyang, China; 5 Department of Pediatrics, Shengjing Hospital of China Medical University, Shenyang, China; 6 Department of Oncology, Shengjing Hospital of China Medical University, Shenyang, China; 7 Department of Urology, Shengjing Hospital of China Medical University, Shenyang, China; 8 Department of Cancer, Breast Cancer Center, Shengjing Hospital of China Medical University, Shenyang, China

**Keywords:** Molecular biology, Diseases, Gene expression analysis

## Abstract

Sirtuins (SIRTs) are nicotine adenine dinucleotide(+)-dependent histone deacetylases regulating critical signaling pathways in prokaryotes and eukaryotes, and are involved in numerous biological processes. Currently, seven mammalian homologs of yeast Sir2 named SIRT1 to SIRT7 have been identified. Increasing evidence has suggested the vital roles of seven members of the SIRT family in health and disease conditions. Notably, this protein family plays a variety of important roles in cellular biology such as inflammation, metabolism, oxidative stress, and apoptosis, etc., thus, it is considered a potential therapeutic target for different kinds of pathologies including cancer, cardiovascular disease, respiratory disease, and other conditions. Moreover, identification of SIRT modulators and exploring the functions of these different modulators have prompted increased efforts to discover new small molecules, which can modify SIRT activity. Furthermore, several randomized controlled trials have indicated that different interventions might affect the expression of SIRT protein in human samples, and supplementation of SIRT modulators might have diverse impact on physiological function in different participants. In this review, we introduce the history and structure of the SIRT protein family, discuss the molecular mechanisms and biological functions of seven members of the SIRT protein family, elaborate on the regulatory roles of SIRTs in human disease, summarize SIRT inhibitors and activators, and review related clinical studies.

## Introduction

The sirtuin (SIRT) protein family, which are conserved proteins belonging to class III histone deacetylases, comprises seven members.^1^ Notably, SIRTs share a nicotine adenine dinucleotide + (NAD) + -binding catalytic domain and may act specifically on different substrates depending on the biological processes in which they are involved.^2^ The sequence and length of SIRTs are different in both their N- and C-terminal domains, partially explaining their different localization and functions.^2^ Recently, more and more studies have shown their association with and involvement in different pathologies, such as (but not restricted to) cancer and cardiovascular diseases (CVDs).^3–6^ Additionally, increasing evidence supported the potential use of SIRT modulators for the treatment of different kinds of diseases,^7–11^ suggesting the critical roles of SIRTs in the diseases. Herein, to enhance our understanding of SIRTs, we provide a comprehensive summary of the roles of SIRTs in health and various diseases.

### Historical review and structure of SIRT proteins

The history of SIRTs can be traced to founding member Sir2 nearly 40 years ago, which was first discovered in the budding *Saccharomyces cerevisiae*, and was originally known as mating-type regulator 1 protein.^12^ Subsequently, Sir2 has been found to function in transcriptional repression at ribosomal DNA loci,^13^ at silent mating-type loci^14^ and in telomeres,^15^ and this increasing knowledge has greatly improved exploration of its function. In the late 1990s, a study confirmed that Sir2 prolonged the lifespan of yeast by inhibiting genomic instability. Loss of Sir2 significantly shortened the lifespan of yeast, while an additional copy of Sir2 prolonged it by about 40%.^16^ Later evidence showed that Sir2 had NAD + -dependent HDAC enzymatic activity, which provided a molecular framework in which NAD-dependent histone deacetylation could be connected to genomic silencing and ageing in yeast, and possibly to higher eukaryotic metabolism as well, opening a new chapter of Sir2 enzymology.^17^ Sir2’s key role in the molecular mechanism of senescence in *Caenorhabditis elegans* was also later demonstrated.^18^ As Sir2 homologous genes have been successively isolated in bacteria, plants and mammals, the Sir2 homologous proteins in all species have been collectively referred to as SIRTs.^19,20^

**Fig. 1:**
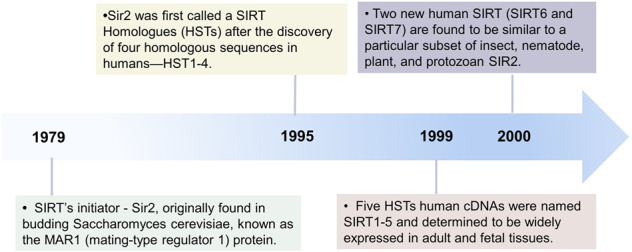
The historical timeline on milestones in SIRT family members

Currently, seven mammalian homologs of yeast Sir2 named SIRT1 to SIRT7 have been identified, which are well-known as the β-NAD + or NAD + -dependent enzymes.^21–23^ Figure 1 shows a historical timeline summarizing studies on milestones in SIRT family members. Regarding to the molecular structures, SIRT1-7 share a chemically and structurally conserved catalytic core in general and there may be subtle differences in the infrastructure of active site.^24^ In detail, X-ray crystalline diffraction reveals that the catalytic core includes two bilobed globular domains consisting of approximately 275 amino acids residues, characterized by their necessity for NAD as a cofactor. The different N- and C-terminals of SIRT proteins are fairly variable in length, chemical composition, susceptibility to post-translational modifications (PTMs) (typically phosphorylation), and enable them to bind substrates.^2,25,26^ The large structural domain is composed of an inverted classical open α/β Rossmann-fold structure, which is a parallel β-sheet nucleotide-binding fold typical of many NAD-utilizing enzymes such as dehydrogenases; in addition, a smaller domain contains a zinc ribbon motif. These two domains form a pocket in the middle where NAD and acetylated peptides bind.^2,27^

Differences among members of the SIRT protein family were initially attributed to their discrete pattern of subcellular localization.^28^ As far as we know, SIRT1 is mainly localized in the nucleus and shuttles to the cytosol under specific circumstances.^29,30^ SIRT2 is predominantly cytosolic but also exists in the nucleus in the G2 to M phase transition of the cell cycle.^31^ SIRT3-5 localize primarily to mitochondria, and have a mitochondrial targeting sequence.^32–34^ Additionally, SIRT6 and SIRT7 are nuclear proteins. Of them, SIRT6 is principally located in the chromatin and SIRT7 is mostly found in the nucleolus.^35,36^ Additionally, the localization and subcellular shuttling of SIRTs depend on different kinds of cell types and cell cycle oscillation.^37^ For example, SIRT1 could be primarily located in the cytosol in some subsets of neurons, as well as expressed in both nucleus and cytosol in ependymal cells.^30^ Moreover, SIRT2 is in the cytosol during most phases of cell cycle, while SIRT2 is expressed in nucleus and associates with chromatin and deacetylates the histone H4K16 during G2/M transition and mitosis.^31^

The catalytic activity level of SIRT protein family members is thought to be their second most significant difference. Of note, the regulation of catalytic activity of SIRTs involves multiple steps: (a) NAD + and acetyl lysine substrates binding; (b) the glycosidic bond cleavage; (c) acetyl transfer; and (d) O-acetyl-ADPR, nicotinamide, and deacetylated lysine products formation. Concretely, the initial reaction of NAD + glycosidic bond cleavage is proceeded through either an SN1-like mechanism, as supported by the structure of Hst2 bound to carba-NAD + ,^38^ or an SN2-like mechanism, as supported by the structure of Sir2Tm bound to NAD^+^ and an acetyl lysine-containing peptide.^39^ Furthermore, available studies suggested a complex array of PTMs regulated by SIRTs. Initially, Sir2 was considered solely as a deacetylase enzyme.^17^ However, the functional range of enzymatic activities of SIRTs has been greatly expanded in mammals. SIRT1-3 sustain strong deacetylase activities. SIRT4 has ADP-ribose transferase activity and can down-regulate glutamate dehydrogenase activity in β cells, thereby reducing insulin secretion response.^33^ SIRT5 is involved in regulating protein post translational modifications such as lysine succinylation, malonylation, and glutarylation, etc.^40,41^ Moreover, SIRT6 can function as NAD + -dependent monoADP-ribosyl transferase and long-chain fatty acyl deacetylases.^42,43^ Meanwhile, SIRT7, the latest discovered SIRT family protein, has been relatively less studied, which was first found to be a β-NAD + -dependent deacetylase enzyme and is localized in nucleoli that govern the transcription of RNA polymerase I.^44,45^ Numerous target proteins, including histone and non-histone, have been shown to be modified by SIRTs, and participates in the regulation of multiple fundamental cellular functions including glucose, and lipid metabolism, mitochondrial biogenesis, DNA repair, oxidative stress, apoptosis, and inflammation.^46^ Hence, SIRTs are now recognized as a major regulator of cellular physiology. Nevertheless, the SIRT protein family still has multiple proven and unproven catalytic modification activities. Given our current limited understanding of the SIRT protein family, more investigation is warranted in this area.

### The regulatory role of SIRTs in cellular biology

#### The role of SIRTs in inflammation

**Fig. 2:**
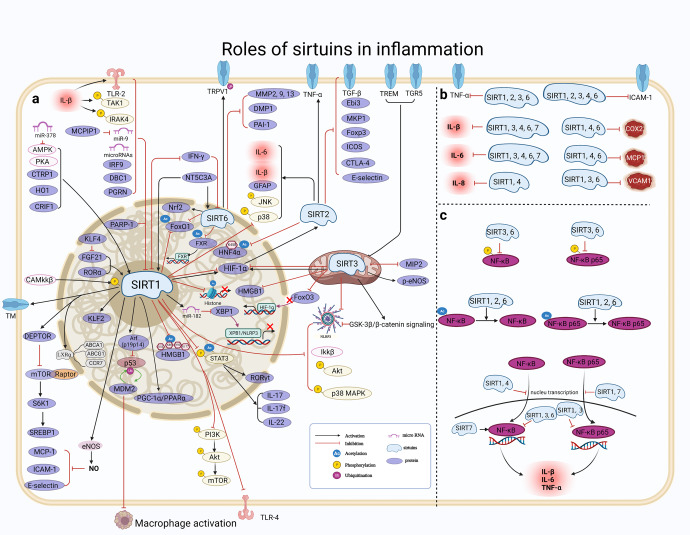
Overview of the roles of SIRTs in inflammation. **a** SIRTs mainly play an anti-inflammatory effect by regulating inflammatory mediators, however, early inhibition of SIRT2 may prevent neuroinflammation evidenced by reduced levels of GFAP, IL-β, IL-6, and TNF-α; (**b**) SIRTs could negatively regulate several pro-inflammatory cytokines; (**c**) SIRTs are involved in the regulation of NF-κB signaling pathway. https://biorender.com. ABCA1 ATP‑binding cassette A1, ABCG1 ATP‑binding cassette G1, Arf alternative reading frame, CaMKKβ Ca(2 + )/calmodulin-dependent protein kinase kinase β, CCR7 C‑C chemokine receptor type 7, CRIF1 CR6-interacting factor1, CTLA4 cytotoxic T lymphocyte–associated antigen 4, CTRP1 C1q/tumor necrosis factor-related protein 1, DBC1 deleted in breast cancer 1, DEPTOR DEP-domain containing mTOR-interacting protein, DMP1 dentin matrix protein-1, Ebi3 Epstein-Barr virus–induced gene 3, FGF21 fibroblast growth factor 21, FXR farnesoid X receptor, GFAP glial fibrillary acidic protein, HIF-α hypoxia-inducible factor-alpha, HMGB1 high-mobility group box 1, HNF4α hepatocyte nuclear factor 4α, HO1 heme oxygenase-1, ICOS inducible T cell co-stimulator, IFN-γ interferon-γ, IKKβ inhibitor kappa B kinaseβ, IRAK interleukin-1 receptor-associated kinase, IRF9 interferon regulatory factor 9, LXR liver X receptor, MCP monocyte chemotactic protein, MCPIP1 MCP-1 induced protein, MIP-2 macrophage inflammatory protein-2, MKP-1 mitogen-activated protein kinase phosphatase-1, NT5C3A pyrimidine 5'-nucleotidase, PAI-1 plasminogen activator inhibitor-1, PARP-1 peroxisome proliferator-activated receptor 1, PGRN progranulin, RORγt RAR-related orphan receptor γ-t, TAK1 transforming growth factor β activated kinase-1, TM thrombomodulin, VCAM-1 vascular cell adhesion molecule-1, XBP1 X-box binding protein 1

Inflammation is an essential immune response that enables survival during infection or injury and maintains tissue homeostasis under a variety of noxious conditions.^47^ It comes at the cost of a transient decline in tissue function, which can in turn contribute to the pathogenesis of diseases involving altered homeostasis and a variety of physiological and pathological processes.^48^ The molecular process of inflammation is varied and depends on the type of inflamed cells and organs. The inflammatory response is composed of several inseparable pathways involving inflammatory cells, inflammatory mediators induced by sensor cells, inflammatory pathway components, and the target tissues that are affected by the inflammatory mediators.^47^ Recently, with greater in-depth understanding of the process of inflammation, numerous studies have successfully illustrated how the SIRT protein family has a close association with inflammation. In this section, we summarize the role of the SIRT family in the inflammatory response and the major signaling pathways (Fig. 2).

##### The effect of SIRTs in inflammatory cells

The cells involved in the inflammatory response include inflammatory cells such as macrophages, mast cells and endothelial cells. SIRTs, especially SIRT1 and SIRT6, can affect the secretion of inflammatory mediators and play a central role in regulating the differentiation of dendritic cells (DCs) and the activation of macrophages.^49,50^ For example, SIRT1 participates in mediating inflammatory signaling in DCs, consequentially modulating the balance of proinflammatory T helper type 1 cells and anti-inflammatory Foxp3(+) regulatory T cells. SIRT1 knockout (KO) in DCs restrained the generation of regulatory T cells while driving T helper 1 cell development, resulting in enhanced T-cell-mediated inflammation against microbial responses.^49^ Moreover, SIRT6 deficiency in macrophages resulted in inflammation with increases in acetylation and greater stability of the forkhead box protein O1 (FoxO1). Conversely, the ectopic overexpression of SIRT6 in KO cells reduced the inflammatory response.^50^ Moreover, results from in vivo experiments demonstrated that SIRT3 overexpression in transfused macrophages not only induced M2 macrophage polarization, but also alleviated inflammation.^51^ Based on these current studies, the SIRT family may regulate the activation or differentiation of inflammatory cells, such as DCs and macrophages in the immune system.

##### The effect of SIRTs on inflammatory mediators

Inflammatory mediators are chemicals produced during inflammation that cause an inflammatory response. In response to the inflammatory process, inflammatory cells release specialized substances, including vasoactive amines and peptides, eicosanoids, proinflammatory cytokines and acute-phase proteins, which mediate the inflammatory process by preventing further tissue damage and ultimately resulting in healing and restoration of tissue function.^52^ Overexpressed or activated SIRTs, mainly SIRT1–3, can reduce the inflammatory response through anti-inflammatory effects, such as tumor necrosis factor-alpha (TNF-α), a multifunctional pro-inflammatory cytokine, which is produced by macrophages/monocytes during acute inflammation, and plays a critical role with orchestrating the cytokine cascade in various inflammatory diseases.^53^ For instance, increased SIRT1 protein expression can reduce acetylation of the nuclear factor kappa-B (NF-κB) p65 subunit, which results in the suppression of TNF-α-induced NF-κB transcriptional activation and reduction of TNF-α secretion in a SIRT1-dependent manner.^54,55^ In addition, SIRT1 knockdown increased, while SIRT1 activator treatment decreased TNF-α secretion from macrophages.^55^ One recent study verified that SIRT6 suppressed inflammatory responses and downregulated the expression of inflammatory factors interleukin (IL)-6 and TNF-α via the NF-κB pathway.^56^ For example, both SIRT1 and SIRT6 inhibited TNF-α-induced inflammation of vascular adventitial fibroblasts through reactive oxygen species (ROS) and the protein kinase B (Akt) signaling pathway.^57^ SIRT1 exerted anti-inflammatory effects against IL-1β-mediated pro-inflammatory stress through the Toll-like receptor 2 (TLR2)/SIRT1/NF-κB pathway.^58^ SIRT1 deficiency increased microvascular inflammation in obese septic mice, while resveratrol treatment decreased leukocyte/platelet adhesion and E-selectin/intercellular adhesion molecule (ICAM-1) expression accompanied by increased SIRT1 expression and improved survival.^59^ In addition, SIRT1 and SIRT6 inhibited inflammation by decreasing pro-inflammatory cytokines such as IL-6, IL-β, cytochrome oxidase subunit 2 and ICAM-1.^60^ Moreover, SIRT1 exerted anti-inflammatory effects against IL-1β-mediated pro-inflammatory stress through the TLR2/SIRT1/NF-κB pathway.^58^ SIRT1 deficiency increased microvascular inflammation in obese septic mice, while resveratrol treatment decreased leukocyte/platelet adhesion and E-selectin/ICAM-1 expression accompanied by increased SIRT1 expression and improved survival.^59^ Recently, SIRT2 as modulators have been shown to be effective in inhibiting lipopolysaccharide-stimulated production of TNF-α to suppress neuroinflammation.^61,62^ Moreover, Kurundkar et al. have determined that SIRT3 deficiency altered the proinflammatory responses of macrophages to lipopolysaccharides, with a greater increase in TNF-α production.^63^ Several studies have also shown an anti-inflammatory effect of SIRT3, which downregulates IL-1β and IL-18, inhibits inflammasomes and attenuates oxidative stress.^64,65^ SIRT3 KO mice have significantly increased inflammatory cell infiltration.^66^ These studies highlight the critical role of SIRT3 in the process of inflammation. In conclusion, then, as one of the most important pro-inflammatory cytokines, inflammatory mediators are closely regulated by the SIRT protein family and is widely involved in inflammation.

Currently, the SIRT family mainly exerts an anti-inflammatory effect in response to tissue stress or disease development, but there are exceptions. For example, early SIRT2 inhibition prevented neuroinflammation evidenced by reduced levels of glial fibrillary acidic protein, IL-1β, IL-6 and TNF-α and by increased levels of glutamate receptor subunits GluN2A, GluN2B and GluA1; however, SIRT2 inhibition was unable to reverse cognitive decline or neuroinflammation.^67^ In this case, SIRT2 exhibited a temporary proinflammatory effect. Furthermore, both pro- and anti-inflammatory effects have been attributed to SIRT2 and SIRT3.^68^ Single deficiency of SIRT2 or SIRT3 had minor or no impact on the antimicrobial innate immune responses, while SIRT2/3^−/−^ macrophages secreted increased levels of both proinflammatory and anti-inflammatory cytokines.^68^ From these results, then, most SIRT proteins appear to play anti-inflammatory roles, but limited reports have found the opposite effect, as just described for SIRT2. These inconsistent results might be due to the specificity of SIRT2 mechanisms in the SIRT family, or may be temporary effects manifested at different stages of the disease process. Therefore, more research is needed to explore the reasons for these discrepancies.

Overall, SIRTs can act in concert or compensate each other for certain immune functions.^68^ It is also worth noting that the effects of various SIRTs may differ between diseases, or even have opposite effects. Therefore, research on SIRTs has left a number of gaps which require further exploration to pinpoint the role of the SIRT family in inflammatory responses and the underlying mechanisms of action, which may account for the different results.

##### The effect of SIRTs on inflammatory pathway components

The signaling pathway of inflammation is complex, but inflammatory pathway components have begun to be elucidated over the past several years. Currently, there are many studies on the mechanisms by which the SIRT family participates in inflammation, especially pathways involving NF-κB, TNF-α, and the NOD-, LRR- and pyrin domain-containing protein 3 (NLRP3) inflammasome.

NF-κB is considered to be the central regulator of inflammation, which drives the expression of cytokines, chemokines, inflammasome components and adhesion molecules.^69^ It is mainly involved in immune and inflammatory responses and can induce the expression of downstream inflammatory cytokines.^70,71^ TNF-α is a pro-inflammatory cytokine mainly produced by macrophages and monocytes and is involved in normal inflammatory and immune responses.^72^ As an important component of innate immunity, the NLRP3 inflammasome plays an important role in the body’s immune response and inflammatory cell death (pyroptosis).^73^ In the following sections, we detail the role of the SIRT family as it affects three key inflammatory pathway components.

1. **Majority of SIRTs exert anti-inflammatory effects by inhibiting the NF-κB pathway**
  NF-κB exists in multiple forms, with the heterodimer of p65 (RelA, Rel associated protein) and p50 subunits (p65/p50) being the most prevalent species.^74^ In the absence of stimulation, NF-κB is normally present in the cytoplasm in an inactive form. Upon stimulation by various pro-inflammatory cytokines (such as IL-1β, IL-6 and TNF-α), NF-κB rapidly translocates to the nucleus and regulates the transcription or expression of target genes.^75,76^ In addition, NF-κB activity can be modulated by PTMs of proteins, such as acetylation.^77^ Most members of the SIRT family are involved in regulation of the NF-κB pathway, primarily including SIRT1, SIRT2, SIRT6, and SIRT7.
  Growing evidence suggests the significant role of SIRTs in the regulation of inflammation. SIRT1 has anti-inflammatory effects mediated by the deacetylation and inactivation of the p65 subunit of NF-κB.^78^ SIRT1 inhibits the transcriptional activity of NF-κB via deacetylation of the p65 (RelA) subunit at Ac-Lys310.^78^ Furthermore, the finding that lower SIRT1 activity levels may increase the expression of NF-κB, thus driving inflammation,^79^ also highlight the important role of SIRT1 during inflammation.
  Repression of NF-κB activity is responsible for the anti-inflammatory effect of SIRT6.^80^ For instance, SIRT6 attenuated NF-κB expression by deacetylating histone H3K9 in the promoters of NF-κB target genes, hence decreasing inflammation.^80^ Additionally, SIRT6 overexpression suppressed NF-κB-mediated inflammatory responses in OA development.^81^ Since nuclear SIRT1 and SIRT6 deacetylate RelA/p65 and support its degradation by the proteasome, decreases in both SIRT1 or SIRT6 levels/activity increase NF-κB activity and amplify pro-inflammatory gene expression during chronic inflammation.^82^
  Evidence concerning the role of SIRT7 in inflammatory processes has been somewhat inconsistent. In terms of mediating an anti-inflammatory response, knockdown of SIRT7 promoted the translocation of NF-κB p-p65 to the nucleus and subsequently increased the secretion of downstream inflammatory cytokines, while SIRT7 overexpression had the opposite effect.^83,84^ However, evidence also suggested that loss of SIRT7 promoted the translocation of NF-κB p65 to the cytoplasm.^85^ Thus, the roles of SIRT7 in p65 translocation is controversial. In addition, the decline of SIRT7 upregulated the levels of pro-inflammatory cytokines including IL-1β and IL-6 in human umbilical vein endothelial cells, while overexpression of SIRT7 effectively alleviated the inflammatory response.^86^ However, several studies have also revealed a pro-inflammatory role for SIRT7. For example, SIRT7-kidney-specific KO mice exhibited diminished inflammation with a reduction in the level of multiple inflammatory factors such as TNF-α, IL-1β and IL-6, and suppression of nuclear NF-κB p65 accumulation.^87^ These contradictory results imply that the regulatory effects of SIRT7 on the inflammatory process may be variable under specific pathologies, which will need further study.^84^
  SIRT2 also participates in inflammatory responses. Inhibition of SIRT2 enhanced microglial activation and the release of pro-inflammatory cytokines via acetylation-dependent upregulation of NF-κB transcriptional activity.^88^ SIRT2 reduced the levels of pro-inflammatory cytokines and ameliorated the severity of arthritis by deacetylating the p65 subunit of NF-κB,^89^ further demonstrating the role of SIRT2 activation in suppression of the inflammatory response.
  In summary, SIRTs are found to interfere with the NF-κB signaling pathway by preventing NF-κB translocation, influencing its expression and regulating its interactions, thereby having an anti-inflammatory function. Understanding the underlying molecular mechanisms of NF-κB pathway activation and its effects on inflammation may guide an approach to designing better pharmacological targets for alleviating inflammation and related therapies.
2. **The activation of NLRP3 aggravates inflammation**
  NLRP3 is an important component of the NLRP3 inflammasome complex involved in inflammation.^90,91^ It is believed that activation of the NLRP3 inflammasome occurs in two sequential steps — first, it must be primed, and then it can be activated.^71^ When the body suffers from inflammatory disease, damage-associated molecules directly engage TLR4 and then quickly activate the NF-κB signaling pathway, resulting in augmented expression of NLRP3;^92–94^ this in turn generates inflammatory cytokines such as IL-1β, IL-18, TNF-α and transforming growth factor-beta (TGF-β) which aggravate inflammation.^95^ Some studies have found that SIRTs, especially SIRT1 and SIRT3, act on NLRP3 to exert anti-inflammatory functions. For example, SIRT1 plays an important protective role in the inflammation mediated by the attenuation of NLRP3 activity, which is the best characterized inflammasome.^96,97^ Mechanistic studies of acute liver injury^98^ demonstrated activation of a pathway involving SIRT1 and multipotent mesenchymal stromal/stem cell-mediated AMP-activated protein kinase (AMPK) α in macrophages, resulting in deacetylation of spliced X-box-binding protein 1 and subsequent inhibition of the NLRP3 inflammasome.
  It was reported that mitophagy/autophagy blockade leads to the accumulation of damaged mitochondria generating ROS, and this in turn activates the NLRP3 inflammasome.^99^ For instance, a study carried out by Zhao et al. suggested that the mechanism of action by which SIRT3 protects against tissue damage involved the attenuation of ROS production and reduction of NLRP3 activity, resulting in the inhibition of oxidative stress and the downregulation of proinflammatory cytokines.^64^ However, little information is available on the relationship between SIRT3 and NLRP3; thus, further research is necessary to determine whether SIRT3 has a direct effect on the NLRP3 inflammasome.
3. **The effect of SIRTs targeting noncoding RNAs on the inflammatory pathway**
  Current studies have mainly elucidated the role of the SIRT family in the inflammatory response. However, exploration of the molecular mechanism underlying how SIRTs affect inflammation is still limited, especially studies examining the interaction of SIRT1 with noncoding RNAs. For example, microRNAs (miRNAs) can negatively regulate inflammation by repressing SIRT1. Downregulation of miRNAs such as miR-217 and miR-543 mitigated the inflammatory response by regulating the SIRT1/AMPK/NF-κB signaling pathway.^100^ In the same way, miR-378 reduced SIRT1 activity and facilitated the inflammatory pathway involving NF-κB-TNFα by targeting 5'-AMPK subunit gamma-2.^101^ In addition, the RNase monocyte chemoattractant protein-induced protein 1 alleviated inflammatory responses by promoting the expression of SIRT1 mediated via miR-9.^102^ Furthermore, SIRT1 targets the p53/miR-22 axis to suppress inflammation, cyclooxygenase (COX)-2 and inducible nitric oxide synthase (iNOS) expression.^103^ These studies suggest that the regulation of SIRTs by noncoding RNAs may be a promising therapeutic strategy for inflammation-related diseases.

##### Conclusion

In summary, the SIRT family is involved in inflammation via various mechanisms. Although the details of SIRT-dependent regulation of inflammation are becoming clear, many unanswered questions remain. For example, further studies are needed to explore whether depletion of SIRTs is a common pathological change in the occurrence and development of inflammation-related diseases. Further attention is also needed to resolve some of the conflicting data and better understand the critical role of the SIRT family in the inflammatory response. The contradictory roles of the SIRT family in inflammation may result from their regulation of common signaling pathways under specific pathologic conditions. While determining what role the SIRT family plays in inflammation, researchers should also target its mechanism of action in order to lay the foundation for subsequent clinical translational studies. To summarize, we have focused on introducing relevant studies and the beneficial effects of the SIRT family through its regulation of inflammatory pathways, providing an important reference point for future studies.

#### The role of SIRTs in metabolism

**Fig. 3:**
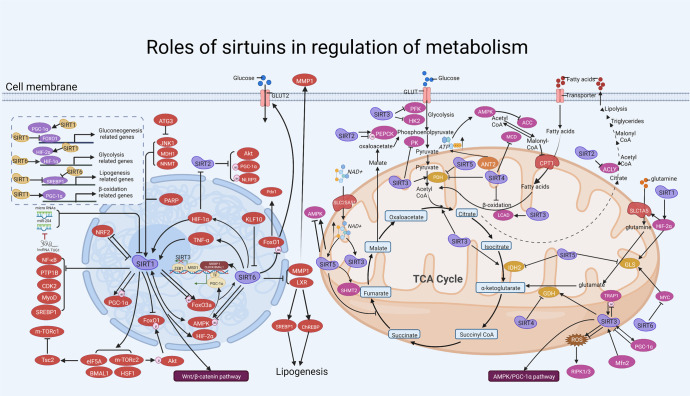
Overview of the roles of SIRTs in cell metabolism. SIRTs participate in glucose metabolism, lipid metabolism, and other metabolisms via interacting with metabolism-related genes and enzymes. (i) In the nuclear, SIRT1 and SIRT6 activate the transcription factors HIF2α and HIF1α respectively through different manners and subsequently improve glycolysis. Besides, SIRT1 regulates gluconeogenesis by activating PGC1α and inhabiting FOXO1, thereby affecting the transcriptional activation of their target genes. SIRT1 also promotes fatty acid oxidation by activating PGC1α and promoting the expression of target genes. Besides the positive regulation, SIRT1 and SIRT6 suppress SREBP1 and transcriptionally represses lipogenesis. (ii) In cytoplasm, SIRT2 deacetylates and activates the rate-limiting enzyme PEPCK and promotes gluconeogenesis during low nutrient condition. Moreover, SIRT2 inhabits ACLY and deters lipid synthesis. (iii) Regarding SIRTs in mitochondria, SIRT4 and SIRT5 reduces PDH activity which converts pyruvate to acetyl CoA. Both SIRT3 and SIRT4 target GDH, but their enzymatic activities are opposite. Besides GDH, SIRT3 also improves IDH2 and LCAD activity, thus enhancing cellular respiration and stimulating β-oxidation of fatty acids. Moreover, SIRT5 represses IDH2 activity and may disrupt glutamine metabolism through GLS. Activation and inhibition effects are displayed in “arrows” and “inhibitors”, respectively. https://biorender.com. ACC acetyl-CoA carboxylase, ACLY ATP citrate lyase, ANT2 adenine nucleotide translocase 2, Bmal1 brain-muscle-Arnt-like protein-1, CDK2 cyclin-dependent kinase 2, ChREBP carbohydrate response element-binding protein, CPS1 carbamoyl phosphate synthetase 1, CPT1 carnitine palmitoyl transferase 1 A, eIF5A eukaryotic initiation factor 5A, GDH glutamate dehydrogenase, GLUT glucose transporter, HIF1/2α hypoxia-Inducible Factor-1/2α, HK2 hexokinase 2, HSF1 heat shock factor 1, IDH2 isocitrate dehydrogenase 2, LCAD long chain acyl CoA dehydrogenase, MCD malonyl CoA decarboxylase, MBD1 methyl-CpG-binding domain protein 1, MDH1 malate dehydrogenases 1, m-TORc1/2 mTOR complex 1/2, MyoD myogenic differentiation factor, NNMT nicotinamide N-methyl transferase, PARP poly (ADP-ribose) polymerase, PDH pyruvate dehydrogenase, PEPCK1 phosphoenolpyruvate carboxykinase, PFK phosphofructokinase-1, PK pyruvate kinase, PTP1B protein-tyrosine phosphatase 1B, RIPK1/3 receptor interacting protein kinases 1/3, SLC1A5 solute carrier family 1 member 5, SREBP1 sterol regulatory element binding protein 1, TRAP1 tumor necrosis factor receptor-associated protein 1, Tsc2 tuberous sclerosis complex 2, ZEB1 zinc finger E-box binding homeobox 1

Metabolism is the general term for a series of ordered chemical reactions that take place in the body to sustain life.^104,105^ These processes allow organisms to grow and reproduce, maintain their structure and respond to the external environment.^106–108^ Metabolism mainly includes glucose metabolism and lipid metabolism.^104,109^ Many metabolic processes occur in the mitochondria where SIRT3–5 proteins are located. In addition, SIRT proteins located in the nucleus may participate in regulating several metabolism-related genes.^109,110^ In this section, we focus on the SIRT proteins and their roles in maintaining metabolic homeostasis by participating in the regulation of glucose, glutamine, and lipid metabolism (Fig. 3).

##### The effect of SIRTs on glucose metabolism

Glucose metabolism refers to a series of complex chemical reactions after glucose, glycogen and other substances enter the body, including anaerobic glycolysis of glucose, aerobic oxidation, synthesis and decomposition of glycogen, and gluconeogenesis.^111,112^ Abnormal glucose metabolism and insulin resistance might cause metabolic diseases such as diabetes.^113–115^ The roles of SIRTs in glucose metabolism have been established. For example, SIRT1 is a key positive regulator of systemic insulin sensitivity and regulates pancreatic insulin secretion, thus contributing to increased systemic insulin sensitivity, which triggers glucose uptake and utilization.^116–118^ Mechanistically, SIRT1 participates in the regulation of glucose metabolism by upregulating AMPK, and activation of AMPK can ameliorate the glucose metabolic imbalance.^116,119^ Upregulated SIRT1 may reverse the development of diabetes by targeting the AMPK/acetyl CoA carboxylase signaling pathway.^117^ Similarly, decreased levels of SIRT1 may lead to AMPK deficiency, thereby impairing the improvement in glucose tolerance.^119^ Meanwhile, there are an interdependent relationship between AMPK and SIRT1,^120,121^ and activation of SIRT1 and its downstream signaling pathways could also be improperly triggered in AMPK-deficient states.^121^ Additionally, SIRT1 increases insulin sensitivity and lowers blood sugar by downregulating protein tyrosine phosphatase 1B, a key negative regulatory protein in the insulin signal transduction pathway.^118^ Thus, high expression of SIRT1 is benefit for maintaining blood sugar stability via the regulatory proteins of insulin signaling. However, the relationship between SIRT1 and other molecules (e.g., AMPK and protein tyrosine phosphatase 1B) that are closely associated with blood glucose regulation is still worth further exploration.

SIRT1, SIRT3, and SIRT6 also participate in glucose metabolism. The limited whole-body benefit of increasing hepatic SIRT3 during the development of diet-induced insulin resistance, which can be considered a pre-diabetic state, has also been demonstrated.^122^ Mechanistically, SIRT3 negatively regulates aerobic glycolysis by inhibiting hypoxia-inducible factor 1α (HIF-1α).^123^ SIRT6 takes part in the maintenance of glucose metabolic homeostasis in the whole body and in local tissues such as liver and skeletal muscle.^124,125^ For instance, SIRT6 in pancreatic β cells deacetylated FoxO1 and subsequently increased the expression of glucose-dependent transporter 2 to maintain the glucose-sensing ability of pancreatic β cells and systemic glucose tolerance.^126^ Improvement in SIRT6-mediated insulin signaling transduction has been reported in the liver of obese rats after exercise.^127^ Also, enhancement of insulin sensitivity in skeletal muscle and liver by physiological overexpression of SIRT6 has been described,^128^ suggesting potential functions of SIRT6 in glucose metabolism.

Finally, direct and indirect involvement of SIRTs in glucose metabolism may provide new insights into therapeutic targets for the treatment of abnormal glucose metabolism in the future. This may help reduce the human disease burden related to glucose metabolism, where SIRT proteins may play an important role in overcoming glucose metabolic diseases at an earlier time point.

##### The effect of SIRTs on lipid metabolism

Lipid metabolism means that most of the fat ingested by the human body is emulsified into small particles by bile, and the lipase secreted in the pancreas and small intestine hydrolyzes the fatty acids in the fat into free fatty acids, after which hydrolyzed small molecules are absorbed by the small intestine into the bloodstream.^104,105,129^ Notably, the SIRT protein family is involved in lipid metabolism.^129,130^ For SIRT1, Qiang et al. found that SIRT1-dependent cAMP Response Element Binding protein (Creb) deacetylation regulates lipid metabolism.^131^ Mechanistically, Lys136 is a substrate for SIRT1-dependent deacetylation that affects Creb activity by preventing cyclic adenosine monophosphate (cAMP)-dependent phosphorylation, leading to the promotion of hepatic lipid accumulation and secretion. Moreover, SIRT1 activates AMPK, which leads to lipid-lowering effects in vitro and in vivo.^132^ SIRT2 prevents liver steatosis and lipid metabolic disorders by deacetylation of hepatocyte nuclear factor 4α.^133^ Additionally, SIRT3 acts as a bridge in the lipid metabolism pathway. For example, pancreatic SIRT3 deficiency promoted hepatic steatosis by enhancing 5-hydroxytryptamine synthesis in mice with diet-induced obesity.^134^ In addition, roles for SIRT5 and SIRT6 were identified in lipid metabolism.^135–138^ For instance, SIRT5 inhibited preadipocyte differentiation and lipid deposition by activating AMPK and repressing mitogen-activated protein kinase (MAPK) signaling pathways, which has been verified in obese mice.^135^ Compared with control wild-type mice, SIRT6-KO mice had a significant increase in both body weight and fat mass and exhibited glucose intolerance and insulin resistance.^138^ Mechanistically, SIRT6-KO decreased expression of the adiponectin gene and Akt in white adipose tissue, while expression of the thermogenic gene UCP1 was diminished in brown adipose tissue.^138^

##### The effect of SIRTs on other metabolism

SIRT3 and SIRT4 have been found to play roles in regulating glutamine metabolism. In detail, Gonzalez-Herrera et al. reported that loss of SIRT3 promoted glutamine use in nucleotide biosynthesis.^139^ Conversely, SIRT4 inhibited glutamine metabolism in colorectal cancer cells, thereby acting as a tumor suppressor.^140^ In addition, SIRT3 affected mitochondrial metabolic reprogramming by activating the AMPK/peroxisome proliferator-activated receptor-γ coactivator-1α (PGC-1α) pathway, thereby maintaining the stability of mitochondrial membrane potential as well as mitochondrial structure.^141^ Moreover, silencing SIRT6 influenced collagen metabolism in human dermal fibroblasts by affecting the synthesis and degradation of collagen.^142^

##### Conclusion

As shown in the previous findings, SIRT1, SIRT3, and SIRT6 have been more frequently studied than other SIRTs in regulating human body metabolism, mainly through their effect on glucose and lipid metabolism. However, only a few studies have focused on the roles of other SIRT proteins, in particular SIRT2 and SIRT7. In the future, research should be focused on the role of these other SIRTs in regulating different metabolism subtypes. Overall, clarifying the various participating mechanisms of SIRTs in metabolism might provide future new ideas for research and novel therapeutic targets for the treatment of abnormal metabolism, thereby lessening the burden imposed on society by human lipid metabolism-related diseases.

#### The role of SIRTs in oxidative stress

**Fig. 4:**
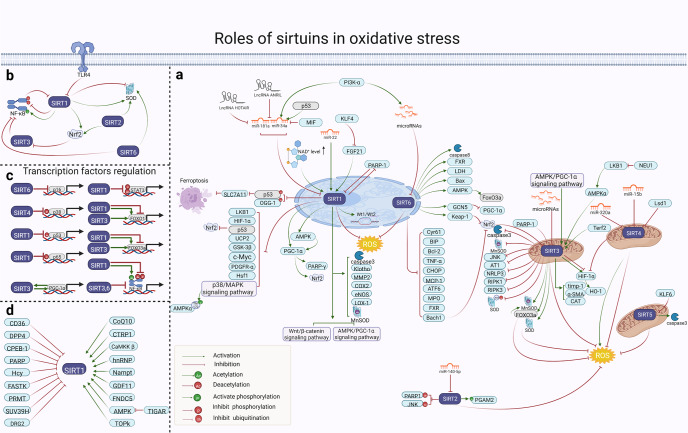
Overview of the roles of SIRTs in oxidative stress. **a** The overall roles of SIRTs in regulating cellular oxidative stress. The effect of SIRTs on oxidative stress is mainly via affecting the following proteins, mainly including Nrf2, FOXOs and SOD. SIRT1 and SIRT6 could indirectly affecting Nrf2 signaling, thereby regulating oxidative stress. SIRT3 activates FOXO3, which leads to increasement of MnSOD, allowing for the elimination of ROS. In addition, SIRT1, SIRT2, and SIRT6 could upregulate the expression of SOD, then reducing the ROS and inhibiting the oxidative stress; (**b**) The regulatory effects of SIRTs on main proteins in oxidative stress. SIRT1 downregulation by NF-κB leads to oxidative stress. Moreover, SIRT3 regulates ROS generation, causing suppression of NF-κB activation, and SIRT6 reduces NF-kB activation and represses oxidative stress. **c** The roles of SIRTs in regulation of transcription factors. SIRT1 increases the expression of FOXO1, reducing the production of ROS and oxidative stress. SIRT1 inhibits oxidative stress by deacetylating P53 protein. Besides, SIRT1 could activate PGC-1α and alleviate oxidative stress injury. **d** The proteins less studied that activate or inhibit SIRT1. Activation and inhibition effects are displayed in green and red arrows, respectively. https://biorender.com. AT1 angiotensin type 1, ATF6 activating transcription factor 6, Bach1 BTB domain and CNC homolog 1, BIP binding immunoglobulin protein, CD36 cluster of differentiation 36, CHOP C/EBP-homologous protein, CoQ10 coenzyme Q10, COX2 cyclooxygenase-2, CPEB-1 cytoplasmic polyadenylation element binding protein 1, DPP4 dipeptidyl peptidase-4, DRG2 GTP-binding protein 2, FASTK Fas-activated serine/threonine kinase, FNDC5 fibronectin type III domain-containing 5, GCN5 general control non-repressed protein 5, GDF11 Growth differentiation factor 11, Hcy homocysteine, hnRNP heterogeneous nuclear ribonucleoprotein F, HO-1 heme oxygenase 1, Keap-1 kelch-like ECH-associated protein 1, LDH lactate dehydrogenase, LOX-1 lectin-like oxidized low-density lipoprotein receptor-1, Lsd lysine-specific demethylase 1, MIF migration inhibitory factor, MPO myeloperoxidase, NEU1 neuraminidase 1, NRLP3 NOD-like receptor thermal protein domain associated protein 3, OGG-1 BER enzyme 8oxoG DNA glycosylase I, PDGFR-α platelet derived growth factor receptor α, PGAM2 glycolytic enzyme phosphoglycerate mutase 2, PRMT protein arginine methyltransferase, α-SMA smooth muscle alpha actin, TIGAR TP53-induced glycolysis and apoptosis regulator, timp-1 tissue inhibitor of metalloproteinase 1, TOPK T‑lymphokine‑activated killer cell‑originated protein kinase, UCP2 uncoupling protein 2, Wt1 Wilms' tumor 1, Wt2 Wilms' tumor 2

Oxidative stress is considered to be an important factor in cell damage and is usually caused by the overproduction of ROS. Under physiological conditions, ROS are produced at low levels and are scavenged by the endogenous antioxidant system. When ROS exceed the scavenging capacity, however, cellular oxidative stress damage occurs.^143^ Oxidative stress plays an important role in the pathological process of various diseases.^144^ Recently, accumulating studies have shown that the SIRT protein family participates in the process of oxidative stress. Notably, SIRT proteins contribute to cellular tolerance to oxidative stress by regulating many genes and their related signaling pathways (as shown in Fig. 4). Herein, we review the regulation of different target genes or proteins by SIRTs, with the aim of understanding their mechanistic effects in the process of antioxidant stress damage.

##### The interaction between SIRT1, SIRT3, SIRT6 and AMPK

AMPK, is a major regulator of metabolic homeostasis and is often activated under oxidative stress conditions such as ischemia and hypoxia.^145^ SIRT1 participates in regulating AMPK and its related pathways. For example, AMPK can be activated by liver kinase B1 (LKB1), the upstream regulator of AMPK, while activated AMPK reduces oxidative stress injury by promoting insulin sensitivity, fatty acid oxidation and mitochondrial biosynthesis to generate ATP.^146^ SIRT1 overexpression leads to the deacetylation of LKB1, the translocation of LKB1 from the nucleus to the cytoplasm, and the activation of AMPK to alleviate oxidative stress.^147^ Additionally, SIRT1 lowers LKB1 activation in the liver, which subsequently abrogates Thr172-AMPKα phosphorylation, thereby increasing oxidative stress in severe acute hypoxia.^148^ It can be seen that SIRT1 may activate AMPK by regulating LKB1, thereby resisting oxidative stress damage and promoting cell survival.

In addition to the role of SIRT1 on AMPK, SIRT3 and SIRT6 can also interact with AMPK to exert an anti-oxidative effect on stress injury. Deficiency of AMPKα resulted in elevated expression of SIRT3, which modulated oxidative stress in heart tissue both in vitro and in vivo .^149^ It has also been shown that the AMPK activated SIRT3, limited oxidative stress and improved mitochondrial DNA integrity and function.^150^ In addition, SIRT3 reduced ROS and lipid peroxidation by improving mitochondrial function via deacetylation of LKB1 and activation of AMPK.^151^ As previously mentioned, a feedback loop may exist between AMPK and SIRT3. SIRT6 also promoted AMPK expression, thus upregulating antioxidant-encoding gene expression of manganese superoxide dismutase (MnSOD) and catalase (CAT), thereby suppressing oxidative stress.^152^ In brief, SIRT1, SIRT3 and SIRT6 act to counter oxidative stress by directly or indirectly interacting with AMPK. However, additional studies are required to clarify the relationship between other SIRT proteins and AMPK pathway under oxidative stress.

##### The effect of SIRT1, SIRT 2, and SIRT6 on Nuclear erythroid 2-related factor 2 (Nrf2)

Nrf2 is a leucine transcription factor that plays extremely important roles in antioxidant response element (ARE)-dependent transcriptional regulation of defense genes. When stimulated, Nrf2 dissociates from suppressor protein Keap1 in the nucleus and interacts with AREs to regulate the expression of antioxidant genes, suggesting a close association between Nrf2 and oxidative stress.^153^ Notably, SIRTs including SIRT1, SIRT2 and SIRT6 can activate Nrf2, regulate antioxidant gene expression, and thus fight oxidative stress damage. For example, SIRT1 activated Nrf2 by changing the structure of Keap1, leading to Nrf2 nuclear transfer and promoting the expression of antioxidant genes, such as glutathione S transferase and glucuronyl transferase.^154,155^ In addition, SIRT2 was downregulated in the spinal cord after peripheral nerve injury, which subsequently inhibited Nrf2 activity, leading to increased oxidative stress.^156^ The overexpression of SIRT6 in the brain through in vivo gene transfer enhanced Nrf2 signaling and reduced oxidative stress.^157,158^ SIRT6 protected human lens epithelial cells from oxidative damage via activation of Nrf2 signaling.^159^ Furthermore, SIRT6 protects cells against hydrogen peroxide-induced oxidative stress by promoting Nrf2/ARE signaling.^160^ Therefore, SIRTs can activate Nrf2, regulate antioxidant gene expression, and thus fight oxidative stress damage.

##### The effect of SIRT1 and SIRT3 on FoxOs

A family of SIRT targets are class O mammalian forkhead transcription factors (FoxO1, FoxO3, FoxO4 and FoxO6) which participate in regulating oxidative stress. FoxO1 can scavenge excessive ROS through the regulation of downstream target genes such as MnSOD and CAT, and thus reduce cellular oxidative stress damage. SIRT1 alleviates oxidative stress by controlling nuclear shuttling and transcriptional activity of FoxO1 and FoxO3a. For instance, SIRT1 induced the transfer of FoxO1 to the nucleus and increased the level of FoxO1 protein in adipocytes, reducing the production of ROS and oxidative stress.^161^ Moreover, SIRT1 promoted early-onset age-related hearing loss by suppressing FoxO3a-mediated oxidative stress resistance in vivo.^162^ Apart from SIRT1, SIRT3 has also been shown to participate in the regulation of oxidative stress via FoxO3.^163,164^ Mechanistically, SIRT3 activated FoxO3 gene expression, which increased transcription of MnSOD and CAT, enabling the elimination of ROS.^165,166^ The aforementioned studies show that SIRT1 and SIRT3 can interact with FoxOs to counteract oxidative stress.

##### The effect of SIRT1 and SIRT3 on PGC-1α

PGC-1α is a coactivator of peroxisome proliferator-activated receptor-γ, which can act to block oxidative stress damage by scavenging excess ROS, inducing antioxidant enzyme expression and maintaining mitochondrial function.^167^ SIRT1 can activate PGC-1α through deacetylation, scavenge ROS caused by oxidative stress, and alleviate oxidative stress injury. Activation of the SIRT1-PGC-1α axis implies activation of antioxidant defense mechanisms, alleviating mitochondrial oxidative stress.^168–170^ Additionally, PGC-1α and SIRT3 can interact directly. PGC-1α increased respiratory capacity and reduced oxidative stress through SIRT3-mediated reduction of mitochondrial ROS.^171,172^ Furthermore, loss of SIRT3 resulted in the expression of PGC-1α, which produced a decrease in mitochondrial respiration. Inhibition of SIRT3 reduced PGC-1α expression and mitochondrial function, thereby lowering oxidative stress resistance.^173,174^ Thus, both SIRT1 and SIRT3 may interact with PGC-1α in order to resist oxidative stress damage.

##### The effect of SIRT1 and SIRT6 on p53

p53 is a stress response transcription factor and was the earliest discovered physiological substrate of SIRT1. p53 can promote oxidative stress injury by regulating different target proteins and further induce cellular responses.^175^ p53 exerted pro-oxidant activity and promoted oxidative damage by regulating its transcriptional targets, including p53-inducible gene 3, glutathione/NADH, p-FoxO3a and B-cell lymphoma -2-associated-X-protein (Bax).^176^ In contrast, p53 can act as an antioxidant factor to suppress oxidative stress by regulating several redox-related proteins, such as MnSOD, glutathione peroxidase 1, and Jun N-terminal kinase (JNK).^176^ When cells are under oxidative stress, multiple sites in the N-terminal of p53 are phosphorylated and multiple lysine sites in the C-terminal are acetylated.^177^ SIRT1 has a negative regulatory effect on p53; for example, depletion of SIRT1 abolished the increase in oxidative stress induced by p53 acetylation in THP-1 cells.^178^ SIRT1 activation also reversed p53 expression and accumulation brought on by H_2_O_2_-induced oxidative stress.^179^ The small molecule activator SRT2104 enhanced renal SIRT1 expression and activity and deacetylated p53, resulting in activation of antioxidant signaling.^180^ As for the role of SIRT6 in oxidative stress, relevant studies have been limited. For instance, SIRT6 protected cardiomyocytes by inhibiting p53/Fas-dependent cell death and augmenting endogenous antioxidant defense mechanisms.^181^ Hence, SIRT1 and SIRT6 can inhibit p53 activity through deacetylation and reduce oxidative factor expression, promoting resistance to oxidative stress injury.

##### The effect of SIRT1, SIRT3, and SIRT6 on NF-κB

NF-κB is a nuclear transcription factor. Activated NF-κB factors promote the production of ROS that damage tissues and organs.^182^ When oxidative stress occurs, enhanced ROS activity can stimulate the activation of NF-κB and induce the expression of ICAM-1 and monocyte chemotactic factor 1, which further activate NF-κB and lead to oxidative stress.^183^ SIRTs inhibited transcription by deacetylating the NF-κB subunit Rel/p65, reducing the production of oxygen radicals.^79^ SIRT1, SIRT3 and SIRT6 inhibited the transcriptional activity of NF-κB through deacetylation, thereby resisting oxidative stress injury. For example, downregulation of SIRT1 protein levels by NF-κB led to oxidative stress.^184^ In addition, SIRT3 regulated ROS generation, causing suppression of NF-κB activation and oxygen radicals.^185^ Moreover, loss of SIRT6 in cutaneous wounds aggravated the proinflammatory response by increasing NF-κB activation and promoting oxidative stress.^186^ Therefore, SIRT1, SIRT3, and SIRT6 can block oxidative stress damage by inhibiting NF-κB activity.

##### The effect of SIRTs on oxidative stress through other pathways

Many molecules are upstream regulators of SIRTs and have a regulatory effect on them under oxidative stress. For example, the expression of SIRT1 and SIRT6 was decreased by oxidative stress-dependent miR-34a activation in epithelial cells.^187^ SIRT5 was upregulated by Krüppel-like factor (KLF) 6 silencing, thereby reducing oxidative stress.^188^ Meanwhile, SIRTs target many downstream factors, such as HIF-1α and endothelial nitric oxide synthase (eNOS), and then participate in regulating oxidative stress. Activation of HIF-1α is associated with oxidative stress and can regulate ROS formation through direct or indirect effects.^189^ For example, SIRT4 reduced the accumulation of ROS by inhibiting HIF-1α, which is also an important mechanism underlying SIRT4 activity in oxidative stress.^190,191^ In addition, eNOS dysfunction in an oxidative stress environment led to increased generation of ROS. SIRTs play important roles in regulating the activity of eNOS as well. For instance, upregulation of SIRT1 reduced eNOS acetylation (inactive state) and enhanced eNOS phosphorylation (active state).^192^ Activation of the SIRT1/eNOS pathway has been found to reduce ROS production by inhibiting NF-κB expression.^193^ In brief, the mechanisms by which SIRTs regulate oxidative stress are diverse, and there are many more regulatory pathways that need to be verified.

##### Conclusion

Together, these aforementioned studies reflect the importance of the SIRT protein family in oxidative stress and can be expected to stimulate future research in order to decipher the SIRT protein mechanisms. As summarized in Fig. 4, SIRTs are involved in the regulation of redox homeostasis and oxidative stress involving many key genes and molecules. Indeed, SIRTs play important roles in maintaining intracellular homeostasis which keeps cells healthy, making them ideal for redox regulation studies. Additionally, SIRTs enhance intracellular homeostasis by acting synergistically through different mechanisms.

Further in-depth studies are needed to identify and elucidate the exact role of each SIRT and to determine whether different SIRTs have functional redundancy or overlapping roles in homeostasis, which may be important for regulating oxidative stress in cells and important pathological manifestations. SIRTs should be developed as modulators of redox-related diseases, and may also provide a mechanistic basis for the development and discovery of antioxidants. Given the interest in SIRTs as drug targets and their redox importance, studies addressing these questions may also provide therapeutic opportunities for the treatment of metabolic, age-related and other redox-related diseases.

#### The role of SIRTs in cell apoptosis

**Fig. 5:**
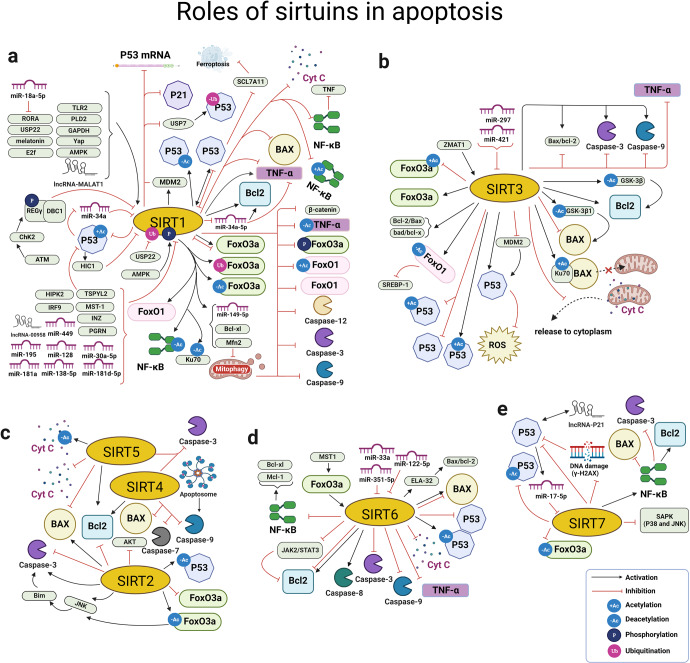
Overview of the roles of SIRTs in apoptosis. SIRT protein family has functions in both physiological conditions and diseases by regulating the acetylation modification and/or influencing various apoptosis-related proteins by crosstalk of pathways. Meanwhile, they can also be regulated by the molecules in the aforementioned process, such as microRNA, FoxO1, FoxO3a, TNF-α and NF-κB. **a** The roles of SIRT1 in regulating apoptosis by targeting apoptosis-related proteins and pathways; (**b**) The roles of SIRT3 in regulating apoptosis by targeting apoptosis-related proteins and pathways; (**c**) The roles of SIRT2, SIRT4 and SIRT5 in regulating apoptosis by targeting apoptosis-related proteins and pathways; (**d**) The roles of SIRT6 in regulating apoptosis by targeting apoptosis-related proteins and pathways; (**e**) The roles of SIRT7 in regulating apoptosis by targeting apoptosis-related proteins and pathways. https://biorender.com. ATM ataxia telangiectasia mutated, Cyt C cytochrome c, ELA elabela, GAPDH glyceraldehyde 3-phosphate dehydrogenase, HIC1 hypermethylated in cancer-1, HIPK2 homeodomain-interacting protein kinase-2, INZ inauhzin, JAK2 janus kinase 2, MALAT1 metastasis-associated lung adenocarcinoma transcript 1, Mcl-1 myeloid cell leukemia 1, MicRNA microRNA, MST1 mammalian sterile 20-like kinase 1, PLD2 phospholipase D2, RORA retinoid-related orphan receptor α, TSPYL2 testis-specific protein y-encoded-like 2, Yap yes-associated protein, ZMAT1 zinc finger matrin-type 1

Cell apoptosis is an active form of cell death that involves programmed cellular machineries leading to progressive self-destruction of the cell.^194^ As a type of programmed cell death, apoptosis is a basic cellular mechanism and may occur in numerous diseases. Notably, one of the most extensive biological functions regarding the SIRT protein family is participation in the process of cell apoptosis. The SIRT protein family has functions in both physiological conditions and diseases by regulating the acetylation modification and/or influencing various apoptosis-related proteins by pathway crosstalk, and thus takes part in the pathogenesis of many diseases including cancer, CVDs and others (Fig. 5).^195,196^

##### The effect of SIRTs as histone deacetylases on apoptosis

Histones are the major protein components of chromatin, serve as spools around which DNA is wound, and play roles in gene regulation. The SIRT family-dependent epigenetic regulation of histone acetylation is an important link in the regulation of apoptosis.^197^ For example, SIRT1 can reduce the acetylation levels of histones in the promoters of genes, e.g., AR, BReast-CAncer susceptibility gene 1(BRCA1), ERS1, ERS2, EZH2 and EP300, which ultimately affected cancer cell apoptosis.^197^ Additionally, SIRT6 links histone H3K9 deacetylation to NF-κB-dependent gene expression and organismal life span.^80^ At the molecular level, SIRT6 binds to the promoters of extracellular signal-regulated kinase (ERK) 1 and ERK2 genes, and deacetylates histone H3K9, thereby inhibiting ERK1/2 expression.^198^ Moreover, SIRT6 induced the expression of GATA binding protein 5 (GATA5) through inhibition of Nkx3.2 transcription by deacetylating histone H3K9, thereby regulating GATA5-mediated signaling pathways to prevent endothelial injury.^199^ These studies have demonstrated the critical role of the SIRT protein family in regulating apoptosis. However, additional studies have found that the SIRT protein family regulates other novel modification types of histones, for example, sumoylation^200^ and ubiquitination.^201^ Whether these new types of histone modification participate in cell apoptosis remains largely unknown, which may be a new direction for further research.

##### The effect of SIRT1 on apoptosis by targeting apoptosis-related proteins and pathways

Among the SIRT protein family, SIRT1 is the most widely studied protein, especially in regulating cell apoptosis. A variety of transcription factors, including p53, NF-κB and FoxO, which act downstream of SIRT1, are closely related to cell apoptosis.^103,202–204^ Therefore, we focus here on how SIRT1 participates in regulating these three proteins and their related pathways.

1. **SIRT1 mediates p53-dependent apoptosis by suppressing acetylated p53**
  As first discovered with non-histone targets of SIRT1, p53 plays a central role in the prevalence of diseases related to apoptosis.^205–207^ SIRT1 regulates p53 deacetylation, which is associated with the apoptosis-inhibiting signaling pathway, mainly including the p53-induced death domain protein Pidd,^208^ p21, Bax/Bad and caspases.^209^ For example, Zeng et al. reported that an extract of *Anoectochilus roxburghii* flavonoids reduced neuron apoptosis by positively regulating SIRT1 expression, thereby reducing expression of the apoptosis-related molecules p53, p21 and caspase-3, while increasing the ratio of B-cell lymphoma (Bcl)-2/Bax.^210^ SIRT1 also participated in the regulation of p53 protein through direct deacetylation. For example, SIRT1 deacetylating p53 at Lys379 inhibited p53-dependent apoptosis.^211^ In addition, SIRT1 can regulate the p53 signaling pathway by targeting proteins. The overexpression of SIRT1 resulted in markedly reduced mRNA and protein expression levels of p53 signaling pathway-related molecules (including p53 and Bax) in vitro, but increased Bcl mRNA and protein expression.^212^ p53 expression gradually decreased with increasing SIRT1 levels, thus indicating a gradual decrease in apoptosis.^213^ These findings thus show that SIRT1 inhibits apoptosis via inactivation of p53, suggesting a critical role for SIRT1 in regulating the p53 signaling pathway.
2. **The SIRT1/NF-κB pathway is mainly involved in inflammation-induced apoptosis**
  Regarding the mechanism underlying SIRT1 involvement in apoptosis, NF-κB (p65) acetylation was significantly increased after inhibition/deletion of SIRT1.^214^ A large number of studies have shown that SIRT1 mediates NF-κB pathway modulation to mitigate inflammasome signaling and cellular apoptosis.^203,214,215^ For example, SIRT1 overexpression promoted mouse B lymphocytes cell proliferation, inhibited apoptosis, and upregulated pro-inflammatory cytokines by inhibiting the NF-κB pathway.^216^ Additionally, activating the NF-κB signaling pathway could ultimately induce apoptosis through regulation of the inflammatory process.^217^ Silencing interferon regulatory factor 9 curbed activity of the NF-κB signaling pathway by upregulating SIRT1, which further inhibited TNF-α induced changes in inflammatory cytokine secretion and promoted apoptosis.^218^ Therefore, it appears to be a double-edged sword that SIRT1 regulates NF-κB signaling to affect cellular inflammatory activation and apoptosis in different spatiotemporal dependencies.
3. **SIRT1 regulates apoptosis by the regulation of FoxOs**
  FoxO transcription factors can control cell survival by regulating the expression of genes involved in cell-cycle progression and promoting apoptosis.^219^ SIRT1 is a key regulator of cell defenses and survival in response to stress, which deacetylates and represses FoxO-dependent apoptosis.^219,220^ SIRT1 mediates cell apoptosis through the deacetylation of FoxO proteins including FoxO1,^221^ and upregulation of SIRT1 can inhibit apoptosis via the FoxO1/β-catenin pathway.^222^ Moreover, SIRT1, FoxO1, and sterol regulatory element binding protein-1 (SREBP-1) may act as a pathway and play crucial roles in apoptosis. At both the protein and mRNA levels, SIRT1 and SREBP-1 were upregulated in progestin-resistant cells, while FoxO1 was downregulated.^223^ Interestingly, SIRT1 may be a potential target for cross-regulation of post-transcriptional modifications. For example, acetylation was required for FoxO3-induced apoptosis through phosphorylated-FoxO3 (p-FoxO3) formation, while expression or activation of SIRT1 blocked p-FoxO3 formation and apoptosis.^224^ Deacetylation of FoxO3 by SIRT1 resulted in S-phase kinase-associated protein 2-mediated FoxO3 ubiquitination and degradation.^225^ These fine-tuning mechanisms of FoxO3 regulation modulated by PTMs may be a new method to regulate apoptosis in a coordinated manner. In summary, then, SIRT1 can regulate the activity of FoxO, thereby modulating the balance between anti-apoptotic and apoptotic genes.^226^
4. **miRNAs play important roles in the regulation of SIRT1**
  miRNAs, a subtype of non-coding RNAs, are small endogenous RNAs which can inhibit protein translation in apoptosis.^227^ Moreover, SIRT1 has been revealed to be targeted by miRNAs such as miR-34a, miR-181, miR-128, miR-449 and miR-30a-5p. For example, Yamakuchi et al. demonstrated a negative correlation between the expression of miR-34a and SIRT1, suggesting SIRT1 was a target of miR-34a.^228^ In addition, SIRT1 is a key player in the protection provided by miR-34a-5p inhibition during apoptosis.^229^ The overexpression of miR-181d-5p inhibited cell apoptosis and renal fibrosis in a mouse model by downregulating the SIRT1/p53 pathway.^230^ Furthermore, miR-181a increased FoxO1 acetylation and promoted granulosa cell apoptosis via SIRT1 downregulation.^231^ The previous study also suggested that miR-128 promoted apoptosis in human cancers via the p53/Bak axis.^232^ Upregulation of miR-128 promoted apoptosis in an epilepsy model in vivo and in vitro through the SIRT1/p53/Bax/cytochrome c/caspase signaling pathway.^233^ Other miRNAs, such as miR-449, have been investigated in a model of acute kidney injury model by detecting expression of its target SIRT1 and downstream factors p53/Bax.^234^ Inhibition of miR-449 elevated SIRT1 expression and inhibited acetylated p53 and Bax protein levels.^234^ Finally, miR-30a-5p targeted SIRT1 to activate the NF-κB/NLRP3 signaling pathway, resulting in cardiomyocyte apoptosis.^227^ These studies all demonstrate how miRNAs play important roles in the regulation of SIRT1, which should be further studied in various diseases in the future.
5. **Other regulatory molecules or factors acting on SIRT1**
  Upstream of SIRT1, in addition to miRNAs, a novel fibroblast growth factor 1 variant could counteract adriamycin-induced apoptosis by decreasing p53 activity via upregulation of SIRT1-mediated p53 deacetylation.^235^ There have also been a series of studies on the anti-apoptotic effect of melatonin which regulates SIRT1 in various physiological processes.^236–239^ Additionally, some chemicals or drugs, like cambinol and ginsenoside Rc, have been shown to inhibit or activate SIRT1 to regulate the apoptotic process.^240,241^ Given that the above-mentioned molecules can regulate SIRT1-related signaling pathways, SIRT1 may be a potential therapeutic target in the apoptotic response.

##### The effect of SIRT2 on apoptosis

Several previous studies have suggested that SIRT2 has complex regulating mechanisms promoting or inhibiting apoptosis.^242^ In contrast to SIRT1, SIRT2 is predominantly a cytoplasmic protein and is able to deacetylate several cytoplasmic substrates,^243^ including p53,^244^ NF-κB,^245^ and FoxO3.^246^ In terms of its anti-apoptotic effects, SIRT2 downregulation alone is sufficient to cause apoptosis, and SIRT2 depletion leads to p53 accumulation causing activation of the p38 MAPK in cancer cell lines such as HeLa, but not in normal cells.^247^

On the other hand, SIRT2 can promote apoptosis mediated by the caspase, Bcl2/Bax and FoxO pathways. For example, She et al. demonstrated that the SIRT2 inhibitor AGK2 effectively reduced the levels of phospho-JNK and FoxO3a.^248^ As JNK is a well-known regulator of apoptosis, protein downregulation will lead to attenuation of the subsequent signaling cascade involving Bim, and eventually leads to suppression of the caspase cascade.^248^ In addition, SIRT2 overexpression induces cellular apoptosis via upregulating cleaved caspase 3 and Bax and downregulating anti-apoptotic protein Bcl-2,^245^ suggesting the important role of SIRT2 in apoptosis. As for the FoxO-related pathway, FoxO3a, which is the immediate downstream target for SIRT2-driven deacetylation, is a promoter of apoptotic pathways in many diseases.^246,249^ SIRT2 activates FoxO3a by deacetylating it, which promotes the activation of the pro-apoptotic pathways Akt/FoxO3a and JNK, and thus increases apoptosis. Additionally, the administration of specific inhibitors of SIRT2 attenuates neuronal cell death under ischemic conditions in vitro and in vivo.^248^ The confusing role of SIRT2 in the process of apoptosis might thus be attributed to regulation of different pathways affected by different conditions, but more studies verifying SIRT2 functions in apoptosis will be needed in the future.

##### The effect of SIRT3-7 on apoptosis

1. **The critical roles of SIRT3-5 in regulating cell apoptosis**
  Three SIRT proteins, namely SIRT3–5, are localized to the mitochondrion, a dynamic organelle that functions as the primary site of endogenous apoptosis. Although mitochondrial SIRT proteins have not been as extensively studied as SIRT1, a growing body of studies have illustrated their importance in basic mitochondrial biology and apoptosis.
  SIRT3 plays a pro-apoptotic role in that glycogen synthase kinase-3 β (GSK-3β)/Bax, Bax/Bcl-2 and bad/Bcl-x/L ratios regulate apoptosis.^250,251^ SIRT3 overexpression promoted apoptosis by enhancing caspase 9 cleavage in hepatocellular carcinoma (HCC) cells,^252^ and SIRT3 depletion downregulated cleaved caspase 3 levels in lung cancer (LC) cells.^253^ In contrast, several studies have found that SIRT3 has an anti-apoptotic effect. SIRT3 deficiency resulted in significantly increased apoptosis, increased Bax and caspase 3 mRNA levels, and decreased Bcl-2 mRNA levels in septic mice,^254^ and also significantly increased caspase 3 expression in SIRT3-KO mice. Thus, SIRT3 plays different roles in different diseases, both pro- and anti-apoptotic. A typical example is when SIRT3 expression inhibited the growth of cancer cells by promoting apoptosis and necroptosis. In a stress injury disease model, SIRT3 inhibited apoptosis and exerted a protective effect against various stressors. For example, SIRT3 deficiency produced more melanocyte apoptosis by inducing severe mitochondrial dysfunction and cytochrome c release into the cytoplasm.^255^ However, more research is needed in the future to determine whether SIRT3 promotes or inhibits apoptosis of the caspase 3 pathway in different types of diseases.
  FoxO transcription factors are downstream targets of the serine/threonine protein kinase B/Akt, which promotes apoptosis signaling by affecting multiple mitochondria-targeting proteins.^256^ SIRT3 acetylation modulated FoxO1 and exerted apoptotic effects.^51^ In addition, SIRT3 post-translationally upregulated FoxO3a activity through deacetylation, dephosphorylation and deubiquitination to regulate apoptosis.^257^ Meanwhile, non-coding RNAs act as upstream regulators of SIRT3 to regulate apoptosis. For example, the miR-297 antagomir affected apoptosis by targeting SIRT3 to reduce the extent of IκBα and NF-κB phosphorylation and prevent activation of NLRP3.^258^ A similar study confirmed that SIRT3 was also a target of miR-421.^259^ Studies of the upstream and downstream regulatory mechanisms of SIRT3 regulating apoptosis are few and more research will be required in this area.
  There are only limited studies on SIRT4 and cell apoptosis, but these few have indicated that SIRT4 prevents apoptosis by affecting the ratio of pro-caspase 9/caspase 9 or pro-caspase 3/caspase 3, and by altering Bax translocation.^191,260^ In addition, SIRT5 participates in the regulation of apoptosis as a deacetylated protein and may have an effect on apoptosis-related proteins. For example, SIRT5 deacetylated cytochrome c, a protein of the mitochondrial intermembrane space with a central function in oxidative metabolism as well as in apoptosis initiation.^261^ SIRT5 overexpression ameliorated cytochrome c leakage and activation of caspase 3 to alleviate apoptosis.^262,263^ Thus, these data implicate mitochondrial SIRTs as effective in protecting against pathological injury and apoptosis by inhibiting the cytochrome c/caspase 3 apoptosis pathway. Such research may form the basis for future treatment for apoptosis. However, the number of related studies on SIRT4 and SIRT5 is still limited and need to be expanded.
2. **The role of SIRT6 and SIRT7 during apoptosis**
  At present, only a few studies have explored the role of SIRT6 and SIRT7, which could be a new research direction for the SIRT protein family. Both SIRT6 and SIRT7 mediate apoptosis by regulating p53.^264,265^ Furthermore, SIRT7 promoted cellular survival following genomic stress by attenuation of DNA damage and the p53 response.^266^ However, current studies on SIRT6 and SIRT7 are still in their infancy, and more research is needed in the future to explore their role in apoptosis.

##### Conclusion

In conclusion, one of the most extensive biological functions of the SIRT protein family is to participate in the process of apoptosis. As a family of bidirectional regulatory proteins, the function of SIRTs appears to be reversible depending on the cellular state. However, our current knowledge of SIRTs in apoptosis and its regulation is far from complete. More studies are needed in the future to explore the underlying molecular mechanisms of how the SIRT protein family is regulated in pathophysiological processes.

#### The role of SIRTs in autophagy

**Fig. 6:**
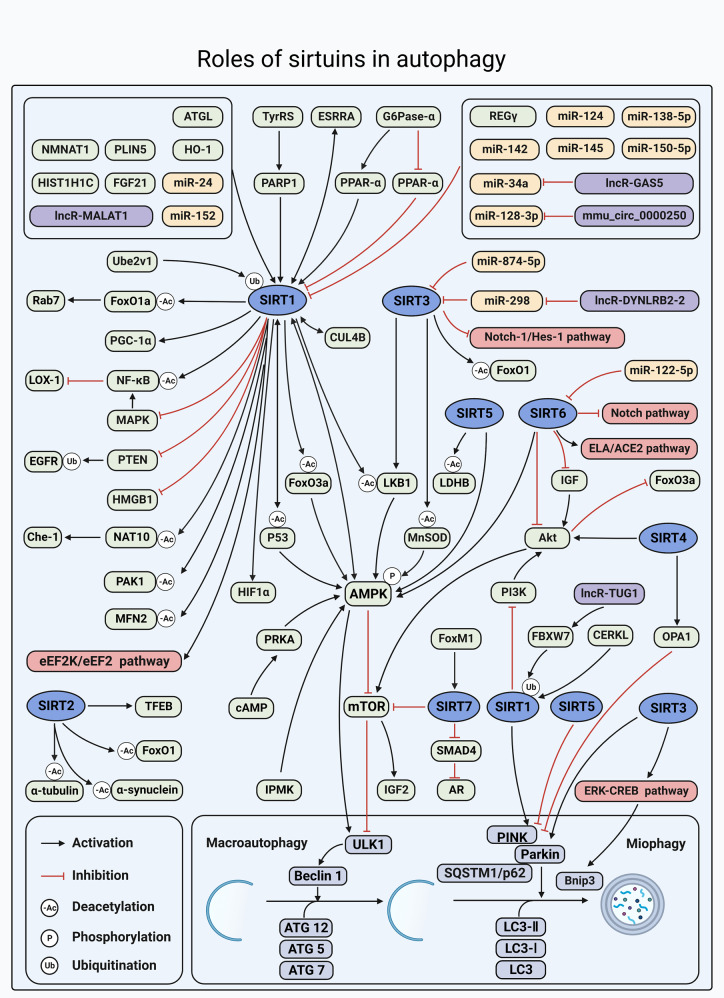
Overview of the roles of SIRTs in autophagy. SIRTs can regulate a series of substrates involved in the process of macroautophagy and mitophagy. Meanwhile, they can also be regulated by a series of molecules in the aforementioned process. SIRTs are all involved in the regulation of macroautophagy, of which AMPK/mTOR signaling is the most common pathway. In addition, SIRT1, SIRT3, SIRT4, and SIRT5 are also involved in PINK1/Parkin-mediated mitophagy or Bnip3-mediated mitophagy. https://biorender.com. ACE2 angiotensin-converting enzyme 2, ATGL adipose triglyceride lipase, Bnip3 BCL2 interacting protein 3, CERKL ceramide kinase-like protein; circ, circular RNA; CUL4B, cullin 4B, eEF2 eukaryotic elongation factor-2, eEF2K eukaryotic elongation factor-2 kinase, EGFR epidermal growth factor receptor, ESRRA estrogen-related receptor α, FBXW7 F-box and WD repeat domain-containing 7, FoxM1 forkhead box M1, G6Pase-α glucose-6-phosphatase-α, GAS5 growth arrest specific 5, Hes‑1 hairy and enhancer of split‑1, HIF1α hypoxia-inducible factor 1 α, HIST1H1C histone cluster 1 H1 family member c, IPMK inositol polyphosphate multikinase, LDHB lactate dehydrogenase B, lncR long non-coding RNA, miR miRNA, NAT10 nucleolar protein N-acetyltransferase 10, NMNAT1 nicotinamide mononucleotide adenylyltransferase 1, Notch‑1 Notch homolog 1, OPA1 optic atrophy 1, p53 tumor protein p53, PINK PTEN induced putative kinase, PLIN5 perilipin 5, PTEN phosphatase and tensin homolog, SQSTM1/p62 sequestosome 1, TFEB transcription factor EB, TUG1 taurine-upregulated gene 1, TyrRS tyrosyl transfer-RNA synthetase, Ube2v1 ubiquitin-conjugating E2 enzyme variant 1

Autophagy is a cell self-digestion process via lysosomes that clears cellular waste, including aberrantly modified proteins or protein aggregates and damaged organelles.^267^ Recent studies have illustrated the important roles of the SIRT protein family in the autophagic process. Therefore, in this section, we aim to review recent research on the relationship between the SIRT protein family and autophagy, and discuss possible regulatory roles of SIRT proteins in autophagy, as well as the conditions under which they participate in autophagy in a positive or negative manner (Fig. 6).

##### The effect of SIRT1 on autophagy

1. **SIRT1 regulates autophagy through deacetylation**
  SIRT proteins affect protein acetylation level, and this modification is closely involved in autophagy. There are complex roles for SIRT1-related deacetylation in the regulation of autophagy.^268,269^ For example, SIRT1 deacetylates autophagy-related proteins (such as Beclin-1 and microtubule-associated protein light chain 3 (LC3)) to promote autophagy. Deacetylation of Beclin-1 lysine residue by SIRT1 impairs autophagic flux; thus, autophagosome fusion with lysosomes is compromised.^270,271^ SIRT1 promotes autophagy of cancer cells by reducing acetylation of LC3.^272^ LC3 and autophagy related (Atg)7 deacetylation is disrupted in germ-cell-specific SIRT1 KO mice, which affects the redistribution of LC3 from the nucleus to the cytoplasm and activation of autophagy.^273^ Suppression of SIRT1 enhances acetylation level of unc-51 like kinase 1 (ULK1) and induces ROS-dependent autophagy.^274^ Therefore, SIRT1 could directly regulate autophagy through deacetylation of autophagic proteins.
  SIRT1 regulates autophagy via deacetylation of autophagy-related proteins as well as through deacetylation of mitochondrial proteins.^275^ Mitochondrial proteins participate in the process of mitophagy; a selective autophagic process that is critical for cellular homeostasis and eliminates dysfunctional mitochondria.^276^ For example, induction of autophagy by SIRT1/HIF-1α activation is a novel therapeutic option for peripheral nerve injury.^277^ SIRT1 activity is involved in mitochondrial biogenesis through PGC-1α and participates in the balance of autophagy regulatory proteins.^278^ Mitofusins2 (MFN2) is a mitochondrial fusion factor and increasing evidence has shown that it is involved in the regulation of autophagy.^279^ For example, MFN2 is deacetylated by SIRT1, and loss of SIRT1 causes a sequential chain of defective autophagy in an MFN2-dependent manner.^280^ Mechanistically, SIRT1 deacetylates K655 and K662 residues at the C terminus of MFN2, leading to autophagy activation.^281^
  In conclusion, SIRT1 acts on autophagy-related proteins and transcriptional factors mainly through modification of acetylation, and affects the occurrence or degradation of autophagosomes. However, there have been limited studies on other PTMs of SIRT1, and more research is needed to explore the regulatory mechanism of SIRT1 in the future.
2. **Upstream and downstream signaling pathway of SIRT1 in autophagy**
  AMPK is an evolutionarily conserved serine/threonine-protein kinase. Under various physiological and pathological conditions, AMPK acts as an activator of SIRT1 and is involved in the regulation of autophagy. For example, inositol polyphosphate multi kinase enhances autophagy-related transcription by stimulating AMPK-dependent SIRT1 activation.^282^ AMPK can also be activated as a downstream molecule of SIRT1. SIRT1 promoted autophagy via AMPK activation.^283^ Autophagy impairment is mediated by downregulation of SIRT1/FoxO3a/AMPK/ peroxisome proliferators-activated receptors (PPAR)-α signaling.^284^ The SIRT1 activator resveratrol increases cAMP content, expression of protein kinase A, as well as the activity of AMPK. Besides, resveratrol pretreatment reduces tumor necrosis factor α-induced inflammation and increases LC3B expression and sequestosome 1(SQSTM1)/p62 degradation in a concentration-dependent manner.^285^ Activation of the AMPK/SIRT1 pathway alleviates cell damage and promotes autophagic flux via downregulation of p62.^286^ Therefore, SIRT1 recognizes resveratrol-induced autophagy in vitro and in vivo via the cAMP/phosphorylated protein kinase A (PRKA)/AMPK/SIRT1 signaling pathway.^287,288^ AMPK acts as an upstream molecule to regulate expression of SIRT1 active agent. SIRT1 affects autophagy by binding to molecules directly. SIRT1 forms a molecular complex with Atg5, Atg7 and Atg8, and transiently increased expression of SIRT1 is sufficient to stimulate basal rates of autophagy.^289^ SIRT1 interacts with the Cullin 4B-Ring E3 ligase complex, which promotes autophagy of cancer cells.^290^ In conclusion, these molecules play important roles as the upstream or downstream of SIRT1 in the process of autophagy, and affect the occurrence and development of diseases.
3. **Noncoding RNAs in SIRT1-regulated autophagy**
  A variety of miRNAs have been found to affect autophagy by directly regulating expression of SIRT1. For example, miR-124 and miR-142 represses autophagy via targeting SIRT1 in cancer cells.^291^ Silencing of miR-150-5p increases autophagy by targeting the SIRT1/p53/AMPK pathway.^292^ miR-138-5p affects insulin resistance through inducing autophagy in HepG2 cells by regulating SIRT1, and overexpression of SIRT1 increases Beclin-1 and LC3 II/I levels, and the number of green fluorescent protein-LC3 dots, and decreases p62 level.^293^ miR-145 inhibition upregulates SIRT1 and attenuates autophagy via NF-κB-dependent Beclin-1.^294^
  Both long noncoding RNAs (lncRNAs) and circular RNAs (circRNAs) modulate autophagy associated with SIRT1. For instance, lncRNA metastasis-associated lung adenocarcinoma transcript 1 enhances ox- low-density lipoprotein (LDL)-induced autophagy through the SIRT1/MAPK/NF-κB pathway.^295^ lncRNA growth arrest specific 5 inhibits macroautophagy and forms a negative feedback regulatory loop with the miR-34a/SIRT1/mammalian target of rapamycin (mTOR) pathway.^296^
  In conclusion, SIRT1 is a key regulator of the autophagic process. Through its deacetylase activity, SIRT1 is involved in the regulation of different autophagic proteins from initiation to degradation. The level and function of SIRT1 are also regulated by many signaling pathways, such as AMPK. Some studies have shown the regulation of SIRT1 by ncRNAs. SIRT1-mediated autophagic dysregulation leads to progression of various diseases. In the future, we need more research evidence to improve and supplement the mechanism of SIRT1.

##### Effect of SIRT2 on autophagy

It has been indicated that SIRT2 controls the functional ability of the autophagic system through acetylation.^297^ Genetic manipulation of SIRT2 levels in vitro and in vivo modulates the levels of α-synuclein acetylation, its aggregation, and autophagy.^298^ SIRT2 loss of function either with AK1 (a specific SIRT2 inhibitor) or by SIRT2 KO recovers microtubule stabilization and improves autophagy.^299^ Additionally, SIRT2 directly binds to the 3'UTR of transcription factor EB and facilitates its mRNA stability. Transcription factor EB is a key transcription factor involved in the regulation of many lysosome-related genes and plays a critical role in the fusion of autophagosomes and lysosomes, indicating that SIRT2 modulates autophagic components.^300^ Although the precise mechanism is unresolved, SIRT2 plays a key role in regulating autophagy in certain diseases, and more research is needed.

##### Effect of SIRT3–5 on autophagy

As mitochondrial SIRTs (mtSIRTs) members, SIRT3–5 are all involved in regulating energy metabolism and metabolic homeostasis through regulation of mitophagy.^301,302^ SIRT3 regulates autophagy by activating different downstream signaling pathways. For example, overexpression of SIRT3 activates macroautophagy through activating the AMPK/ULK1 pathway.^301^ SIRT3 promotes expression of autophagic proteins Beclin-1 and LC3II via downregulation of the Notch-1/Hes-1 pathway.^303^ Functional studies showed that SIRT3 reversed Bnip3 expression and promoted Bnip3-required mitophagy activity via the ERK-CREB signaling pathway.^304^ SIRT3 is involved in the regulation of autophagy; however, its role as an autophagy regulator, particularly the molecular mechanism, remains poorly understood. One recent study found that SIRT3 was directly inhibited by miR-874-5p and promoted autophagy, while depletion of miR-874-5p inhibited autophagy.^305^ A related study indicated that SIRT3 regulated the LKB1/AMPK/mTOR autophagic signaling pathway through the lncRNA DYNLRB2-2/miR-298/SIRT3 axis.^306^ Compared with SIRT1, the studies related to autophagy in SIRT3 are still lacking.

Mitochondria represent a major source of ROS that affect mitochondrial function, resulting in autophagic clearance of damaged mitochondria.^183^ Localized in the mitochondria, SIRT4 regulates proteins involved in metabolic reactions, antioxidant pathways and autophagy, thus maintaining mitochondrial homeostasis.^307^ Overexpression of SIRT4 inhibits ROS production and autophagy by activating the Akt/mTOR signaling pathway.^308^ Furthermore, the SIRT4/optic atrophy 1 axis is causally linked to mitochondrial dysfunction and altered mitochondrial dynamics that translates into aging-associated decreased mitophagy.^301^ So far, there are few relevant studies on SIRT4 regulation of autophagy. Further studies need to explore the role of SIRT4 as an mtSIRT in mitochondrial processes, such as autophagy (mitophagy).

Unlike SIRT4, which inhibits autophagy, the role of SIRT5 in regulating autophagy is contradictory. In the case of inhibition of autophagy by SIRT5, mitochondrial size is increased and mitophagy decreased upon SIRT5 overexpression, whereas the opposite effect is observed in SIRT5-silenced cells or upon treatment with the SIRT5 inhibitor MC3482.^302^ However, SIRT5 could enhance autophagy in gastric cancer (GC) cells via the AMPK/mTOR pathway.^309^ Additionally, SIRT5-induced deacetylation of lactate dehydrogenase B triggers hyperactivation of autophagy; a key event in tumorigenesis.^310^ Succinyl-proteomics in brown adipose tissue of normal and SIRT5 KO mice. Overacylation due to SIRT5 deficiency leads to defective autophagy/mitophagy.^311^ Besides their functions in energy metabolism and mitochondrial respiratory chain complexes, all three mtSIRTs participate in the regulation of mitochondrial morphology/dynamics. They seem to promote mitochondrial fusion and/or inhibit fission, and thus might attenuate mitophagic clearance of dysfunctional mitochondria.^302^ At present, the mechanism of action of mtSIRTs on autophagy is still unclear.

##### Effect of SIRT6 on autophagy is mainly through inhibition of Akt-related pathway

SIRT6 is essential for the regulation of autophagy in cells. For example, overexpression of the *SIRT6* gene could inhibit apoptosis and induce autophagy, which might be involved in repairing kidney damage caused by lipopolysaccharide (LPS).^312^ Autophagy controls cellular senescence by eliminating damaged cellular components and is negatively regulated by Akt signaling through mTOR. SIRT6 overexpression induces autophagy via attenuation of insulin-like growth factor (IGF)/Akt/mTOR signaling.^313^ Lu et al. revealed that SIRT6 positively regulates autophagy in cardiomyocytes. Mechanistically, SIRT6 promotes nuclear retention of FoxO3 transcription factor via attenuating Akt signaling, which is responsible for autophagic activation.^314^ SIRT6 can be inhibited by upstream miR-122, resulting in a significant reduction in the levels of elabela, thereby preventing angiotensin II (Ang II)-mediated loss of autophagy.^315^ However, the mechanism of SIRT6 promotion of autophagy needs further study.

##### Effect of SIRT7 on autophagy needs further investigation

There are few studies about the effects of SIRT7 in autophagy. For example, silencing forkhead box M1 promotes apoptosis and autophagy through the SIRT7/mTOR/IGF2 pathway in GC cells.^316^ SIRT7 protects against chondrocyte degeneration in OA via autophagic activation.^317^ SIRT7 depletion significantly inhibits androgen-induced autophagy in LNCap and 22Rv1 cells (in vitro). SIRT7 plays an important role in tumor growth and metastases and immunohistochemical analysis of 93 specimens and bioinformatic analysis revealed that SIRT7 expression was positively associated with androgen receptor (AR) (in vivo).^318^ SIRT7 promotes prostate cancer autophagy indirectly via the AR signaling pathway.^318^ These results suggest that SIRT7 plays a positive role in promoting apoptosis. However, the number of studies on SIRT7 is still limited and further research is needed.

##### Conclusion

Autophagy is a highly conserved catabolic process and a major cellular pathway for the degradation of long-lived proteins and cytoplasmic organelles. Growing evidence has suggested that the SIRT protein family plays an important role in pathophysiology by mediating autophagy, maintaining cellular homeostasis, integrating cellular energy metabolism, and clearing damaged and waste cells. Although there is still a lot of work to be done, based on the current research, it is confident that the SIRT family might become a target for future research on autophagy. Investigating the exact mechanism of SIRT-mediated autophagy in different diseases is a new field to be explored in the future. Further studies should focus on the biological mechanism of SIRT co-regulating autophagy with various molecular signals and its role in different subcellular localization. Moreover, autophagy modulators of SIRTs may also provide new pharmacological targets.

#### Role of SIRTs in cell proliferation

**Fig. 7:**
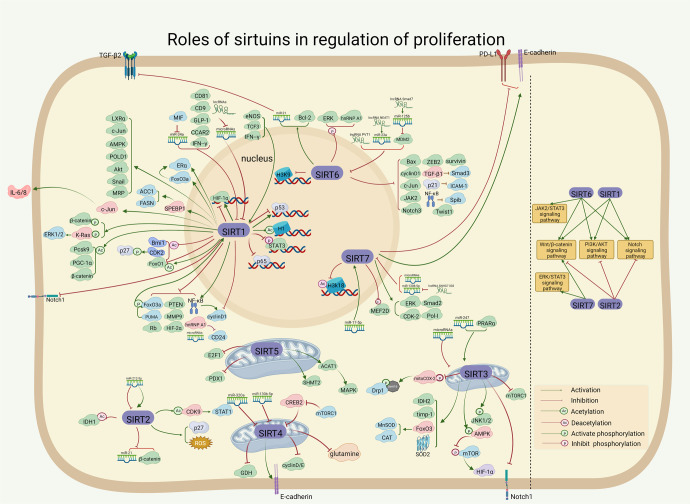
Overview of the roles of SIRTs in cell proliferation. (i) SIRTs participate in regulating cell proliferation by affecting a group of downstream proteins, including p53, p65, STAT3, FOXO1, AMPK, etc. (ii) SIRTs are also regulated by a series of ncRNAs and proteins, such as lncRNA PVT1, miR-34a, IFN-γ, MDM2, PRARα, eNOS, TCF3, etc, and subsequently promote or inhibit cell proliferation directly. (iii) In addition, SIRTs could activate or inhibit several signaling pathways, which perform important roles in cell proliferation, including JAK2/STAT3 signaling pathway, Wnt/β-catenin signaling pathway, PI3K/AKT signaling pathway, Notch signaling pathway, and ERK/STAT3 signaling pathway. Activation and inhibition effects are displayed in green and red arrows, respectively. https://biorender.com. ACAT1 acetyl coenzyme A acyltransferase1, Bmi-1 B-cell-specific Moloney murine leukemia virus integrationsite-1, CCAR2 cell cycle and apoptosis regulator protein 2, CDK9 cyclin-dependent kinase9, Drp1 dynamin-related protein 1, Erα estrogen receptor α, FASN fatty acid synthase, GLP-1 glucagon-like peptide-1, H1 histone1, HIF-2α hypoxia inducible factor-2α, K-Ras p21, MEF2D myocyte enhancer factor 2D, mitoCOX-2 mitochondria cyclooxygenase-2, MRP migration inhibitory-factor related protein, mTORC1 mTOR complex 1, Pcsk9 proprotein convertase subtilisin/kexin type 9, PD-L1 programmed death 1-ligand 1, POLD1 DNA polymerase delta 1, Pol-I DNA polymerase I, BBC3 Bcl-2 binding component 3, Rb retinoblastoma protein, SPEBP1 phosphatidylethanolamine binding protein 1, STAT1 signal transducer and activator of transcription 1, TCF3 transcription factor 3, Twist1 twist family bHLH transcription factor 1, ZEB2 zinc finger E-box binding homeobox 1

Cell proliferation is the process by which a cell grows and divides to produce two daughter cells.^319–321^ Cell proliferation leads to an exponential increase in cell number and is, therefore, a rapid mechanism of tissue growth.^321,322^ Cell proliferation requires both cell growth and division to occur at the same time, which is the basis of organismal growth, development, reproduction and inheritance (Fig. 7).^322–324^

##### Effect of SIRT1 on cell proliferation

SIRT1 is involved in regulating cell proliferation in a bilateral way by regulating protein expression and acetylation.^272,325^ The opposite effects of SIRT1 on cell proliferation have been observed among different cell types or the regulation of different downstream molecules. For example, SIRT1 promotes cell proliferation by regulating LC3 and retinoblastoma (Rb) acetylation. At the molecular level, SIRT1 promotes the proliferation of endometrial cancer (EC) cells by reducing acetylation of LC3.^272^ SIRT1 deacetylates Rb protein in the Rb/ E2F transcription factor 1 (E2F1) complex, leading to dissociation of E2F1 and enhanced oligodendrocyte progenitor cell proliferation.^326^ SIRT1 directly regulates expression of transcription factor proteins, such as E2F1 and p53, subsequently promoting macrophage and HCC cell proliferation, respectively.^327,328^

However, SIRT1 can have an antiproliferative role via regulating expression of key proteins related to cell proliferation, such as AMPK and signal transducer and activator of transcription 3 (STAT3). For instance, SIRT1 exerts antiproliferative effects via the AMPK/mTOR pathway in the context of mutant p53 in HCC cells.^329^ SIRT1 overexpression inhibits the proliferation of renal cancer cells, while inhibition of SIRT1 expression has the opposite effects.^325^ SIRT1 might serve an anticancer role in cancer cells by upregulating expression of downstream AMPK.^330^ SIRT1 also inhibits GC cell proliferation via the STAT3/matrix metalloproteinase (MMP)-13 signaling pathway.^331^ SIRT1 has both promotive and inhibitory effects on proliferation in different cells. However, more studies are still needed to elucidate the mechanisms and establish under which conditions SIRT1 promotes or inhibits cell proliferation.

##### Effect of SIRT2 on cell proliferation

Participation of SIRT2 in cell proliferation was identified by a series of studies.^332–335^ At the molecular level, SIRT2 regulates Myc and results in promotion of cell proliferation. For example, SIRT2 enhances N-Myc and c-Myc protein stability and promotes cancer cell proliferation.^332^ On the contrary, SIRT2 functions as an HDAC and inhibits proliferation of neuroblastoma cells, renal podocytes, and neuroblastoma cells.^336^ SIRT2 upregulation reduces cell proliferation in renal podocytes under high-glucose conditions.^337^ The opposite effect of SIRT2 on cell proliferation might be due to the different cell types, which might be the direction for future studies.

##### Effect of SIRT3 on cell proliferation

SIRT3, the major deacetylase in mitochondria, also plays a bilateral role in regulating cell proliferation. For instance, SIRT3 is responsible for hydroxymethyl-transferase 2 (SHMT2) deacetylation, and the conversion of serine and glycine accomplished by SHMT2 deacetylation in mitochondria is significantly upregulated to support cell proliferation.^338^ Chen et al. found that increased activity of SIRT3 contributed to decreased ROS levels and increased cell proliferation.^339^ Conversely, the expression of SIRT3 is upregulated by Profilin-1, and subsequently negatively regulates HIF-1α protein levels and suppresses cell proliferation.^340^

##### Effect of SIRT4 on cell proliferation

SIRT4 inhibits proliferation of several types of cancer cells. For example, SIRT4 inhibits the proliferation of cancer cells by inhibiting glutamine metabolism.^341,342^ In addition, cell proliferation due to repression of SIRT4 by the mTORC1 pathway has been identified.^343^ Moreover, SIRT4 is the molecular switch mediating cellular proliferation through glutaminase (GLS)-mediated activation of the Akt/GSK3β/CyclinD1 pathway; mechanically, SIRT4 suppression activates glutaminase, thereby initiating Akt activation.^344^

##### Effect of SIRT5 on cell proliferation

SIRT5 promotes cell proliferation in most conditions by regulating activity of signaling proteins or protein PTM. For instance, SIRT5 promotes cell proliferation by increasing activity of the MAPK pathway through acetyl-CoA acetyltransferase 1.^345,346^ Moreover, citrate synthase desuccinylation by SIRT5 promotes cancer cell proliferation.^347^ Similarly, SHMT2 desuccinylation by SIRT5 drives cell proliferation.^348^ In addition, SIRT5 regulates cell proliferation directly or indirectly by influencing expression of transcription factors, such as E2F1 and pancreatic and duodenal homeobox 1 (PDX1).^349^ However, SIRT5 suppresses the proliferation of pancreatic β-cells in vitro by downregulating transcription of PDX1 by deacetylating H4K16.^350^ In conclusion, SIRT5 has dual functions in regulating proliferation of different cell types. However, the distinct mechanism for the bilateral roles of SIRT5 is worth further exploration.

##### Effect of SIRT6 on cell proliferation

SIRT6 is also reported to regulate cell proliferation in a bilateral manner via influencing downstream molecules, such as AMPK, ERK, Wnt signaling and the MAPK pathway. SIRT6 promotes expression of COX-2 by repressing AMPK signaling, thereby increasing cell proliferation.^351^ Moreover, overexpression of SIRT6 promotes cell proliferation via upregulating he phosphorylation of ERK.^352^ In addition, SIRT6 deletion promotes hematopoietic stem cell proliferation through aberrant activation of Wnt signaling.^353^ Using genetic and biochemical studies in vitro and in human multiple myeloma xenograft models, Cea et al. found that SIRT6 depletion enhanced cell proliferation via upregulating expression of MAPK.^354^ In conclusion, SIRT6 has both promotive and inhibitory effects on cell proliferation. The different results of SIRT6 in regulating cell proliferation need further study.

##### Effect of SIRT7 on cell proliferation

Previous studies have shown that SIRT7 has a positive role in regulating cell proliferation.^355^ Upregulation of SIRT7 protects against the proliferation of vascular smooth muscle cells (VSMCs) in atherosclerosis.^355^ Similarly, SIRT7 deficiency attenuates VSMC proliferation, thus attenuating neointimal formation following vascular injury.^356^ Moreover, SIRT7 depletion inhibits cancer cell proliferation by suppressing AR signaling and activating p38MAPK.^318,357^

##### Conclusion

The direct and indirect involvement of SIRTs in proliferation could provide new ideas and evidence in support of potential research and as therapeutic targets. This might be meaningful for the treatment of abnormal proliferation in the future, thereby reducing the human disease burden related to proliferation. However, at present, research is still focused on the effect of SIRTs on carcinoma, and other molecular mechanisms of proliferation is less researched. Therefore, research on other molecular mechanisms of proliferation should be increased in the future. More evidence from in vitro and in vivo models for different kinds of diseases to confirm undefined molecular mechanisms of proliferation as yet is awaited.

#### Roles of SIRTs in cell migration and invasion

**Fig. 8:**
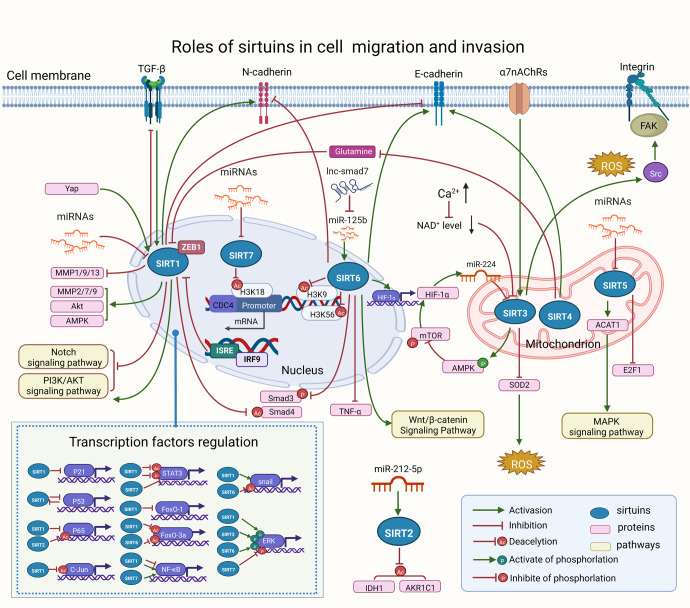
Overview of the roles of SIRTs in cell migration and invasion. SIRTs coordinate a multi-faceted regimen to control cell migration and invasion. In the nucleus, SIRT1, SIRT6, and SIRT7 may affect many key proteins, which also contain transcription factors, mainly involved in EMT process, TGF-β signaling, PI3K/Akt signaling, MMPs signaling, and AMPK signaling pathways, etc, thereby regulating cell migration and invasion. In the cytosol, SIRT2 could suppress cell migration and invasion by deacetylating target proteins such as AKR1C1 and IDH1. In mitochondria, SIRT3, SIRT4, and SIRT5 could participate in regulating cell migration and invasion via influencing various molecular mechanisms such as integrin adhesion and EMT. Activation and inhibition effects are displayed in green and red arrows, respectively. https://biorender.com. α7nAChR alpha7 subtype of nicotinic acetylcholine receptors, ISRE IFN-stimulated response element

Migration and invasion are vital phenotypes both in physiological and pathological status. They allow normal cells to change position within tissues during embryonic morphogenesis, wound healing, and immune-cell trafficking.^358,359^ Specifically, in human cancers, they allow neoplastic cells to enter lymphatic and blood vessels for undergoing metastatic growth in distant organs.^360,361^ An increasing number of studies have shown that SIRTs play important roles in the molecular mechanisms of cell migration and invasion, such as regulation of TGF-β signaling and epithelial-to-mesenchymal transition (EMT).^362,363^ Since these two phenotypes are hallmarks during tumor progression, we introduced the potential roles of SIRT protein family in cell migration and invasion, mainly depending on cancers (Fig. 8).

##### Effect of SIRT1 on cell migration and invasion

SIRT1 deacetylates many key proteins, which also contain transcription factors, mainly involved in EMT and integrin adhesion, thereby regulating cell migration and invasion.^271,363^ EMT is the most well-established example of changes in cell–cell adhesion, which refers to nonepithelial cells that are loosely embedded in an extracellular matrix (ECM).^364^ Integrin adhesion activates pathways including TGF-β, phosphatidylinositol-4,5-bisphosphate 3-kinase (PI3K)/Akt, and AMPK signaling pathways.

Several studies have shown that SIRT1 protein levels are lower in lesion tissues than in adjacent tumor tissues or normal tissues of patients with cancer.^365–367^ This phenomenon is also observed in autoimmune disorders, which indicates that SIRT1 plays important roles in regulating cell migration and invasion.^368^ SIRT1, as a deacetylase, influences the biological functions of proteins via regulating protein deacetylation, such as deacetylation of Beclin-1. In melanoma cells, SIRT1 deacetylates Beclin-1 and then accelerates autophagic degradation of the epithelial marker E-cadherin, finally promoting EMT.^271^ Additionally, SIRT1 could regulate the expression levels of several proteins that participate in cell migration and invasion, resulting in promotion of EMT. Both in vivo and in vitro studies have shown that expression of SIRT1 in chondrosarcoma cells could effectively take part in the metastatic plasticity of the cells by inducing EMT, via enhancing expression of Twist protein, which is a critical transcriptional factor of EMT.^363^ Zinc finger E-box binding homeobox 1 is an E-cadherin‐related transcription factor. Yu et al. have reported that there is positive feedback between SIRT1 and Zinc finger E-box binding homeobox 1, which enhances EMT of osteosarcoma.^369^ SIRT1 induces deacetylation of Beclin-1 and then accelerates autophagic degradation of the epithelial marker E-cadherin, further promoting EMT in melanoma cells.^271^ Epidermal SIRT1 plays a role in wound repair. SIRT1 knockdown inhibits EMT, cell migration, and TGF-β signaling in keratinocytes.^370^ Furthermore, SIRT1 activates downstream PI3K/Akt and Notch signaling pathways, which alleviates H9c2 cell injury induced by hypoxia, via promoting cell proliferation, migration and invasion, and by inhibiting apoptosis.^371^ In non-small cell LC (NSCLC), the SIRT1-mediated AMPK/mTOR signaling pathway could promote A549 and H1299 cell proliferation, invasion and apoptosis.^372^

Expression of SIRT1 can be regulated by ncRNAs, which further influence its effects in regulating cell migration and invasion. For instance, in colorectal cancer (CRC) cells, downregulation of SIRT1, by miR-34a transfection, increases the level of acetylated-p53 and inhibits cell migration and invasion.^373^ This situation is also found in HCC.^374^ Expression of SIRT1 can also be regulated by lncRNAs or circRNAs in a ceRNA-dependent manner. For example, SIRT1 promotes cell migration and invasion in HCC. Expression of SIRT1 is upregulated by lncRNA MALAT1 via sponging miR-204, which might have a pivotal role in treatment and prognosis.^375^ Furthermore, SIRT1 promotes the migration of fibroblast-like synoviocytes in rheumatoid arthritis, which providing new insight into SIRT1 during RA progression. Mechanistically, SIRT1 is positively regulated by circ0088036 via sponging miR-140−3p.^368^

##### Effect of SIRT2 on cell migration and invasion

SIRT2 participates in regulating cell migration and invasion through deacetylating target proteins. STAT3 is an important protein for regulating cell invasion and migration.^376,377^ STAT3 has been shown to affect EMT in several cancers.^378^ Previous studies have shown that SIRT2 can deacetylate Aldo-keto reductase family 1 member C1 (AKR1C1), which is a member of the human aldo-keto reductase protein family that catalyzes NADP + -dependent reduction. AKR1C1 deacetylation further inhibits the transactivation of STAT3 target genes, thus suppressing migration in NSCLC cells and xenograft models.^379^ It has been reported that isocitrate dehydrogenase 1 (IDH1) affects cell migration in malignant tumors, such as glioblastoma.^380^ In human CRC, SIRT2-dependent IDH1 deacetylation represses CRC cell migration and invasion both in vitro and in vivo.^381^

##### Effect of SIRT3-5 on cell migration and invasion

SIRT3-5 are three main deacetylases that are located in mitochondria, which appear to be suppressors of cell migration and invasion. Previous studies have demonstrated that SIRT3 and SIRT4 negatively regulate EMT. For instance, transplantation of sh-SIRT3 cells in nude mice resulted in rapid tumor growth and larger tumors. At the molecular level, SIRT3 depletion inhibits EMT by lower E-cadherin expression, leading to tumor suppression.^382^ Sun et al. suggested that SIRT4 suppressed EMT through promoting E-cadherin expression in GC cells.^383^ Li et al. reported that SIRT3 was involved in the inhibitory effect of nicotinic alpha7 subtype of nicotinic acetylcholine receptors on platelet-derived growth factor-BB, an angiogenic factor, induced VSMC migration. Activation of alpha7 subtype of nicotinic acetylcholine receptors attenuates migration in platelet-derived growth factor-BB-treated VSMCs via a mitochondrial SIRT3-dependent manner.^384^

SIRT5 regulates cell migration and invasion in several cancer cells. For example, Dang et al. found that SIRT5 promoted migration and invasion of HCC cells.^385^ The opposite findings were reported by Yao et al. in that the inhibition of SIRT5 increased migration and invasion of HCC in hypoxic microenvironments.^386^ This inconsistent phenomenon might be attributed to the hypoxic status of tumor microenvironments. However, further studies are required for illustrating its deeper regulatory mechanisms.

##### Effect of SIRT6 and SIRT7 on cell migration and invasion

SIRT6 and SIRT7 are the least studied of the seven SIRTs to date, which are both located in the nucleus. Both of them have been found to play a role in cell migration and invasion via regulating EMT and/or MMP expression. For example, in human HCC, SIRT6 promotes N-cadherin and vimentin expression via deacetylating FoxO3a in HCC cells.^387^ SIRT6 upregulates expression of MMP9 probably through the MAPK/ERK1/2 pathway, with increased migration and invasion of OS cells.^388^ Liu et al. found that forced expression of SIRT6 attenuated EMT by suppressing the TGF-β1/ small mothers against decapentaplegic protein (Smad)3 pathway and N-terminal kinase (c-Jun) in rat models of asthma.^362^ A recent study has shown that SIRT7 promotes CRC cell invasion through the inhibition of E‐cadherin, which is the most important protein in EMT.^389^ Furthermore, SIRT7 is overexpressed in EC cells compared with normal endometrial cells. SIRT7 downregulation inhibits the invasiveness of EC cells.^390^

##### Conclusion

Taken together, the above-discussed findings suggest that SIRT proteins are involved in regulating cell migration and invasion during physiological processes and the development of human cancers. However, current research mainly focuses on the function of SIRT1 in regulating cell migration and invasion. Much work is still needed to pinpoint the precise molecular mechanisms governing the functions of other SIRTs, especially SIRT6 and SIRT7, under those conditions. It is meaningful to continue to explore the role of SIRT proteins in other diseases, which might provide future beneficial alternatives against those devastating diseases.

### Regulatory role of SIRTs in human diseases

#### SIRTs and cancer

**Fig. 9:**
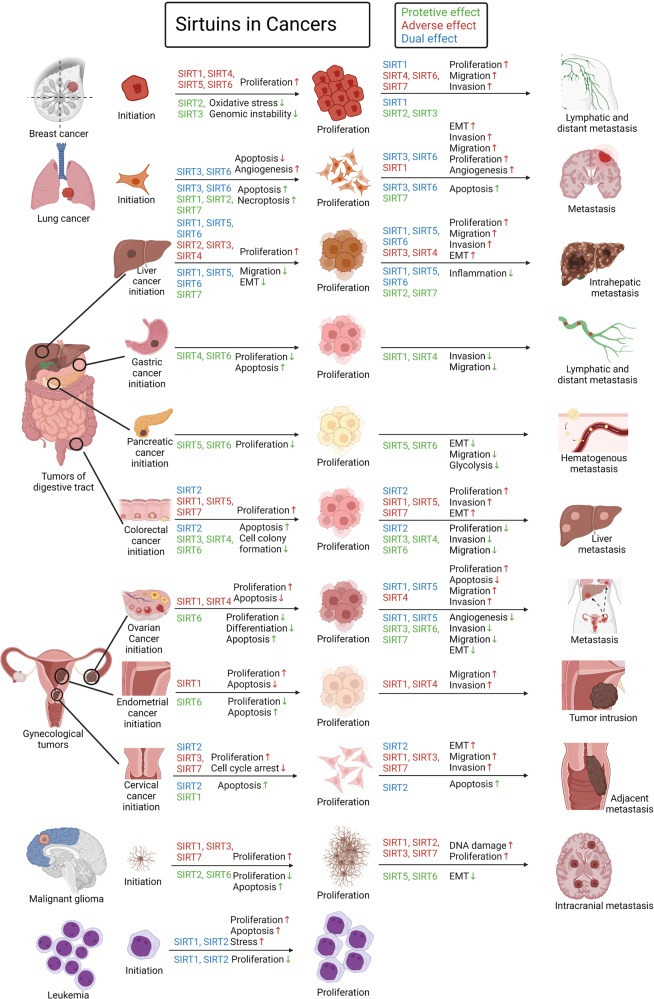
The roles of SIRTs in cancers. SIRTs are involved in a series of malignancies, including BC, LC, liver cancer, GC, PC, colorectal cancer, OC, EC, CC, malignant glioma, and leukemia. SIRTs act as tumor promoters (marked in red color), tumor suppressors (marked in green color), or both suppressor and promoter (marked in blue color). Major events in solid tumor development consist of tumor initiation, tumor proliferation, and tumor metastasis. Between these events, processes including cell proliferation, oxidative stress, apoptosis, angiogenesis, EMT, migration and invasion are promoted or inhibited. Depending on the tumor location, the metastasis site also varies, including lymph nodes, distant organs, liver, adjacent organs, etc. https://biorender.com

Cancer is currently the second most common contributor to premature mortality worldwide.^391^ Since an early diagnosis and effective treatment for patients with cancer are critical, the identification and application of effective biomarkers and novel drug targets are urgently required. Recent evidence reveals that aberrant expression of SIRTs occurs in almost all cancer types with different mechanisms, including those involved in cancer metabolism, genome stability, and the tumor microenvironment.^3^ The functions of SIRTs in the tumor process are characterized as tumor suppressor and/or oncogene, depending on genetic context and tumor type and stage.^392^ Moreover, SIRTs could exert regulatory roles in the response of the tumor to chemotherapy.^393^ These unique features suggest that SIRTs serve as potentially targetable markers and play important roles in cancer therapy. In this section, we summarize the recent studies of SIRTs in diverse cancers, which is shown in Fig. 9.

##### Breast cancer (BC)

BC is the most common malignancy throughout the world and is the fifth leading cause of cancer-related deaths.^394^ SIRT2 and SIRT4 are downregulated,^395,396^ while SIRT1 and SIRT7 are upregulated in BC tissues compared to adjacent tissues or normal tissues.^357,397^ Besides, increased SIRT2 and SIRT4 expression is correlated with longer overall survival,^395,396^ whereas increased SIRT1 and SIRT7 expression predicts a poor prognosis in patients with BC.^398,399^ These disparities might be in respect to the different roles of them in BC progression.^400^

Regarding BC development, SIRTs are generally considered as tumor suppressors but might act as tumor promoters as well. SIRT1 has been extensively explored in comparison to other SIRTs for their roles in BC, and may influence BC progression by regulating many processes, especially EMT. SIRT1 plays a critical role in regulating EMT-associated programming and thus, consequently, eliciting BC invasion and metastasis in patients with triple-negative BC.^401^ SIRT1 expression suppresses BC metastasis by reducing EMT, and invasiveness in nude mice.^402^ The effect of SIRT1 modulation on EMT in breast cancer-related cancer stem cells has also been observed. This study indicates that loss of SIRT1 destabilizes EMT inducer paired related homeobox 1, disinhibits KLF4, and activates transcription of aldehyde dehydrogenase 1, which encourages cancer stem cells, resulting in metastatic reversion.^403^ In addition to its tumor suppressive roles in BC, SIRT1 overexpression, altered EMT programming, and a decrease in tumor-suppressive miR-200a may be consistently involved in BC development and subsequent distant metastasis.^404^ The plausible explanation of the contradictory functions of SIRT1-mediated BC regulation might be due to tumor grade, tumor stage of BC, and the use of animal or human samples with a different pathological subtype.

Additionally, many other non-EMT factors that are known to function in other cellular processes in BC development could also be regulated by SIRT1. For example, the estrogen receptor (ER) and AR could mediate induction of estrogen- and androgen-responsive genes respectively and stimulate cell proliferation, and SIRT1 represses the transcriptional and proliferative response of BC cells to estrogens via an Erα-dependent mechanism.^405^ DNA polymerase delta1, the gene coding for DNA polymerase δ catalytic subunit p125, is upregulated by SIRT1, thus promoting proliferation and migration of BC cell line MCF-7.^397^ Metadherin, an oncogenic protein, has been implicated in promoting cancer progression, metastasis, and chemoresistance in BC.^406^ Activation of AMPK has been reported to reduce the expression of metadherin through enhanced SIRT1 activity along with GSK-3β in an independent manner in triple-negative BC.^406^

In addition to SIRT1, SIRT2 functions in a binary manner, as a tumor suppressor or promoter. SIRT2 acts as a tumor suppressor in BC by regulating mitosis and genome integrity. Evidence has shown that SIRT2 promotes BRCA1-BRCA1-associated RING domain protein 1 (BARD1) heterodimerization through deacetylation, thereby facilitating homologous recombination and tumor suppression.^407^ Additionally, in cancer biology, Slug, an EMT transcription factor, promotes tumor progression and metastasis.^408^ In basal-like BC, SIRT2 maintains Slug protein stability by deacetylation, which contributes to basal-like BC’s robust tumorigenic activity, along with enhanced invasive and metastatic capabilities.^409^ SIRT3, SIRT4 and SIRT7 illustrate different functions in BC progression. SIRT3 has been found to affect p53 by disruption of the ERα–p53 interaction, and decrease proliferation, colony formation, and migration in BC cells.^410^ Notably, SIRT4 could exert its tumor-suppressive activity in BC though negatively regulating SIRT1 expression via repressing glutamine metabolism, which suggests a novel crosstalk between mitochondrial and nuclear SIRT proteins in BC progression.^411^ SIRT7 depletion inhibits tumor growth via activating p38/MAPK signaling.^357^

Additionally, SIRT proteins can affect the sensitivity of BC cells to several drugs, including tamoxifen, paclitaxel and doxorubicin. For example, SIRT1 causes tamoxifen resistance in ER-α-positive BC cells through upregulation of multidrug resistance protein 2 by mediating deacetylation of FoxO1 protein.^412^ Subsequently, SIRT1 inhibition impairs nuclear FoxO1 and multidrug resistance protein 2 expression and augments the cytotoxic effect of paclitaxel and doxorubicin in tamoxifen-resistant BC cells.^412^ SIRT3 overexpression in BC cell line MTR-3 reduces the sensitivity of the resistant cells to tamoxifen.^357,413^ On the contrary, SIRT4 enhances the tamoxifen sensitivity of BC cells via inhibiting the STAT3 signaling pathway.

These findings indicate unique mechanisms of SIRT1 mediate BC regulation and its contribution to tumor development and resistance, which suggests that SIRTs are promising therapeutic targets in BC, and provides clinical strategy for overcoming drug resistance. However, the exact molecular mechanism is still uncertain and needs further investigation.

##### LC

LC is the leading cause of cancer-related deaths and the second most diagnosed cancer worldwide, with NSCLC being the most common type.^394^ Significant differences in SIRT expression between NSCLC tissues and nontumor lung tissue or adjacent tissue have been observed, which indicates that SIRTs are promising biomarkers in the diagnosis of LC.^414–416^ Notably, serum SIRT3 distinguished LC patients from healthy individuals with an area under the curve of 0.918 and optimal cutoff value of 3.12, reaching sensitivity of 86.4% and specificity of 94%.^416^ SIRTs could be potential prognostic factors for NSCLC.^414,415,417^ For example, high SIRT1-3 expression is associated with poor survival in patients with NSCLC.^414,415^

Evidence has suggested that SIRTs are key factors involved in tumor development and treatment in LC.^418,419^ Regarding LC progression, the SIRTs play conflicting roles. SIRT1 upregulated by SNHG10 suppresses NSCLC cell proliferation, as a tumor suppressor.^420^ Overexpression of SIRT1 protects NSCLC cells against osteopontin-induced NF-κB p65 acetylation and EMT, thus attenuating OPN-induced cell proliferation, migration and invasion.^421^ However, SIRT1 upregulated by circ_0001946, could promote cell growth in lung adenocarcinoma by activating the Wnt/β-catenin signaling pathway.^422^ SIRT2 and SIRT6 have been shown to exert both pro- and anticarcinogenic effects in the process of LC. For example, SIRT2 suppressed the migration of NSCLC cells by deacetylating AKR1C1, and inhibiting transactivation of STAT3 target genes.^379^ In addition, SIRT2 deacetylates the K100 residue of glycolytic enzyme phosphoglycerate mutase and facilitates its activation, resulting in enhanced NADPH production and accelerated tumor growth in NSCLC cells.^423^ Similarly, SIRT6 illustrates opposite functions in the promotion of LC development, as tumor suppressor and promoter.^424,425^ For instance, SIRT6 can coordinate with chromatin remodeler chromodomain-helicase-DNA-binding 4 to promote chromatin relaxation and DNA repair, thereby exerting an anticarcinogenic role in LC.^424^ In contrast, SIRT6 can also drive EMT and metastasis in NSCLC via snail-dependent transrepression of KLF4.^425^ This dual action of these SIRTs might depend upon the cellular context, tumor types, cancer stage, and their involvement in various cellular pathways,^392,418^ and further studies are needed to explore the exact mechanisms underlying their dual roles in LC.

Expression of SIRTs could have an influence on the chemoresistance and radioresistance of LC. SIRT1 promotes cisplatin resistance of NSCLC cells by elevating vascular endothelial growth factor A expression.^426^ SIRT1 is upregulated in cisplatin-resistant NSCLC tissues and cells compared to cisplatin-sensitive groups.^291^ SIRT1 silencing enhances the cisplatin sensitivity of H1299/cisplatin cells via suppressing autophagy. Upregulation of SIRT2 in NSCLC cells increases the sensitivity to cisplatin treatment while SIRT3 promotion reduces cisplatin resistance in LC by modulating the FoxO3/Cdc10-dependent transcript 1 protein axis.^253,427^ In relation to LC radioresistance, SIRT3 promotes DNA damage repair and radioresistance through ataxia telangiectasia mutated–Chk2 in NSCLC cells.^428^ SIRTs can affect radioresistance in LC through the regulation of tumor metabolism. Overexpression of SIRT6 inhibits key-enzyme generation in A549 cells to inhibit glycolysis and enhance radiosensitivity.^428^

In LC, SIRTs are involved in tumor development, chemoresistance and radioresistance, and exert regulatory functions by targeting different target proteins. Thus, SIRTs represent promising therapeutic targets in the perspective of precision medicine and provide new insights into therapeutic strategies for LC.

##### Gastrointestinal cancer

1. **HCC**
  HCC, the most common type of primary liver cancer with a relatively high mortality, is the sixth most common cancer and the third-leading cause of cancer-related mortality worldwide.^394^ Several bioinformatics studies have reinforced that nonmitochondrial and mitochondrial SIRTs are differentially expressed in HCC. For example, nonmitochondrial SIRT1, SIRT2 and SIRT6 are expressed at higher levels,^429–431^ while mitochondrial SIRT3-5 are expressed at lower levels in HCC tissues compared with normal liver or surrounding tumor tissue.^432–434^ SIRTs could be prognostic markers for patients with HCC. For instance, high expression of SIRT1 and SIRT7 is highly associated with poor survival, whereas low tumor levels of SIRT4 predicts a decreased survival time in HCC patients.^434–436^
  Recent studies have suggested that SIRTs could play regulatory roles in HCC development by regulating the metabolic state of the cancer cells.^437^ Referring to mitochondrial SIRTs, SIRT4 exerts a tumor-suppressive function in HCC by inhibiting glutamine metabolism.^434^ SIRT5 prevents tumor immune evasion and suppresses HCC development by orchestrating bile acid metabolism.^438^ However, SIRT5 also exerts a tumor-promoting function as a metabolic regulator. The activation of mitochondrial SIRT5 contributes to the promotion of growth and metastasis of HCC cells via glucose metabolism reprogramming from oxidative phosphorylation to glycolysis.^439^ The possible explanation for the dual role of SIRT5 in HCC could be related to its involvement in different metabolic processes, including glucose and lipid metabolism, which might result in opposite effects on tumor progression.^3^ In addition to mitochondrial SIRTs, the nonmitochondrial SIRTs can influence HCC by regulating cancer-related metabolism, especially glucose metabolism. SIRT1 and SIRT6 deacetylate hnRNP A1 to suppress glycolysis and growth in HCC.^440^ SIRT6, stabilized by ubiquitin-specific peptidase 48, attenuates HCC glycolysis and impedes metabolic reprogramming, thereby hampering HCC malignancy.^441^ SIRTs can also play roles in modulation of the cell cycle in HCC, which are essential for tumor development. Evidence has shown that SIRT4 upregulates cell-cycle governing genes p16 and p21 expression, suppresses CyclinB1/Cdc2 and Cdc25c, which normally induce cell-cycle progression, and suppresses survival to induce apoptosis in HCC cells.^442^
  Therapeutic advances targeting SIRTs are currently being explored as it is suggested that modulating SIRT3 abundance via cyclin-dependent kinase (CDK) 4/6 inhibition might enhance HCC therapy when combined with sorafenib.^443^ SIRT3 downregulates the mRNA and protein levels of glutathione S-transferase π1, a phase II detoxification enzyme involved in metabolism of chemotherapeutic agents, and SIRT3 overexpression promotes chemotherapeutic-agent-induced or sorafenib-induced apoptosis, thereby enhancing the drug sensitivity of HCC cells.^252^ Aside from mitochondrially directed deacetylase activity, SIRT6 depletion is reported to downregulate multidrug resistance protein 1 expression through the suppression of CCAAT/enhancer-binding protein, promoting enhanced HCC chemosensitivity.^444^
2. **Colorectal cancer**
  CRC ranks third in terms of cancer incidence worldwide and is the second most common cause of cancer deaths.^394^ Previous studies have shown that SIRT1 and SIRT7 are increased,^389,445^ whereas SIRT2, SIRT4 and SIRT6 are decreased in human CRC tissues compared to normal tissue, which suggests that SIRTs are potential diagnostic biomarkers for CRC.^446–448^ SIRTs are potential prognostic factors for CRC. For instance, overexpression of SIRT5 is correlated with poor prognosis in patients with CRC, while SIRT6 expression is related to improved survival.^448,449^ However, there still a need for further studies that make more clear analyses to verify the roles of SIRTs as biomarkers of CRC, such as receiver operating characteristic curve, sensitivity and specificity analyses.
  The pleiotropic roles of SIRTs in the regulation of tumor cell metabolism and cell death are strongly linked to the progression of CRC. SIRT1 has been found to affect CRC in a dose-dependent manner by regulating glutamine metabolism and apoptotic pathways. Heterozygous deletion of SIRT1 induces c-Myc expression, enhancing glutamine metabolism and subsequent proliferation, autophagy and cancer formation. In contrast, homozygous deletion of SIRT1 triggers apoptotic pathways, increases cell death, diminishes autophagy, and reduces cancer formation.^450^ The dose-dependent regulation of cellular metabolism and apoptosis by SIRT1 mechanistically contributes to the observed dual roles of SIRT1 in tumorigenesis. SIRTs have an anticarcinogenic action via modulation of CRC-related metabolism. SIRT2-dependent IDH1 deacetylation regulates cellular metabolism and inhibits liver metastasis of CRC.^381^ SIRT4 upregulates E-cadherin expression and suppresses proliferation, migration and invasion through inhibition of glutamine metabolism in CRC cells.^140^ In addition to the anticarcinogenic effects of SIRTs, SIRT5 contributes to colorectal carcinogenesis by enhancing glutaminolysis in a deglutarylation-dependent manner.^449^ SIRTs exert their regulatory function in CRC development through the modulation of several autophagy-related pathways. In particular, SIRT5 can deacetylate lactate dehydrogenase B, thus promoting hyperactivation of autophagy and tumorigenesis in CRC.^310^
  Recent evidence highlights that SIRTs are involved in various tumor processes related to chemoresistance and radioresistance in CRC. For example, overexpression of SIRT3 improves anticancer drug resistance of CRC cells through superoxide dismutase (SOD) 2 and PGC-1α regulation.^451^ In addition, SIRT4 increases the sensitivity of CRC cells to chemotherapeutic drug 5-fluorouracil by inhibiting the cell cycle.^447^ Regarding radioresistance of CRC, FoxQ1-mediated SIRT1 upregulation augments expression and nuclear translocation of β-catenin and benefits CRC-related intestinal pathological bacteria, thereby enhancing the radioresistance of CRC cells.^452^
  SIRTs play a role in regulating CRC progression, which indicates that SIRT-small-molecule-activator/inhibitor-based therapy strategies is a rescue strategy for patients with CRC. However, there have been a limited number of studies of the molecular mechanisms of SIRTs in regulating CRC progression.
3. **Gastric cancer**
  GC is the fifth most frequently diagnosed cancer with an incidence rate of 5.6%, and the fourth most common cause of cancer death with a mortality rate of 7.7% worldwide.^394^ During the past decades, SIRTs have been considered as potential druggable targets in the clinical treatment of GC. SIRT1 is upregulated in GC tissues and SIRT1 depletion promotes GC progression through activation of STAT3/MMP-13 signaling.^331^ SIRT4 and SIRT6 are downregulated in GC tissues, and their low expression is negatively correlated with tumor size and pathological grade, which predicts poor prognosis.^383,453^ Mechanistically, SIRT4 inhibits cell proliferation, migration, and invasion in GC via regulating EMT. SIRT6 inhibits the Janus kinase 2/STAT3 pathway, thereby suppressing the growth of GC. Regarding tumor resistance, SIRT6 silencing can overcome sorafenib resistance by promoting ferroptosis.^454^ Thus, SIRTs could act as novel biomarkers and therapeutic targets of GC.
4. **Pancreatic cancer (PC)**
  PC has high mortality and ranks as the seventh leading cause of cancer-related deaths worldwide.^394^ PC is also affected by SIRT activity and expression. SIRT5 expression is directly correlated with favorable prognosis, as its loss promotes glutamic-oxaloacetic transaminase 1 acetylation, thus promoting cell proliferation by enhancing glutamine and glutathione metabolism.^455^ Upregulation of SIRT6 by tumor suppressor KLF10 activity influences glycolysis, EMT, and distant metastasis of PC.^456^ SIRTs are associated with drug resistance of PC. SIRT1 can facilitate chemoresistance of PC cells by regulating adaptive response to chemotherapy-induced stress.^457^
  Collectively, SIRTs play important roles in tumor progression, chemoresistance and radioresistance in gastrointestinal cancer by regulating multiple cellular and physiological processes, including metabolism, cell cycle, cell death, and tumor microenvironment. Therefore, the potential selective modulation of SIRT protein family members represents a promising area in gastrointestinal cancer treatment. However, given the contribution of gastrointestinal cancer to worldwide morbidity and mortality, further research is needed to understand the exact mechanisms underlying the involvement of SIRTs in these cancers, especially for GC and PC.

##### Gynecological cancer

1. **Ovarian cancer (OC)**
  OC is one of the most aggressive female malignancies, with poor prognosis.^394^ SIRT1-3 and SIRT6 are significantly decreased, while SIRT5 is significantly increased in OC tissues compared to normal or adjacent tissues.^458–460^ High expression of SIRT2 and SIRT5-7 is correlated with favorable survival, while high expression of SIRT1 and SIRT4 is associated with poor survival,^458^ suggesting that SIRTs could serve as novel prognostic biomarkers. SIRTs are implicated in the development and treatment of OC. For example, SIRT1 expression suppresses high motility group box-1 protein expression and acetylation, thus inhibiting OC migration, invasion and angiogenesis.^461^ However, MHY2245, a new SIRT1 inhibitor, exert antitumor activity against OC cells by blocking the pyruvate kinase M2/mTOR pathway.^462^ In addition to SIRT1, overexpression of SIRT3 dramatically suppresses OC cell metastatic capability by inhibiting EMT via downregulation of Twist.^463^ Regarding OC treatment, cisplatin has been a pivotal drug, however, cisplatin resistance hinders the prognosis of patients.^464^ Overexpression of SIRT2 significantly enhances the sensitivity of cisplatin-resistant counterpart cells to cisplatin in OC.^465^ In addition, SIRT5 can promote cisplatin resistance in OC by suppressing DNA damage in a ROS-dependent manner via regulation of the Nrf2/Heme Oxgenase-1 pathway.^460^
2. **EC**
  EC is the most common gynecological cancer in high-income countries and its incidence is rising globally.^466^ Recent studies have shown that SIRTs participate in the development and progression of EC. For example, SIRT1 is elevated in EC cell lines and tissues and SIRT1 promotes autophagy and proliferation of EC cells by reducing acetylation of LC3.^272^ The expression of SIRT2 is increased in most human EC cell lines and SIRT2 overexpression promotes EC cell proliferation but inhibits apoptosis.^467^ In contrast to SIRT1 and SIRT2, SIRT6 might function as a tumor suppressor of EC cells. SIRT6 negatively affects the proliferation of AN3CA and KLE EC cells by repressing expression of the antiapoptotic protein surviving.^468^ Chemotherapy is crucial for postoperative adjuvant therapy of EC. SIRT1 promotes the growth and cisplatin resistance of EC cells.^469^ SIRT2 has been shown to promote cell stemness and activate the MEK/ERK signaling pathway while repressing chemosensitivity in EC.^470^
3. **Cervical cancer (CC)**
  CC is one of the most severe and prevalent female malignancies and a global health issue.^394^ Abnormal expression of SIRTs in CC tissue may be related to disease progression. For instance, the expression of SIRT2 is decreased in CC tissue compared with paired adjacent tissue, and SIRT2 expression in tumor tissue is negatively correlated with tumor size, and lymph node metastasis, which predicts favorable survival.^471^ For mechanistic studies, SIRT1 has been found to be overexpressed in HPV-infected CC cells and SIRT1 expression is correlated with poor clinical outcomes in CC.^472^ SIRT1 enables HPV-infected CC cells to continue growing by nullifying absent in melanoma 2 inflammasome-mediated immunity. Moreover, SIRT3 contributes to the reprogramming of fatty acid synthesis by upregulating acetyl-coA carboxylase 1 to promote *de novo* lipogenesis by SIRT3 deacetylation, thereby promoting the invasion and metastasis of CC cells.^473^
  SIRTs are implicated in tumor development and chemotherapy resistance in gynecological cancer including OC, EC, and CC, thus SIRTs might serve as indicators of prognosis and as promising therapeutic targets for gynecological cancer. However, evidence about the roles of SIRTs in gynecological cancer is still limited, so more studies are needed to further explore the underlying molecular mechanism by which SIRTs regulate tumor processes in these cancers.

##### Glioma

Glioma is the most common and malignant primary tumor of the central nervous system, with a poor prognosis, especially glioblastoma.^474^ SIRT1 and SIRT7 are upregulated,^475,476^ while SIRT3 and SIRT6 are downregulated in glioma tissues compared with normal or adjacent brain tissues.^477,478^ Glioma patients with higher SIRT1 or SIRT3 expression exhibit worse prognosis, whereas downregulation of SIRT5 is significantly correlated with shorter survival time in glioblastoma. These situations have suggested that SIRTs are promising prognostic biomarkers of glioma and might be involved in tumor progression.^475,477,479^

SIRT1 and SIRT6 exert a tumor suppressor effect in glioma. SIRT1-mediated p21-Activated kinase 1-deacetylation at K420 hinders autophagy and glioblastoma growth.^480^ Besides, SIRT6 suppresses glioma cell growth via induction of apoptosis, inhibition of oxidative stress, and inhibition of the activation of the Janus kinase 2/STAT3 signaling pathway.^478^ On the contrary, SIRT3 and SIRT7 are reported to play positive roles in the development of glioma. SIRT3 can stabilize Ku70–Bax interaction to enhance glioma cell viability.^477^ Moreover, SIRT7 affects the malignancy of glioma cells mainly by promoting glioma proliferation and invasion through ERK and STAT3 signaling.^476^ Evidence also suggests that SIRTs participate in the transformation of chemoresistance and radioresistance in glioma. For instance, SIRT1 inhibition increases the sensitivity of glioma cells for temozolomide via facilitation of intracellular ROS generation.^475^ In addition, CDK1-mediated SIRT3 activation could enhance mitochondrial function and contribute to adaptive radioresistance in glioma cells.^481^ Therefore, SIRTs are potential biomarkers for the prognosis and diagnosis of glioma and promising therapeutic targets.

##### Leukemia

Leukemia is a malignant clonal disease of hematopoietic stem cells, and most leukemias are sporadic and their specific etiology remains elusive.^482^ SIRTs participate in the development and therapeutic resistance of leukemia. SIRT1 promotes T-cell acute lymphoblastic leukemia progression by regulating the phosphorylation and degradation of p27 through deacetylating cyclin-dependent kinase 2.^483^ SIRT2 is overexpressed in primary acute myeloid leukemia blasts, and SIRT2 activation by nicotinamide phosphoribosyltransferase (NAMPT) reduces proliferation and induces apoptosis in human acute myeloid leukemia, possibly via the Akt/GSK-3β/β-catenin pathway.^335^ Inhibition of SIRT2 suppresses the in vitro growth and in vivo engraftment of T-cell acute lymphoblastic leukemia cells via diminished LIM domain only 2 (LMO2) deacetylation.^484^ This dual action in tumor development of SIRT2 might be due to different types of leukemia.

Regarding leukemia treatment, the combination of chemotherapeutics with SIRT modulators could provide a novel therapeutic strategy. For example, pharmacological targeting or RNAi-mediated knockdown of SIRT1 inhibits cell growth and sensitizes AML cells to tyrosine kinase inhibitor treatment.^485^ Moreover, shSIRT6-induced DNA repair deficiencies are potently synergistic with NAMPT targeting in acute myeloid leukemia treatment, which shows promising in vivo efficacy compared with monotherapy.^486^ SIRT7 expression increases with the positive response to treatment, but shows reduction when patients progress or relapse, which suggests that SIRT7 potentially serves as a general biomarker for monitoring treatment response in myeloid stem cell disorders.^487^ Accordingly, these results suggest that targeting SIRTs represents an attractive therapeutic strategy and provides a rationale for the novel combination-based treatments for leukemia.

##### Conclusion

We have reviewed the role of different SIRTs in diverse cancers, focusing on them as new anticancer therapeutic targets. Various investigations have indicated that different SIRTs show differential patterns of expression depending upon the pathological subtype, tumor grade and stage. It could be concluded that SIRTs serve as prognostic factors/biomarkers in patients with cancer. The discrepancy in the role of SIRTs in tumor progression and tumor chemoresistance or radioresistance might be attributed to various tumor types, stages, microenvironment, and their involvement in various tumor processes, such as cellular metabolism, cell death, cell cycle, and DNA damage/repair. Notably, several SIRTs exert a dual action in cancer. Thus, figuring out the underlying mechanisms and conditions that enable their opposing roles in cancer might be one of the main challenges and of great therapeutic significance. Collectively, SIRTs could be utilized as promising target molecules to be used as potential biomarkers for diagnosis and prognosis in patients with cancer. A variety of available SIRT modulators could be developed and further utilized to promote treatment efficacy of various cancers by themselves or, more likely, in combination with different anticancer drugs.

#### SIRTs and CVDs

**Fig. 10:**
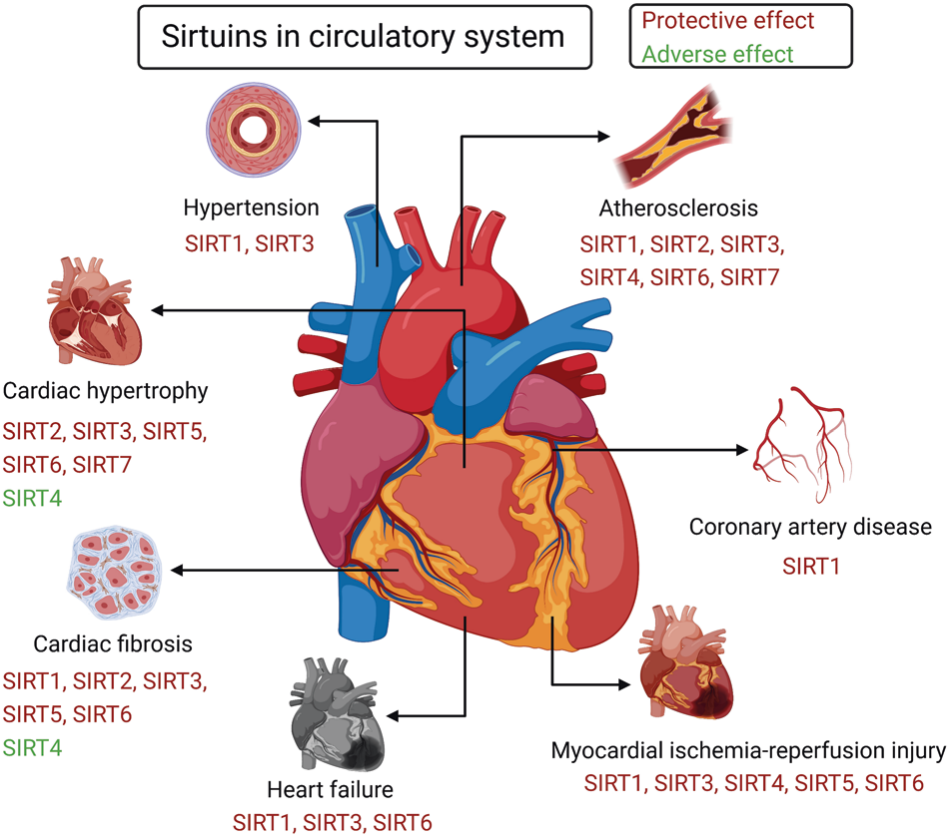
The roles of SIRTs in circulatory system. SIRT1, SIRT3 and SIRT6 play protective roles in CVDs, such as cardiac fibrosis, heart failure, atherosclerosis, and MI/R injury. In addition, the protective effect of SIRT2 is observed in cardiac hypertrophy, cardiac fibrosis, as well as atherosclerosis. Furthermore, SIRT4 has a protective effect on atherosclerosis and MI/R injury. However, SIRT4 may have an adverse effect on cardiac hypertrophy and fibrosis. In contrast, SIRT5 plays protective role in cardiac hypertrophy and fibrosis, and similar protective effect is also observed in MI/R injury. Finally, the protective effect of SIRT7 is observed in cardiac hypertrophy and atherosclerosis. https://biorender.com

Over the past decades, the incidence of CVDs, such as heart failure, atherosclerosis, and hypertension, has been increasing.^488^ CVDs are the major cause of mortality worldwide.^489,490^ According to the Global Burden of Disease Study 2019, prevalent cases of total CVDs have increased from 271 million to 523 million in 204 countries and territories between 1990 and 2019. The number of CVD deaths has also increased from 12.1 million to 18.6 million.^491^ Epigenetic modification plays a critical role in the occurrence and development of CVD^488^ and regulates the function and expression level of CVD-related genes through DNA methylation, histone modification, and non-coding RNA mechanism.^492^ Therefore, SIRT protein family has received much attention in CVD research due to its crucial role in regulating histone deacetylation.^488^ In addition to HDAC function, SIRTs also have multiple non-histone deacetylase and mono-ADP-ribosyl transferase activities.^493^ These functions also play an important role in CVDs (Fig. 10). SIRTs regulate crucial pathological processes, such as cell proliferation, cell senescence, DNA damage, oxidative stress, inflammation, and cell metabolism, thereby influencing the occurrence and development of CVDs.^493,494^

##### Cardiac hypertrophy and fibrosis

Cardiac hypertrophy is an adaptive and compensatory mechanism for maintaining cardiac output during physiological and pathological stimuli.^495^ However, some detrimental processes, such as pressure or volume overload, can lead to pathological cardiac hypertrophy.^495^ Cardiac fibrosis induces fibroblast proliferation and excessive deposition of extracellular proteins.^496^ Pathological cardiac hypertrophy and fibrosis are the main characteristics of cardiac remodeling.^497^ It is crucial to reveal the molecular mechanisms associated with cardiac hypertrophy and fibrosis, as there are currently no effective treatments for cardiac remodeling.^497^ Therefore, SIRT proteins, which have been reported to play important roles in the occurrence and development of cardiac hypertrophy and fibrosis, have received extensive attention, especially SIRT1, SIRT3, and SIRT6.

1. **Cardiac hypertrophy**
  The impact of SIRT1 on cardiac hypertrophy is inconsistent, with both alleviating and exacerbating effects having been reported.^4^ Some in vivo studies have suggested that SIRT1 overexpression can alleviate Ang II-induced cardiac hypertrophy by reducing cardiomyocyte apoptosis and promoting autophagy.^498,499^ In addition, SIRT1 overexpression can ameliorate cardiac hypertrophy induced by phenylephrine by inhibiting protein kinase C (PKC)‐ζ activation.^500^ However, some studies have shown the opposite effect. For example, SIRT1 exacerbated cardiac hypertrophy by promoting membrane localization and activation of Akt and phosphoinositide-dependent protein kinase 1, while impaired Akt activation in the hearts of SIRT1-deficient mice was related to decreased cardiac hypertrophy in response to physical exercise and Ang II.^501^ These opposite effects might be dependent on the degree of SIRT1 expression.^502^ For instance, the low (2.5-fold) or moderate (7.5-fold) overexpression of SIRT1 in the hearts of transgenic mice attenuated cardiac hypertrophy. However, a high overexpression (12.5-fold) level of SIRT1 increased cardiac hypertrophy.^502^ These conflicting effects imply that SIRT1 has different effects on cardiac hypertrophy in different contexts and models.^503^ Therefore, more studies are needed to further explore the complex effects of SIRT1 on cardiac hypertrophy.^503^
  SIRT3 has a protective role in cardiac hypertrophy.^504^ Its expression was reduced in the hearts of Ang II-induced cardiac hypertrophic mice and in Ang II-treated cardiomyocytes.^505^ In addition, SIRT3 overexpression protects myocytes from hypertrophy, whereas SIRT3 silencing exacerbates Ang II-induced cardiomyocyte hypertrophy.^505^ Resveratrol can be used to activate SIRT3 with protective effects on hypertrophy through activation of SIRT3 and subsequent autophagy.^506^ However, the protective effects of resveratrol have not been observed after the addition of siRNA-SIRT3.^506^ SIRT3 promotes autophagy in Ang II-induced myocardial hypertrophy via deacetylation of FoxO1,^507^ blocks the cardiac hypertrophic response by augmenting FoxO3a-dependent antioxidant defense mechanisms in mice,^164^ and exerts protective effects against cardiac hypertrophy by reducing the level of acetylation and activity of poly (ADP-ribose) polymerase-1.^508^
  SIRT6 also protects against cardiac hypertrophy.^509^ Both in vivo and in vitro studies have revealed that SIRT6 inhibits isoproterenol-induced cardiac hypertrophy via activation of autophagy.^314^ Specifically, SIRT6 promotes nuclear retention of FoxO3 transcription factor, possibly by attenuating Akt signaling, which is responsible for autophagy activation.^314^ In addition, SIRT6 protects cardiomyocytes from hypertrophy by decreasing the protein level of p300 and subsequently the acetylation and transcriptional activity of NF-κB p65 subunit.^510^ It also blocks IGF-Akt signaling and cardiac hypertrophy development by targeting c-Jun.^511^ Moreover, STAT3 suppression has been reported to be involved in the protective effect of SIRT6 against cardiomyocyte hypertrophy.^512^
  In addition, SIRT2, SIRT5, and SIRT7 have protective effects on cardiac hypertrophy, while SIRT4 appears to have the opposite effect. The protein level of SIRT2 is reduced in cardiac hypertrophy, and SIRT2 overexpression attenuates agonist-induced cardiac hypertrophy in a cell-autonomous manner.^513^ On a molecular level, SIRT2 binds to and deacetylates the nuclear factor of activated T-cell c2 transcription factor, thereby regulating nuclear factor of activated T-cell c2 transcription activity and exerting protective effects on cardiac hypertrophy.^513^ In contrast, loss of SIRT2 has been reported to reduce AMPK activation, thereby promoting aging-related and Ang II-induced cardiac hypertrophy and blunting metformin-mediated cardioprotective effects.^514^ These findings have suggested that SIRT2 might be a potential target for the treatment of cardiac hypertrophy. In addition, SIRT5 prevents age-related cardiac hypertrophy,^515^ while SIRT7 also ameliorates stress-induced cardiac hypertrophy by interacting with and deacetylating GATA4.^516^ Interestingly, SIRT4 seems to have an adverse effect on cardiac hypertrophy. For instance, an in vivo study has revealed that SIRT4 overexpression aggravates Ang II-induced cardiac hypertrophy by inhibiting MnSOD activity.^517^ However, further studies are needed to confirm this result.
2. **Cardiac fibrosis**
  In cardiac fibrosis, TGF-β is a key profibrotic cytokine that exerts profibrotic effects.^518^ TGF-β is involved in the protective effect of SIRT1, SIRT3, and SIRT6 on cardiac fibrosis by regulating the activity of Smad family transcription factors.^519–521^ For instance, activation of both SIRT1 and SIRT3 by resveratrol attenuates cardiac fibrosis in mice by inhibiting the TGF-β/Smad3 pathway^519,520^ and systematic SIRT6 KO induces cardiac fibrosis in mice by activating the TGF-β/Smad3 pathway.^521^ On a molecular level, the study has also shown that SIRT3 overexpression partially prevents the inflammatory and profibrotic effects by modulating the FOS/activator protein-1 pathway in human and rat cardiomyocytes.^65^ SIRT6 prevents Ang II-mediated cardiac fibrosis and injury by targeting AMPK-Angiotensin-converting enzyme 2 signaling.^522^
  In addition, other SIRTs affect cardiac fibrosis. For example, SIRT2 overexpression protects against Ang II-induced cardiac fibrosis and rescues cardiac function.^514^ This protective effect of SIRT2 is associated with the promotion of AMPK activation by deacetylating the kinase LKB1.^514^ SIRT5 KO mice have shown increased fibrosis compared to age-matched wild-type mice,^515^ although relevant mechanisms need to be further explored. However, SIRT4 appears to contribute to cardiac fibrosis, and global SIRT4 KO in mice confers resistance to Ang II infusion by significantly suppressing fibrosis deposition.^517^ Similarly, enhanced expression and phosphorylation of SIRT7 plays a role in promoting cardiac fibrosis via activation of Smad2 and ERK signaling pathways.^523^ However, SIRT7 KO in mice has been reported to result in cardiac fibrosis.^265^
  Overall, SIRTs play an important role in cardiac hypertrophy and fibrosis. SIRT1, SIRT3, and SIRT6 might protect against cardiac hypertrophy and fibrosis by affecting important biological processes and regulating downstream signaling pathways, such as autophagy and TGF-β/Smad3 pathways. Of note, SIRT1 might have bidirectional effects on cardiac hypertrophy, which might be dependent on the degree of SIRT1 expression. Furthermore, SIRT2 and SIRT5 might also have protective effects on cardiac hypertrophy and fibrosis. In contrast, SIRT4 might exacerbate cardiac hypertrophy and fibrosis. Evidence has suggested that SIRT7 has a protective effect on cardiac hypertrophy, but its effect on cardiac fibrosis is inconsistent. Considering that there are few studies on SIRT7 in cardiac hypertrophy and fibrosis, further research is needed in the future.

##### Heart failure

Heart failure is the most common endpoint of most CVDs,^524^ affecting an estimated 64.3 million people worldwide.^391,525^ It is a complex disease and involves various molecular and cellular alterations that affect the cardiac structure and impair the contractile function.^526^ However, the underlying mechanisms of heart failure remain not fully understood.^527^ Recently, growing evidence has suggested that SIRTs play key roles during the process of heart failure. The following section summarizes this evidence.

SIRT1 has beneficial effects on the development of heart failure. The expression of SIRT1 is decreased in the hearts of advanced heart failure patients and rat models.^528,529^ Heart failure is closely related to some biological processes, such as oxidative stress and cell apoptosis.^530,531^ SIRT1 might attenuate oxidative stress and protect cells from oxidative damage and apoptosis through several mechanisms.^531^ For example, levels of MnSOD, thioredoxin1, and Bcl-xL (an anti-apoptotic molecule) are significantly decreased in cardiomyocytes from individuals with advanced heart failure.^528^ The low expression of SIRT1 might downregulate antioxidants and upregulate pro-apoptotic molecules by increasing p53 acetylation and decreasing FoxO1 translocation in the nucleus.^528^ In addition, an in vivo study has suggested that SIRT1 overexpression reduces cardiomyocyte apoptosis through the NF-κB p65/miR-155/brain-derived neurotrophic factor (BDNF) signaling pathway, thereby alleviating heart failure in rats.^532^ Furthermore, reduced level and activity of sarco-endoplasmic reticulum Ca^2+^-ATPase (SERCA2a) are major features of heart failure, and SIRT1 KO elevated the acetylation of SERCA2a, which in turn leads to SERCA2a dysfunction and cardiac defects in a failing heart.^533^ In contrast, the pharmacological activation of SIRT1 restores SERCA2a activity via deacetylation at K492.^533^ Overall, the above evidence has indicated that SIRT1 is involved in the occurrence and development of heart failure and might be a promising therapeutic target for heart failure treatment.

In addition, mitochondrial energy metabolism disorder contributes to the progression of heart failure.^534^ Myocardial acetylproteomics demonstrates that there is extensive mitochondrial protein lysine hyperacetylation in mouse models of early-stage heart failure and in end-stage failing human hearts.^534^ As a mitochondrial deacetylase, SIRT3 plays an important role in maintaining the mitochondrial function,^535^ and provides a protective effect during heart failure.^536^ SIRT3 deficiency might impair cardiac mitochondrial function and aggravate heart failure during aging.^537^ In addition, SIRT3 is involved in the regulation of endothelial metabolism and angiogenesis, thereby affecting the occurrence and development of heart failure.^538,539^ For instance, an in vivo study has suggested that the endothelial‐specific SIRT3 KO disrupts glucose transport from endothelial cells to cardiomyocytes, decreases cardiomyocyte glucose utilization via apelin in a paracrine manner, and sensitizes pressure overload-induced heart failure.^538^

Similar protective effects during heart failure have also been observed in SIRT6.^511^ SIRT6 expression is significantly decreased in the hearts of patients with chronic heart failure as well as animal models of heart failure.^527^ SIRT6 overexpression increases the survival of transverse aortic constriction-induced heart failure mice, which might be associated with telomerase upregulation, such as telomerase reverse transcriptase and telomeric repeat binding factor 1.^540^

Compared to SIRT1, SIRT3, and SIRT6, studies on SIRT2,^541^ SIRT4, SIRT5,^542^ and SIRT7 in heart failure are limited. SIRTs might play important roles in the occurrence and development of heart failure and their further exploration is needed in the future. Studies on SIRT2, SIRT4, SIRT5, and SIRT7 might reveal promising research directions for the treatment of heart failure.

##### Atherosclerosis

Atherosclerosis is a chronic inflammatory disease^4^ that results from a series of events, including increased levels of LDL cholesterol in the plasma, dysfunctional endothelial cells, inflammation with immune cell infiltration, and ultimately plaque formation.^494,543^ SIRTs have been reported to directly affect atherogenesis and plaque stability by preventing endothelial cell dysfunction, VSMC senescence, and macrophage foam cell formation via regulation of key biological processes, such as DNA damage repair and anti-apoptosis and anti-inflammatory pathways.^493^

SIRT1 has a protective effect on atherosclerosis.^544^ A prior in vivo study has shown that endothelial cell-specific overexpression of SIRT1 protects against atherosclerosis in apolipoprotein E KO mice,^545^ which was associated with inhibited endothelial cell apoptosis via eNOS expression activation.^545^ In addition, SIRT1 activation by SRT1720 in aging mice ameliorates endothelial dysfunction by increasing COX-2 signaling and reducing oxidative stress and inflammation.^546^ On VSMC level, SIRT1 protects against DNA damage and inhibits atherosclerosis partly by activating the repair protein Nijmegen breakage syndrome-1.^544^ Moreover, macrophage foam cell formation is a key initiation event in the pathogenesis of atherosclerosis.^547^ SIRT1 activation reduces Lox-1-mediated foam cell formation via suppression of the NF-κB signaling pathway.^548^ In contrast, suppression of the SIRT1 signaling pathway by mTOR signaling promotes foam cell formation and inhibits foam cell egress.^549^ Several miRNAs have been revealed to have a key role in atherosclerosis by regulating the expression of SIRT1.^550,551^ For example, miR-217 downregulation might alleviate atherosclerosis via inhibition of macrophage apoptosis and inflammatory response.^550^ SIRT1 is a direct target of miR-217. SIRT1 silencing can eliminate the effects of miR-217 downregulation.^550^ The above evidence has suggested that SIRT1 is associated with the occurrence and development of atherosclerosis and might be a promising therapeutic target for atherosclerosis treatment.

Compared to SIRT1, a relatively limited number of studies have explored the roles of other SIRTs in atherosclerosis. SIRT2 decreases atherosclerotic plaque formation in LDL receptor-deficient mice by regulating macrophage polarization.^552^ SIRT3 gene expression is associated with endothelial cell apoptosis in atherosclerosis rats,^553^ and SIRT3/SOD2 signaling can be activated by circ_0,003,423, thereby protecting human umbilical vein endothelial cells from oxLDL-induced dysfunction.^554^ SIRT4 suppresses the PI3K/Akt/NF-κB signaling pathway and relieves oxLDL-induced human umbilical vein endothelial cells injury.^555^ SIRT6 protects against endothelial dysfunction, VSMC senescence, and atherosclerosis in mice.^201,556,557^ In addition, SIRT6 overexpression reduces oxLDL uptake in RAW macrophages, and SIRT6 knockdown enhances it and increases the expression of macrophage scavenger receptor 1.^558^ Finally, SIRT7 has been reported to regulate the VSMC proliferation and migration via the Wnt/β-catenin signaling pathway, which provides a promising therapeutic strategy for anti-atherosclerosis.^559^

In conclusion, the role of SIRT1 in atherosclerosis has received extensive attention. SIRT1 deficiency in endothelial cells, VSMCs, and monocytes/macrophages promotes atherosclerosis.^560^ Therefore, SIRT1 might be a potential therapeutic target for the treatment of atherosclerosis. Other SIRTs might also have protective effects on atherosclerosis. However, due to a relatively low number of studies, the relevant mechanisms need to be further explored in the future.

##### Coronary artery disease (CAD)

CAD is the result of atherosclerotic plaque development in the walls of coronary arteries.^561^ It is one of the most common causes of death in the developed countries and is responsible for about one in every five deaths.^562^ Current studies on the role of SIRTs in CAD mainly focus on SIRT1, which has a protective effect on CAD by regulating some crucial biological processes, such as oxidative stress, inflammation, cell apoptosis, and cell proliferation.

Epidemiological studies have suggested that genetic SIRT1 polymorphisms are associated with the risk of CAD,^563^ while the expression level of SIRT1 is reduced in CAD patients.^564^ SIRT1 inhibition causes oxidative stress and inflammation in CAD patients.^565^ On a molecular level, expression of downregulated SIRT1 in human CAD monocytes is related to the enhanced acetylated p53 expression levels.^565^ In contrast, SIRT1 overexpression in human CAD monocytes mitigates pro-apoptotic events and attenuates some proinflammatory events, such as upregulating expression of NF-κB and iNOS and NO concentrations.^565^ SIRT1 has been reported to be involved in the regulation of CAD via noncoding RNAs.^566,567^ For example, promoted expression of SIRT1 by elevated expression of lncRNA C2dat1 and subsequent suppressed miR-34a expression increases VSMC proliferation and migration in CAD.^567^

Except for SIRT1, epidemiological studies also suggest that genetic polymorphisms of SIRT3 and SIRT6 are associated with the risk of CAD,^563^ but the related mechanism needs to be further explored. Given the protective role of SIRT1 in CAD, other SIRTs might also be potential therapeutic targets for CAD. Therefore, exploring the roles of other SIRTs in CAD might be a promising research direction in the future.

##### Myocardial ischemia/reperfusion (MI/R) injury

In recent years, the morbidity and mortality of ischemic cardiac diseases, such as myocardial infarction, have shown an upward trend.^568^ With the development of recanalization technology, the treatment of myocardial infarction has made remarkable progress.^568^ However, MI/R injury can be induced as the treatments progress.^569^ MI/R injury is closely related to oxidative stress and apoptosis,^4^ and SIRTs play crucial roles in MI/R by controlling the above biological processes.

SIRT1 has a protective effect on MI/R injury and reduces the infarct area of the heart.^570,571^ Cardiac-specific SIRT1 KO mice have shown a significantly increased myocardial infarction area size.^572^ In contrast, cardiac-specific SIRT1 overexpression was significantly reduced in the myocardial infarction area.^572^ As for its potential mechanism, overexpression of SIRT1 leads to upregulation of antioxidant pathways mediated by FoxO1 and MnSOD and downregulation of pro-apoptotic pathways mediated by caspase-3 and Bax, thereby protecting the heart from MI/R injury.^572^ In addition, SIRT1 overexpression has been shown to be involved in ameliorating miRNA inhibition associated with MI/R injury.^573,574^ For example, upregulated SIRT1 expression resulting from miR-132 inhibition might ameliorate MI/R injury by inhibiting oxidative stress and pyroptosis through activation of PGC-1α/Nrf2 signaling.^573^ The SIRT1/AMPK/PGC-1α pathway is involved in the process by which lncRNA Oip5-as1 attenuates MI/R injury by sponging miR-29a.^574^ Like SIRT1, nuclear deacetylase SIRT6 also has a protective effect on MI/R injury. On a molecular level, SIRT6 protects against MI/R injury by increasing FoxO3α-dependent antioxidant defense mechanisms^575^ and attenuating aging-related charged multivesicular body protein 2B accumulation.^576^

In addition, the protective effects of mitochondrial SIRT3-5 have been observed in MI/R injury. An in vivo study has revealed that SIRT3 deficiency exacerbates MI/R injury.^577^ Both in vitro and in vivo models have shown that SIRT4 is downregulated in cardiomyocytes after MI/R injury, and that SIRT4 overexpression decreases myocardial infarct size.^578^ This protective effect of SIRT4 against MI/R injury has been reported to be associated with preserved mitochondrial function and reduced myocardial apoptosis.^578^ Similarly, a prior in vivo study has demonstrated that SIRT5 loss increased myocardial infarct size and MI/R injury, which might be associated with the effect of SIRT5 on modulating protein succinylation in the heart.^579^

This evidence suggests that SIRT1-6 might play critical roles in alleviating myocardial infarction and M/IR by regulating some important biological processes, such as oxidative stress and apoptosis. However, relevant molecular mechanisms behind these processes need to be further explored. Moreover, few studies have focused on the roles of SIRT2 and SIRT7 in M/IR injury, and further research is needed in the future.

##### Hypertension

Hypertension, defined as systolic blood pressure of ≥ 140 mmHg and/or diastolic blood pressure of ≥ 90 mmHg, is the risk factor for other CVDs,^580^ affecting an estimated 1.39 billion people worldwide in 2010. Its prevalence is still rising globally.^581^ In recent years, increasing studies have focused on the protective effects of SIRT1 and SIRT3 on hypertension.^582,583^ In vivo studies have shown that SIRT1 overexpression in VSMCs attenuates Ang II-induced hypertension in mice.^582^ Similarly, SIRT3 overexpression attenuates Ang II and deoxycorticosterone acetate salt-induced hypertension in transgenic mice.^583^ Both SIRT1 and SIRT3 have been reported to be involved in the regulation of oxidative stress in hypertension.^584–586^ For example, SIRT1 activation attenuates Klotho deficiency-induced arterial stiffness and hypertension by increasing AMPKα and eNOS activity.^584^ SIRT1 overexpression mediated by NAMPT alleviates Ang II-mediated ROS production.^585^ In addition, diminished SIRT3 expression and redox inactivation of SIRT3 leads to SOD2 inactivation and contributes to the pathogenesis of hypertension.^586^

SIRTs also play important roles in the complications of hypertension. For example, decreased urinary levels of SIRT1 can be seen as a non-invasive biomarker of early renal damage in hypertension.^587^ SIRT3 alleviates the development of hypertensive renal injury by suppressing EMT.^588^ Endothelial-specific deletion of SIRT6 significantly enhances blood pressure and exacerbates endothelial dysfunction and cardiorenal injury in experimental hypertension by targeting Nkx3.2-GATA5 signaling.^589^

These findings indicate that SIRTs have protective effects on the occurrence and development of hypertension and might be valuable predictive biomarkers as well as promising therapeutic targets for hypertension complications. However, relevant mechanisms still need to be further explored, especially for SIRT2, SIRT4, SIRT5, and SIRT7, which have not been extensively investigated.

##### Conclusion

This section summarized the effects of SIRTs on CVDs. The effects of SIRT1, SIRT3, and SIRT6 have received extensive attention. Most studies have shown that they have a protective effect on CVDs, such as cardiac fibrosis, heart failure, atherosclerosis, and M/IR injury. Compared to SIRT1, SIRT3, and SIRT6, studies on SIRT2, SIRT4, SIRT5, and SIRT7 are relatively limited, even though they play important roles in CVDs. The protective effects of SIRT2, SIRT5, and SIRT7 in several CVDs (e.g., hypertrophy) have been observed. Of note, SIRT4 might aggravate cardiac hypertrophy and fibrosis. Overall, SIRTs are promising therapeutic targets, and the pharmacological modulation of SIRTs can be used in the prevention and treatment of CVDs.

#### SIRTs and respiratory system diseases

**Fig. 11:**
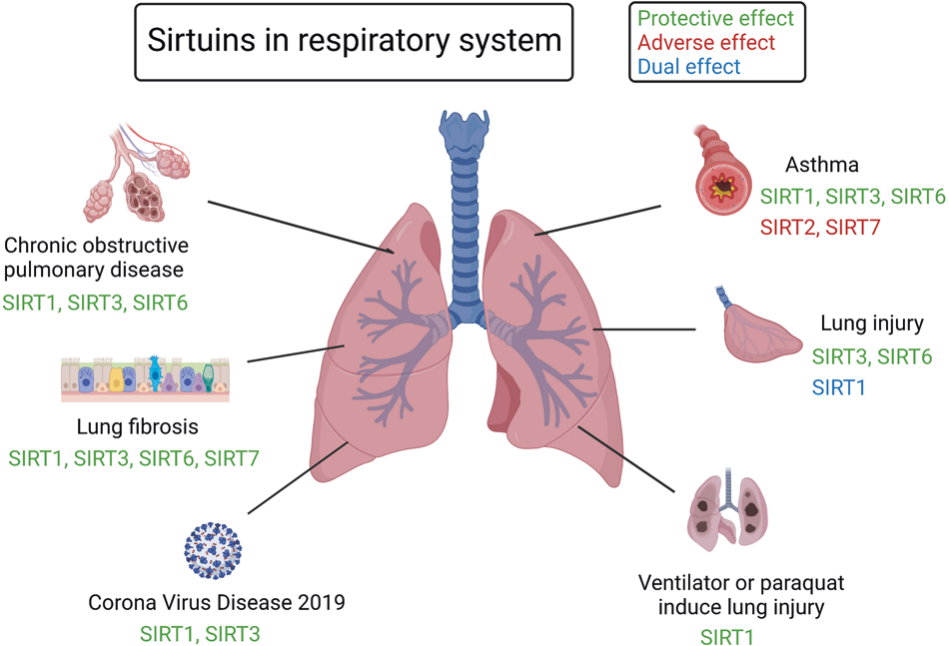
The roles of SIRTs in respiratory system. SIRTs are involved in common respiratory diseases including COPD, asthma, lung fibrosis, COVID-19, and other lung injured diseases. SIRT1, SIRT3 and SIRT6 play protective effects in COPD, and these three members also have a positive effect on asthma. However, SIRT2 and SIRT7 could aggravate the occurrence of asthma. In lung fibrosis, the positive effects of SIRT1, SIRT3, SIRT6 and SIRT7 have been demonstrated. Besides, SIRT3 and SIRT6 contribute to the remission of lung injury, whereas SIRT1 play dual effects on the disease. Moreover, the activation of SIRT1 can effectively alleviate ventilator or paraquat-induced lung injury. Finally, SIRTs are also associated with COVID-19. https://biorender.com

Respiratory diseases are one of the biggest threats to human health.^590^ Common respiratory diseases, including asthma, chronic obstructive pulmonary disease (COPD), lung fibrosis (LF), coronavirus disease 2019 (COVID-19), and other lung injures, seriously affect physical and mental health.^590^ SIRTs have received considerable attention due to their important effects on respiratory diseases.^591^ Herein, we summarize the related studies in several common respiratory system diseases (Fig. 11).

##### COPD

COPD is a common disease characterized by persistent respiratory symptoms and progressive airflow obstruction.^592^ Most chronic respiratory disease-attributable deaths are due to COPD, which is the fourth leading cause of death worldwide and considered to be a global public health challenge.^593–595^ Oxidative stress, inflammation, and apoptosis are the most important influencing factors for COPD occurrence^596^ and are closely related to SIRT family.^593^ Cigarette smoking (CS) is a causative factor for COPD. The level of SIRT1 is substantially decreased in lungs of patients with COPD/emphysema, as well as in lungs of rodents exposed to CS.^597^ Moreover, SIRT1 has been found to have anti-inflammatory, anti-apoptotic, and antioxidant roles in the pathogenesis of COPD.^598^ For example, SIRT1 plays a pivotal role in regulating NF-κB-dependent proinflammatory mediators in lungs of smokers and patients with COPD.^599^ Apart from NF-κB regulation, SIRT1 also mediates COPD via deacetylation of the FoxO3 transcription factor and tumor suppressor p53 involved in lung cell senescence and oxidative stress-induced cellular apoptosis.^597,599^ Moreover, the SIRT1 activator SRT1720 might be able to inhibit LPS-induced cytokine release from cultured peripheral blood mononuclear cells in patients with COPD. Thus, pharmacological activation of SIRT1 might have considerable potential as a novel form of chronopharmacology in COPD.^600^

SIRT6 plays an important role in the regulation of autophagy in COPD.^591^ For example, reduced SIRT6 expression level is associated with COPD development through enhancement of cellular senescence created by insufficient autophagy during CS exposure.^601^ SIRT6 overexpression weakens autophagy via IGF–Akt–mTOR signaling.^601^ Similar to SIRT1, reduced SIRT6 level is also implicated in COPD.^602^ Therefore, SIRT6 deficiency might contribute to the development of COPD.

SIRT3 is a mitochondrial deacetylase regulating mitochondrial function, and its role in the pathogenesis of COPD has also been mentioned. For instance, SIRT3 inhibits airway epithelial mitochondrial oxidative stress, thereby contributing to attenuating the progression of COPD.^603^ Therefore, activating the SIRT3 signaling pathway might present a novel therapeutic target to slow or prevent the pathogenesis of COPD.

With the understanding of the positive roles of SIRT1, SIRT3, and SIRT6 in COPD, their pharmacological activation by specific agents might be a promising strategy against COPD. However, other SIRT family members have not yet been studied in the respiratory system. SIRTs mainly mediate this disease via inflammation- or autophagy-related pathways.^604,605^ In addition, COPD is commonly thought to be associated with other chronic diseases, especially those where accelerated aging is involved. Therefore, the defection of anti-aging molecules, such as SIRTs, has been proposed as a mechanism for accelerated lung aging in COPD.^606^ Given the severity and complexity of COPD, further studies are necessary to validate the exact roles of SIRTs.

##### LF

LF is a leading cause of death in the industrialized world, which significantly increases with age.^607^ An epidemiological study has shown that approximately 45% of global deaths have been attributed to fibrosis.^607^ The pathogenesis of LF is complex and involves environmental influences and microorganisms.^608^ Recent developments in the field of LF have pointed towards the pivotal role of SIRTs in regulating disease progression, thereby qualifying as potential anti-fibrotic drug targets.^607^ Four of the seven SIRTs (SIRT1, SIRT3, SIRT6, and SIRT7) have been investigated in LF, while the functional roles of the remaining SIRTs (SIRT2, SIRT4, and SIRT5) remain elusive.

SIRT1 loss might be involved in the pathogenesis of LF. Thus, its activation might be an effective treatment for LF. SIRT1 plays an important role in regulating alveolar epithelial cell 2 progenitor renewal and LF.^609^ Mechanistically, SIRT1 activation promotes self-renewal and differentiation of alveolar epithelial cell 2 in lung tissues of idiopathic pulmonary fibrosis (IPF) patients and aged mice.^609^ However, the opposite results have been reported for SIRT1 changes in LF. According to the study performed by Zeng et al., SIRT1 expression was significantly increased in lungs from patients with IPF, as well as in lungs from bleomycin-induced LF mouse models.^610^ Nevertheless, SIRT1 activation or overexpression attenuates LF through regulation of canonical TGF-β1/p300 signaling. In addition, SIRT1 activation has been used in aging-related LF prevention and therapy.^611^ As the expression of SIRT1 in LF is controversial, more studies are needed to explore this notion in the future.

Due to the preferential mitochondrial association with extended life span in humans, SIRT3 is a protein of particular interest in age-related diseases, including LF.^612^ For example, there is a SIRT3 deficiency within the murine aging lung, which promotes the fibrotic response mediated by TGF-β1.^612^ TGF-β1 is a major multifunctional cytokine that is known as a mediator implicated in LF pathogenesis.^613^ In addition, SIRT3 deficiency promotes LF by augmenting alveolar epithelial cell mitochondrial DNA damage and apoptosis.^614^ Cheresh et al. have suggested that SIRT3 overexpression can ameliorate asbestos-induced pulmonary fibrosis.^615^ Thus, improvement in SIRT3 expression might be a novel therapeutic focus for managing patients with IF.

It is possible that SIRT6 participates in the inhibition of cellular fibrosis by regulating the TGF-β1 signaling pathway. SIRT6 can also be an ambitious target molecule for understanding the pathogenesis of IPF through the inhibitory role in TGF-β-induced cellular senescence.^616^ Additionally, Chen et al. have shown that targeting SIRT6 is a potential novel therapeutic strategy for pulmonary fibrosis that involves inactivating the TGF-β1/Smad2 signaling pathway.^617^ Furthermore, SIRT6 prevents TGF-β1-induced lung myofibroblast differentiation by inhibiting the TGF-β1/Smad2 and NF-κB signaling pathways.^618^ SIRT6 also inhibits EMT during IPF by inactivating TGF-β1/Smad3 signaling,^619^ highlighting the critical role of SIRT6 in LF. Moreover, all SIRTs show a tendency to be expressed at lower levels in fibroblasts from patients compared to controls, but the greatest decrease is observed with SIRT7.^620^ Furthermore, the decline in SIRT7 in LF has a profibrotic effect, which is mediated by changes in Smad3 levels.^620^

The above evidence shows that SIRT1, SIRT3, SIRT6, and SIRT7 are beneficial for preventing and improving the pathogenesis of LF. However, the modulatory roles of other SIRT members remain unclear. Mazumder et al. have reviewed the regulatory roles of under-reported SIRTs (mainly SIRT2, SIRT4, and SIRT5, which lack direct reported associations with LF) in basic cellular and mitochondrial metabolic pathways critical to LF.^607^ Overall, they have suggested that SIRTs appear to exert a protective action in LF, except SIRT2, which might have a pro-fibrotic action given its proinflammatory effects observed in asthma.^607^ In summary, studies on the function of SIRTs in regulating LF have potential.

##### Asthma

Asthma is a chronic inflammatory disease that is characterized by cough, breathlessness, and episodic wheezing caused by airway inflammation and hyperresponsiveness.^621^ It is estimated to influence about 300 million people all over the world, with a significantly increasing prevalence.^621,622^ Asthma affects all age groups, but particularly children^623^ SIRT1-targeting approach has been shown to be a potentially effective new strategy for the treatment of asthma.^621,624^ For example, SIRT1 protein levels are decreased in patients with severe asthma.^625^ SIRT1 exerts an anti-inflammatory effect on airway diseases, including asthma. Tang et al. have investigated the potential role of SIRT1 in regulating inflammation through modulation of IL-6 expression in an Akt-dependent manner during allergic asthma.^626^ Similarly, SIRT1 regulates IL-6 level via the Akt pathway, thereby affecting pulmonary function in asthma patients.^627^ In addition, SIRT1 inhibits the differentiation of IL-9-producing CD4^+^ T cells that are associated with allergic airway inflammation via mTOR-HIF-1α-dependent signaling coupled with glycolytic pathway.^628^ In addition, a study performed by Liu et al. has shown that anthocyanin inhibits airway inflammation by blocking the NF-κB pathway via the miR-138-5p/SIRT1 axis in asthmatic mice.^621^ All of these studies have demonstrated that SIRT1 suppresses the allergic airway inflammation that occurs in asthma and suggested that SIRT1 activation might represent a therapeutic strategy for asthma.

In addition to SIRT1, SIRT2, SIRT3, SIRT6, and SIRT7 have also been reported to be involved in asthma. For example, SIRT2 enhances allergic asthmatic inflammation, while pharmacologic SIRT2 ablation attenuates and genetic SIRT2 overexpression exaggerates the allergic asthmatic phenotype.^629^ Moreover, SIRT2 aggravates asthmatic inflammation by upregulating T-helper type 2 responses and macrophage polarization.^630^ In contrast, upregulation of SIRT3 expression reduces apoptosis in the bronchial epithelium and airway inflammation in asthma.^631^ Allergic asthma is a chronic inflammatory airway disease involving airway remodeling that severely limits airflow in the lungs.^632^ SIRT6 and SIRT7 expression levels have been found to be increased in human bronchial epithelial cells isolated from patients with asthma.^633^ Upregulated SIRT6 ameliorates airway remodeling through regulation of EMT in asthma.^634^ In contrast, upregulated SIRT7 promotes airway remodeling in asthma by regulating TGF-β1-induced airway smooth muscle cell proliferation and migration,^635^ indicating a different SIRT6 role during airway remodeling.

Asthma is a complex respiratory disease with an increasing incidence worldwide. Individuals with asthma need to receive emergency treatment if their symptoms become severe. However, there is currently no effective cure. Therefore, the pathological mechanism of asthma needs to be investigated further. Accumulating evidence shows that asthma is caused by chronic inflammation and that SIRTs have important effects on regulating chronic inflammatory responses. Future exploration of the molecular mechanisms of SIRT-mediated inflammatory response will provide more information for the development of novel therapeutic targets in asthma.

##### Lung injury

Acute lung injury (ALI) is a potentially life threatening and devastating disease with an extremely high rate of mortality.^636^ It is a clinical syndrome associated with respiratory dysfunction and is often a complication of sepsis.^637^ Additionally, ALI can develop into acute respiratory distress syndrome in more serious injuries, which lacks novel and efficient therapies.^638,639^ Inflammation and oxidative stresses are essential for the progression of ALI.^640^ However, the molecular mechanisms of sepsis-induced lung inflammatory injury are yet to be determined. SIRT1 has been widely reported to exert its anti-inflammatory function by regulating the production of proinflammatory cytokines.^637,641^ For instance, overall SIRT1 KO mice are highly susceptible to sepsis-induced inflammatory lung injury due to activation of proinflammatory transcription factor NF-κB.^641^ In addition, resveratrol is a potent SIRT1 activator that reduces ALI in an LPS-induced sepsis mouse model via activation of SIRT1.^637^ On the contrary, SIRT1 inhibitor EX-527 suppresses mTOR activation and alleviates ALI in mice with endotoxemia.^642^ This finding suggests that SIRT1 might be a detrimental factor under certain pathological conditions.

Evidence also shows that SIRT3 and SIRT6 have positive effects on ALI. SIRT3 promotes the expression of MnSOD, and this regulation is crucial for the protective effect of SIRT3 on hyperoxia-induced ALI.^643^ SIRT3 can also diminish inflammation and mitigate endotoxin-induced ALI.^63^ Kurundkar et al. have shown that SIRT3-deficient mice (SIRT3^−/−^) develop more severe ALI compared to wild-type controls (SIRT3^+/+^). Macrophages obtained from SIRT3^−/−^ mice show significant alterations in mitochondrial bioenergetic and redox homeostasis in association with proinflammatory phenotype characterized by NLRP3 inflammasome activation.^63^ Similarly, SIRT6 regulates macrophage polarization to alleviate sepsis-induced acute respiratory distress syndrome via dual mechanisms both dependent on and independent of autophagy.^644^

Apart from the above-mentioned LPS and endotoxin, other external factors, such as ventilator and paraquat, also cause lung damage.^645,646^ Mechanical ventilation contributes to excessive mechanical stress and impaired physiological and structural lung integrity. HDAC inhibited by SIRT1-silencing RNA attenuates NAMPT expression in ventilator-induced lung injury.^645^ Paraquat, which is a highly toxic herbicide and primary lung attacker, results in severe ALI.^647^ A recent study has demonstrated that resveratrol reduces paraquat-induced lung injury by upregulating SIRT1 mRNA and protein expression in combination with the Nrf2 antioxidant pathway.^646^ Therefore, SIRT1 activation can effectively alleviate lung injury.

These results suggest that SIRT protein family plays an important role in maintaining normal homeostasis and protective mechanisms in the lung. SIRTs regulate lung injury by inhibiting the expression of inflammatory factors, although specific mechanisms require further investigation.

##### Coronavirus disease 2019

In December of 2019, a new strain of coronavirus, severe acute respiratory syndrome–coronavirus 2, was first identified and called COVID-19.^648^ The disease has been recognized as pandemic by the World Health Organization. A dysregulated inflammatory profile plays an important role in COVID-19 pathogenesis.^649^ It has been reported that the SIRT family has a part in this mechanism. The unbalanced p53/SIRT1 axis might impact lymphocyte homeostasis in COVID-19 patients.^649^ COVID-19 can be characterized not only by an increase in p53 transcription in circulating lymphocytes, but also by a persistently activated p53 form, possibly due to the low level of SIRT1.^649^ Therefore, increased SIRT1 expression might help to alleviate the pathogenesis of COVID-19. Additionally, serum SIRT3 levels are associated with the clinical outcome and prognosis of COVID-19 patients.^650^ SIRT3 levels are markedly lower in severe patients compared to those in the mild/moderate patients, indicating a positive role of SIRT3 in alleviating COVID-19.

In conclusion, SIRTs are involved in COVID-19 and may provide a new therapeutic strategy. However, the impact of SIRTs on regulation of inflammatory homeostasis in severe and mild cases of COVID-19 remains to be determined.

##### Conclusion

This section discusses how the SIRT family plays a vital role in various molecular pathways in the respiratory system. SIRTs might be targets for respiratory system-related adverse health events. Increased activity of individual SIRTs often has beneficial effects in pathophysiological conditions, whereas reduced activity is usually associated with disease conditions.^651^ This also seems to apply to the respiratory system diseases. In addition, epigenetic alteration is implied in the occurrence and development of various respiratory diseases.^652^ Since SIRTs are NAD-dependent deacetylases, their deacetylation activity via epigenetics might be a new research strategy. However, detailed epigenetic roles of SIRTs in the respiratory system still need to be further explored.

#### SIRTs and digestive system diseases

**Fig. 12:**
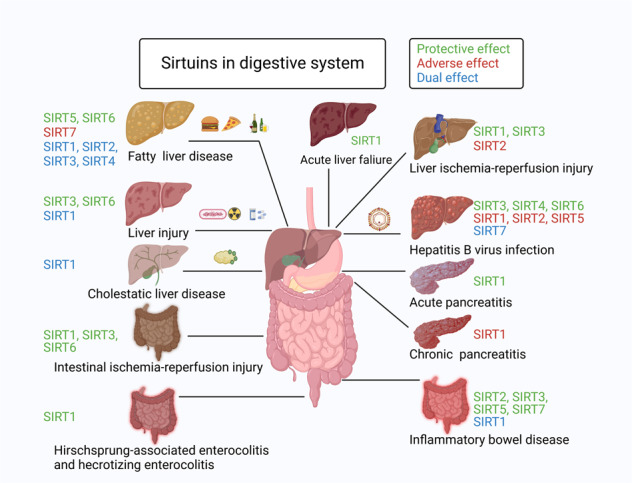
The roles of SIRTs in digestive system, mainly including FLDs, liver and intestinal ischemia-reperfusion injury, HBV infection, pancreas diseases, and IBDs. In FLDs, SIRT1, SIRT2, SIRT3, and SIRT4 could provide protective effects, while the role of SIRT7 may be harmful. Notably, the effects of SIRT1, SIRT2, SIRT3, and SIRT4 may be dual effects; in different causes of liver injury, SIRT3 and SIRT6 are beneficial, while SIRT1 plays dual roles; in HBV infection, SIRT3, SIRT4, and SIRT6 can block viral replication, SIRT1, SIRT2, and SIRT5 may contribute to the HBV-induced pathomechanism in nontransformed hepatocytes, while the effect of SIRT7 could be dual; in liver ischemia-reperfusion injury and intestinal ischemia-reperfusion injury, SIRT1 and SIRT3 could reduce tissue damage, and SIRT6 could also protect intestinal ischemia/reperfusion injury, while SIRT2 augments liver ischemia-reperfusion injury; SIRT2, SIRT3, SIRT5, and SIRT7 have a protective role in inflammatory bowel diseases, however, SIRT1 may play opposite role; in other digestive diseases, SIRT1 acts inconsistently. https://biorender.com

Digestive system diseases, including fatty liver diseases (FLDs), liver and intestinal ischemia-reperfusion injury (IRI), hepatitis B virus (HBV), pancreas diseases, and inflammatory bowel diseases (IBDs), are the most common clinical diseases.^653,654^ Increasing evidence has suggested that changes in SIRT activity and expression are associated with etiology of various digestive diseases.^280,655^ As discussed below, SIRTs play an important role in maintaining the homeostasis of digestive system function and participating in the occurrence and development of digestive diseases (Fig. 12).

##### Liver diseases

1. **FLDs**
  The disease spectrum of FLDs, with high-fat/high-calorie diets, heavy alcohol consumption, and/or other causes of metabolic disorders, ranges from simple steatosis to steatohepatitis, fibrosis, and, ultimately, cirrhosis and carcinoma.^656,657^ Notably, nonalcoholic fatty liver disease (NAFLD) is the most common liver disease, with a worldwide prevalence of 25%. About 2 billion people consume alcohol worldwide and upwards of 75 million are at risk of alcohol-associated liver diseases.^658,659^ The accumulated evidence has shown that SIRTs have complex effects on the FLD. The dual effects of SIRT1-4 have been explored, while SIRT6 might only have a protective role. However, limited studies have investigated the roles of SIRT5 and SIRT7.
  SIRTs might play a protective role in FLD, except for SIRT7. Decreased expression of SIRT1-3, SIRT5, and SIRT6 in patients with NAFLD or fibrosis have been observed in several studies.^660–662^ Molecular mechanism studies have also demonstrated this protective effect. For example, SIRT1 and SIRT6 deacetylate the carbohydrate response element-binding protein and sterol regulatory element-binding protein-1c, which are two major transcription factors responsible for the coordinated induction of glycolytic and lipogenic genes, thereby maintaining lipid homeostasis in the liver.^5,136,663,664^ In addition, SIRT1 and SIRT6 antagonize liver fibrosis by blocking the activation of hepatic stellate cells via the deacetylation function in a mouse model.^655,665^ Notably, SIRT1 might act as a key metabolic/energy sensor, which directly regulates transcriptional activity and/or gene expression of several crucial transcription factors and transcription co-activators that are involved in lipid metabolic homeostasis to play a protective role in FLD.^666–668^ These include PPAR-α, PPAR-γ co-activator 1 alpha, and NF-κB.^666–668^ Moreover, liver fibrosis, oxidative stress, and related gene expression are significantly elevated in hepatocyte-specific SIRT6-KO mice with nonalcoholic steatohepatitis.^158,669^
  The beneficial effects of SIRT2-5 on FLD might also be mediated by various biological mechanisms. Although related studies on SIRT2 are limited, it has been demonstrated that SIRT2 prevented NAFLD by deacetylation of hepatocyte nuclear factor 4α, a master regulator of gene expression for bile acid, lipids, and glucose metabolism.^670^ SIRT3 and SIRT5 improve mitochondrial function and increase mitochondrial fatty acid oxidation to relieve hepatic steatosis.^671,672^ For example, hepatic overexpression of SIRT3 improves mitochondrial function by deacetylation of mitochondrial trifunctional proteins and long-chain acyl-coenzyme A dehydrogenase.^671,672^ In systematic SIRT5 KO mice, impaired mitochondrial medium-chain fatty acid oxidation drove periportal macrovascular steatosis.^673^ Results from in vitro models have shown that SIRT4 upregulation might inhibit high fat diet-induced lipid accumulation, inflammation, and fibrogenesis through the SIRT4/Smad4 axis. It can also inhibit hepatic stellate cell activation.^662,674^
  Although SIRTs are involved in FLD as protective factors in most studies, the high expression of SIRT1, SIRT2, SIRT4, and SIRT7 in patients with alcoholic hepatitis or NAFLD and upregulation of SIRT3 after chronic alcohol exposure in mouse liver might highlight their harmful effects.^224,661,675,676^ Results from in vitro studies have suggested that elevated monocyte SIRT1 and SIRT7 levels can prevent p-FoxO3 formation and cause a defect in apoptosis in alcoholic hepatitis.^224^ Although hepatocyte apoptosis is related to disease severity, proinflammatory hepatic macrophages also undergo apoptosis in response to alcohol. Therefore, apoptosis serves as a mechanism that suppresses the inflammatory response in alcoholic liver disease.^677^ These results suggest that high SIRT1 and SIRT7 levels in myeloid cells could be a primary event leading to enhanced inflammation, possibly owing to the deleterious consequence of apoptosis.^677^ Intestinal SIRT1 also exerted a partially harmful effect on alcoholic liver disease by intensifying hepatic ferroptosis and inflammation due to the imbalance of gut microbiota.^678^ Thus, it is logical to speculate that intestinal SIRT1 might act as a proinflammatory factor. SIRT2 appears to have a deleterious effect on hepatic fibrosis via the SIRT2/ERK/c-Myc axis.^675^ Moreover, in both NAFLD and alcoholic fatty liver disease mouse models, liver-specific SIRT3 knockdown alleviated alcoholic feeding-induced liver injury and lipid accumulation, which was associated with improved autophagy induction.^676,679^ In addition, SIRT4 might have harmful effects on NAFLD, and its molecular mechanism may be partly associated with deacetylating and destabilizing mitochondrial trifunctional protein-α.^661^
  Overall, the above findings demonstrate the complex role of SIRTs in FLD. Choosing to develop different SIRT agonists or inhibitors might be a new target for the control of FLD occurrence and development in a clinical setting.
2. **Liver IRI**
  IRI is a major complication of hemorrhagic shock, resection, and transplantation. It is characterized by aseptic inflammation and liver cell death and acts as a risk factor involved in acute and chronic rejection.^680^ Current studies have mostly focused on the effect of SIRT1-3, which plays different roles via multiple molecular pathways in liver IRI.
  SIRT1 and SIRT3 might have a beneficial effect against damage. SIRT1 was markedly decreased after IRI in human and mouse livers.^280^ High SIRT1 levels improved hepatocellular function and resulted in superior survival in human liver transplants.^681^ The results from in vivo studies showed that SIRT1 suppressed mitochondrial dysfunction of ischemic mouse livers in a mitofusin 2-dependent manner.^280^ Moreover, overexpression of SIRT1 alleviated autophagy depletion and inflammation to partially mitigate hepatocellular injury during IRI.^281,682,683^ In addition, SIRT3 expression was suppressed in systematic KO of Takeda G protein-coupled receptor 5 in mice, thus leading to a proinflammatory response in macrophages, which significantly exacerbated liver injury and inflammatory response.^684^
  Unlike SIRT1 and SIRT3, with an increasing expression in IRI liver tissues, SIRT2 potentially has a detrimental effect on liver IRI.^685^ SIRT2 deacetylates MAPK phosphatase‐1 and activates the MAPK signaling pathways during liver IRI, thereby augmenting inflammatory responses and enhancing cell death in a mouse model. Pharmacologic and genetic suppression of SIRT2 also provided additional evidence supporting this observation.^685^
  Overall, SIRT1 and SIRT3 have protective effects on liver IRI, while SIRT2 might be harmful. Studies on the role of SIRT4-7 in this disease are lacking. Future research should clarify the role of SIRTs in liver IRI, including the exact molecular mechanisms.
3. **HBV infection**
  HBV infection affects over 250 million chronic carriers, causing more than 800,000 deaths annually, although a safe and effective vaccine is available.^686^ Notably, current evidence shows that the expression of SIRTs might make a difference during HBV infection.
  SIRT3, SIRT4, and SIRT6 are downregulated in patients who tested positive for HBV antigens or the cell for HBV replication.^442,687,688^ SIRT3 and SIRT6 inhibit HBV replication via epigenetic regulation.^687,688^ SIRT3 and SIRT6 induce a decrease in H3K9 acetylation on viral covalently closed circular DNA (cccDNA), serving as a template for all viral transcripts.^687,688^ Specifically, stable HBV X protein transfection suppresses SIRT4 expression, which demonstrates the interaction between HBV and SIRT4 in the context of HCC.^442^
  However, slightly elevated mRNA levels of SIRT1, SIRT2, SIRT5, and SIRT7 in HBV-infected hepatocytes lead to global histone hypoacetylation signatures, which contribute to HBV-induced pathomechanism in non-transformed hepatocytes.^689^ Pharmaceutical agonists of SIRT1, such as resveratrol, activated HBV transcription, while small-molecule inhibitors of SIRT1, including sirtinol and Ex527, exhibited anti-HBV activity, showing that SIRTs might be an anti-HBV target.^690^ SIRT7 also has a protective function in cccDNA via desuccinylation. SIRT7 restricts HBV transcription and replication by catalyzing desuccinylation of histone H3 associated with cccDNA minichromosome.^691^
  The exact molecular mechanisms underlying the alteration in SIRT expression are still not fully elucidated due to limited research, especially the conflicting roles of SIRT7 in HBV infection. Therefore, it is crucial to further examine the functions and molecular mechanisms of SIRTs in regulating the development of HBV infection and HBV-induced diseases.
4. **Other liver diseases**
  SIRT1, SIRT3, and SIRT6 might have effects on other liver diseases, including acute liver failure, hepatitis C virus (HCV) infection, autoimmune liver diseases, cholestasis diseases, and liver injury induced by a variety of causes.^692–694^
  In the acute liver failure induced by D-galactosamine/LPS, a dramatic decrease in SIRT1 levels has been documented in a rat model.^695^ SIRT1 might have a protective effect by inducing HIF-1α deacetylation to reduce the ROS levels in mice.^692^ The treatment by SITR1-activating compounds, including quercetin (natural polyphenol) and SRT1720 (synthetic SIRT1 activator), might also support the beneficial role of SIRT1 in acute liver failure.^696^
  SIRT1 plays an important role in the process of HCV infection. For example, the related study showed that elevated SIRT1 at protein level had an anti-aging effect on senescent CD4^+^ T cells during HCV infection.^697^ In addition, HCV core protein could induce dysfunction of liver sinusoidal endothelial cell by down-regulation of SIRT1.^698^ Interesting, an in vivo study showed that HCV core protein 1b-induced hepatic steatosis could be alleviated in liver-specific SIRT1 KO mice by downregulation of PPAR-γ2 expression.^699^ Therefore, the role of SIRT1 in the process of HCV infection should be further studied.
  Additionally, SIRT1 may have beneficial effects on autoimmune hepatitis. For instance, an in vivo study showed that SIRT1-null mice developed an autoimmune-like disease related with the accumulation of immune complexes in the liver.^700^ Meanwhile, evidence also suggested that the activation of SIRT1 by resveratrol could protected against concanavalin A-induced autoimmune hepatitis in aged mice by repressing the expression of p66^shc^.^701^ Interestingly, pregnancy induces a state of immune tolerance, which can lead to spontaneous improvement of clinical symptoms of autoimmune hepatitis.^702^ As for mechanism, this may be associated with the activation of SIRT1 by chorionic gonadotropin signaling.^702^
  Moreover, SIRT1, SIRT3, and SIRT6 may play important roles in cirrhosis.^665,669,703^ The study suggested that SIRT1 and SIRT6 were decreased at protein level in the livers of patients with cirrhosis.^665,669^ Besides, an in vivo study also suggested that enhanced expression of SIRT3 by curcumin had protective effects on cirrhosis.^703^ However, the exact molecular mechanism regarding cirrhosis should be further explored.
  SIRT1 and SIRT6 show protective effects in drug-induced liver injury. Suppressing SIRT1 by miR-128-3p aggravated doxorubicin-induced liver injury by promoting oxidative stress.^693^ Upregulated SIRT1 pathway by quercetin attenuated NLRP3 inflammasome activation and apoptosis to protect isoniazid-induced liver injury, while SIRT1 inhibitor EX527 reversed the protective effect.^704^ Moreover, overexpression or pharmacological SIRT6 activation enhanced glutathione and decreased N-acetyl-p-benzo-quinoneimine, thus alleviating acetaminophen-induced hepatotoxicity via normalization of liver damage, inflammatory infiltration, and oxidative stress in a mouse model.^705^
  Interestingly, hepatocyte SIRT1 might be a detrimental rather than protective factor in the setting of endotoxemic liver injury. Mechanistically, SIRT1-deacetylated p65 and compromised NF-κB activity in hepatocytes leads to increased susceptibility to endotoxemic injury when confronted with LPS/TNF-α stimulation.^706^ However, the evidence points to a dual role by which SIRT1 overexpression might contribute to cholestasis disease progression. Based on a mouse model of cholestatic liver disease, intestine-specific deletion of SIRT1 impaired systemic bile acid homeostasis.^707,708^ In an in vivo model of cholestatic disease, SIRT1-overexpressing myeloid cells with macrophage activation contributed to liver injury and fibrosis by activating the inflammasome and attenuating autophagy.^709^ Therefore, the role of SIRTs might be varied in different liver diseases. In addition, a loss or decrease in levels of SIRT3 could be an underlying factor and contributor to a damage-permissive phenotype in radiation-induced long-term persistent liver injury.^694^
  Collectively, SIRTs play complex roles in liver disease. SIRTs might act as a double-edged sword and might be related to a specific disease mechanism and cellular type of action. More studies are needed to explore the role of proinflammatory SIRT effects in liver disease.

##### Pancreatic diseases

The incidence of acute pancreatitis (AP) has increased globally to approximately 34 cases per 100,000 persons annually with an increased risk of death.^710^ Currently, few studies have explored the effects of SIRTs on AP. SIRT1 has been reported to have a low expression in a rat model and has shown a protective effect.^711^ Resveratrol protects against acute necrotizing pancreatitis in mice by enhancing SIRT1-mediated deacetylation of p53 and heat shock factor 1.^711,712^

However, SIRT1 has an opposing effect on chronic pancreatitis compared to AP. A related study has shown that SIRT1 was significantly upregulated in chronic pancreatitis and PC and in the absence of SIRT1 expression inhibition by miR-278 contributed to inflammation-induced EMT.^713^ The conflicting roles of SIRT1 in these studies imply a potentially different effect of SIRT1 on pancreatitis. Overall, further studies are needed to verify the exact role of SIRTs in this disease.

##### Intestinal diseases

1. **IBDs**
  IBDs are lifelong and incurable chronic inflammatory diseases affecting 6.8 million people worldwide.^714^ Most SIRTs can alleviate IBD.
  SIRT2 and SIRT6 are downregulated in IBD patients and their deletion promotes inflammatory responses by regulating the NF-κB pathway activation, which highlights their protective roles.^715–717^ In addition, SIRT2 inhibits Wnt/β-catenin signaling to maintain gut homeostasis.^333^ Evidence also supports the role of SIRT6 in the resistance of intestinal epithelium to injury, at least in part by preserving the expression of Rspo1, a critical growth factor in intestinal epithelial cells.^715^ Limited research has shown that systematic SIRT3, SIRT5, and SIRT7 KO mice were susceptible to colitis.^718–720^
  However, the dual role of SIRT1 in IBD has been observed in several studies. There is a significant downregulation in mRNA and protein expression of SIRT1 in patients suffering from IBD.^721^ A decrease in SIRT1 with an increase in age has been shown to aggravate colitis and cause other impairments in a mouse model.^722^ SIRT1 deficiency induced the activation of paneth and goblet cells, increased NF-κB activity, and elevated the levels of proinflammatory genes and antimicrobial proteins in the small intestine.^722^ In addition, SIRT1 activation reduced apoptosis of intestinal epithelial cells via suppression of endoplasmic reticulum stress-mediated apoptosis-associated molecules CCAAT/enhancer-binding protein homologous protein and caspase-12 in both in vivo and in vitro models.^723^ Compared to the studies showing that a decreased expression of SIRT1 might be important in the pathogenesis of IBD, a decrease in SIRT1 might be protective in IBD. SIRT1 deletion might be useful in the improvement of disease conditions in colitis via induction of Foxp3 + T-regulatory cells, which are important for intestinal homeostasis.^724,725^ The loss of SIRT1 in thymic–derived natural Tregs did not affect rescue from autoimmune colitis, although it promoted Foxp3+ development from conventional T cell formation and attenuated autoimmune colitis.^724^ These conclusions suggest that the deleterious role of SIRT1 in immune-related diseases might be related to different T cell sources. Therefore, further studies are needed to verify the association between SIRT1 and more immune cells and to explore the role of SIRT4, which has not been well characterized based on current studies.
2. **Intestinal ischemia/reperfusion injury**
  Studies have demonstrated the beneficial effects of SIRT1, SIRT3, and SIRT6 on intestinal IRI exerted by adjusting ROS generation and massive epithelial apoptosis in a mouse model, which are critical in the pathogenesis of intestinal IRI injury.^229,726,727^ For instance, SIRT1 suppressed epithelial ROS accumulation and apoptosis to attenuate intestinal IRI after miR-34a-5p systematic knockdown.^229^ Resveratrol protected intestinal subacute IRI via the SIRT1-NF-κB pathway in an iNOS-NO-inhibited manner. This might represent a novel prophylactic approach to intestinal IRI.^728^ SIRT3 alleviated intestinal IRI-induced mitochondrial oxidative damage and apoptosis through peroxiredoxin 3 deacetylation, a key protective factor in intestinal IRI.^727^ Moreover, based on both mouse and rat models, downregulating SIRT6 by miR-351-5p aggravated intestinal IRI by promoting oxidative stress, inflammation, and apoptosis.^726^ There have been limited research studies exploring the effect of other SIRTs on intestinal IRI.
3. **Other intestinal diseases**
  In other intestinal diseases, including hirschsprung-associated enterocolitis and necrotizing enterocolitis, SIRT1 was downregulated and involved in inflammation.^729,730^ SIRT1 can be suppressed via miR-132 and miR-212 or downregulated retinoid-related orphan receptor α by exosomal miR-18a-5p and then activate the NF-κB signaling pathway, NLRP3 inflammasome, and caspase-1-mediated pyroptosis, thereby encouraging the inflammatory response in Hirschsprung-associated enterocolitis mice.^729,731^ Similarly, SIRT1 activation might decrease the damage caused by necrotizing enterocolitis by decreasing proinflammatory cytokines and oxidative stress proteins and by increasing the anti‑inflammatory cytokine pathway.^730,732^ SIRT1 alleviated the inflammatory response and intestinal epithelial barrier dysfunction by regulating the expression and inactivation of HIF-1α.^730^
  In conclusion, current studies on intestinal system diseases have mostly focused on IBD. There are limited studies on other SIRTs, except SIRT1, which is a field worthy of further development. In addition, both protective and deleterious effects of SIRT1 have been explored in intestinal diseases. The proinflammatory effects of SIRT1 and regulation of different immune cells might play an important role in aggravating intestinal diseases. As SIRTs have various biological functions in intestinal diseases, they and their underlying mechanisms are promising novel targets for studying the development of intestinal diseases.

##### Conclusions

Most studies on the association between SIRTs and digestive system diseases have been completed in animal models, while the numbers of human studies are increasing. The current evidence demonstrates the role for SIRTs in digestive system diseases and identifies exciting opportunities to adjust SIRT activity to treat or prevent these diseases. Many questions remain unanswered, however, and more research needs to be done, especially on SIRT2, SIRT4, SIRT5, and SIRT7. The potentially divergent roles of different SIRTs in these diseases are not well verified, especially the conflicting roles.

Additionally, SIRT1, the most widely studied SIRT, might have opposing roles in different diseases, particularly in the inflammatory response. The proinflammatory effect of SIRT1 on digestive diseases contrasts with anti-inflammatory effects reported by most studies. These findings are essential because unraveling the less common negative effects of SIRT1 might contribute to a more comprehensive understanding of its generally accepted positive function.

Thus, in the future, more studies on a molecular level and in clinical populations are needed to confirm the role of SIRTs in digestive diseases.

#### SIRTs and nervous system diseases

**Fig. 13:**
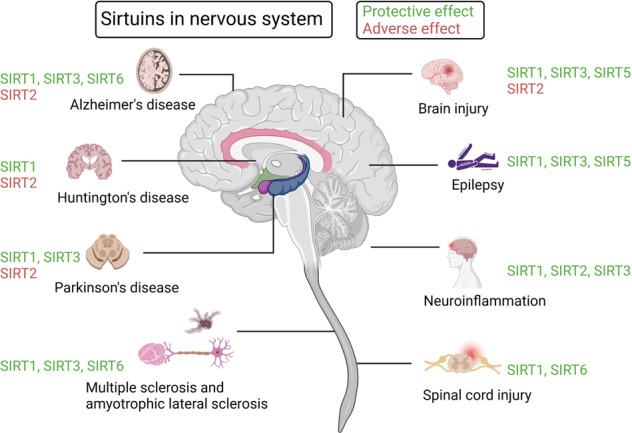
The role of SIRTs in nervous system, mainly including AD, HD, PD, brain injury, epilepsy, neuroinflammation, SCI, multiple sclerosis and ALS. SIRT1, SIRT3, and SIRT6 have protective effects on AD, multiple scierosis and amyotrophic lateral scierosis. SIRT1 plays a major protective role in HD. In PD, SIRT1 and SIRT3 provide protective effects. In addition, SIRT1, SIRT3, and SIRT5 are beneficial in both brain injury and epilepsy. SIRT1, SIRT2, and SIRT3 have roles in protecting against neuroinflammation. SIRT1 and SIRT6 could also protect SCI. Notably, the effects of SIRT2 may be harmful on AD, HD, PD, and brain injury. https://biorender.com

Nervous system diseases directly affect the lives of hundreds of millions of people worldwide,^733^ and one in every nine people dies due to a disorder of the nervous system.^734^ Recently, there has been a gradual increase in research on the role of SIRTs in neurological diseases. An understanding of the latest progress and potential molecular mechanisms of SIRTs in neurological diseases will benefit further studies on the clinical diagnosis and treatment of these diseases. Therefore, this review mainly summarizes the current research progress on the role of SIRTs in neurological diseases (Fig. 13).

##### Alzheimer’s disease (AD)

AD is the most common neurodegenerative disorder that is associated with memory deficit and global cognitive decline.^735,736^ It is a brain disorder associated with gradual weakening of neurocognitive functions, neuroinflammation, and impaired signaling pathways.^737^ The SIRT proteins associated with AD mainly include SIRT1, although mitochondrial SIRTs represented by SIRT3 have also been the focus of research, as well as SIRT6 and SIRT2 that are located in the nucleus and cytosol, respectively.

Amyloid beta (Aβ) is a normal and soluble product of neuronal metabolism,^738^ and Aβ-mediated extracellular senile plaque is regarded as one of the major pathological lesions of AD.^739^ Previous evidence has suggested that SIRTs played important roles in the regulation of Aβ.^739^ For example, in vitro study has shown that overexpression of SIRT1 could reduce Aβ-induced senescence and mitochondrial dysfunctions,^740^ and related mechanism studies have suggested that SIRT1 could regulate Rho-associated kinase 1 or inflammation to attenuate the accumulation of Aβ.^740–742^ Similarly, SIRT3 also protects neurons against Aβ pathology and excitotoxicity.^743^ In contrast, SIRT2 may have adverse effect on Aβ pathology, and in vivo study has revealed that the suppression of SIRT2 deacetylase activity could alleviate Aβ pathology and cognitive deficits in the AD mouse model.^744^ As for the molecular mechanism, SIRT2 could influence the β‐secretase 1 by directly deacetylates reticulon 4B protein, thereby affecting the production of Aβ and ultimately promoting the development of AD.^744^

Tau is the major microtubule-associated protein of a mature neuron, and it is a central molecule in the pathogenesis of AD.^745^ Previous studies have highlighted the importance of PTMs (e.g., O-GlcNAcylation, phosphorylation, and acetylation) of Tau in AD.^746–748^ For example, the level of O-GlcNAcylation of tau in AD brain is reduced, and SIRT1 reduces O-GlcNAcylation of tau through CREB.^749^ Moreover, SIRT2 affects tau phosphorylation and autophagic flux in AD.^750^ There is evidence that tau acetylation occurs in AD brain at early stages of the disorder and that this phenomenon is involved in regulating the early accumulation of tau in AD brain. SIRT3 might play a role in tau acetylation and could be a potential target for developing novel therapies to alleviate tau accumulation.^751^ Collectively, these studies suggest that SIRT1-3 might play a role in PTMs of tau and could be potential targets for designing novel therapies to alleviate tau accumulation in AD brain.

In the healthy brain, high levels of H4K16ac and low levels of SIRT2 coexist with Fzd1 and Fzd7 promoters. A recent study reported a novel role of nuclear SIRT2 in regulating Fzd receptors in AD, wherein nuclear SIRT2 was hyperactivated in AD and FoxO1 recruited SIRT2 to Fzd1 and Fzd7 promoters, leading to a reduction in H4K16ac deacetylation.^752^ These findings suggest that SIRT2 inhibition is an attractive target for ameliorating the pathological effects of AD.

Several studies have shown that autophagy deficits occur in the early stage of AD, which contribute to the process of AD.^753^ SIRT-regulated autophagy impairment plays a key role in the neurodegenerative process of AD. Beclin-1 acetylation impairs the autophagic flux, which contributes to neurodegeneration in AD. Another study showed that SIRT1 enhances the deacetylation of Beclin-1, thus suggesting the beneficial role of SIRT1 in promoting autophagy in AD neurodegeneration.^270^ The loss of function of SIRT2 either through AK1 (a specific SIRT2 inhibitor) or through SIRT2 KO recovers microtubule stabilization and improves autophagy.^299^

AD is manifested through regional cerebral hypometabolism. SIRT3 has emerged as a key regulator of mammalian transcription in response to cellular metabolic state and stress.^754^ Recent studies have shown that SIRT3 dysfunction leads to mitochondrial and neuronal damage in AD, suggesting that SIRT3 has a protective role in hippocampal neurons.^755^ Intermittent food deprivation also reduces neuronal network hyperexcitability and ameliorates deficits in hippocampal synaptic plasticity in a SIRT3-dependent manner in animal models of AD.^756^ Pituitary adenylate cyclase activating polypeptide, a neurotrophin, stimulates mitochondrial SIRT3 production. Knockdown of SIRT3 compromises the neuroprotective effects of pituitary adenylate cyclase activating polypeptide in AD, and this effect was reversed by overexpression of SIRT3.^757^ SIRT3 expression mirrors the spatiotemporal deposition of Aβ in an AD mouse model and is also upregulated in the temporal neocortex of patients with AD.^758^

In clinical research, an inverse relationship was observed between serum levels of SIRT1, SIRT3, and SIRT6 and AD.^759^ Measurement of SIRT1, SIRT3, and SIRT6 levels in saliva could be used as an additional method for intravital noninvasive diagnosis of AD in advanced age patients.^760^ The SIRT protein family constitutes a unique molecular link between aging and human neurodegenerative diseases and offers a promising avenue for therapeutic intervention. However, the mechanisms of action of SIRTs in chronic neurodegenerative diseases in vivo remain unclear. Hence, further studies on the role and mechanism of the SIRT family in AD are required, which could provide promising avenues for therapeutic intervention.

##### Parkinson’s disease (PD)

PD is the most common movement disorder associated with older adults, and currently, there is no effective treatment or prevention methods other than symptomatic treatment. A previous study investigated the possible association of nine SIRT1 and SIRT2 SNPs with the risk of PD through a clinical case-control investigation in Chinese Han population. Further functional assays suggested that rs2015 might influence the expression of SIRT2 by affecting the binding of miR-8061 to the 3ʹ-UTR of SIRT2, eventually contributing to the risk of PD.^761^ Therefore, the SIRT family is involved in the pathology of PD. However, considering that an epidemiological investigation showed that variations in the SIRT genes do not affect the risk for PD,^762^ the association between SIRT gene polymorphisms and PD risk remains elusive and needs further studies to clarify.

The accumulation of misfolded α-synuclein in dopaminergic neurons is the leading cause of PD.^763^ Activated SIRT1 ameliorated LC3 deacetylation-mediated autophagic degradation of α-synuclein and improved motor defects and pathological changes in PD mice.^763^ Moreover, pharmacologically increased levels of SIRT3 could counteract αsyn-induced mitochondrial dysfunction by reducing αsyn oligomers and normalizing mitochondrial bioenergetics, thus supporting a protective role of SIRT3 in PD-associated pathways.^764^ Mitochondrial dysfunction is the main cause of dopaminergic (DAergic) neuronal loss in PD, and SIRT3 plays a key role in regulating mitochondrial function.^765^ The age-dependent elevation of mitochondrial oxidative stress is widely recognized as a major factor in the loss of dopaminergic neurons in the substantia nigra pars compacta in PD, and this process is associated with a decrease in SIRT3 protective function.^766^

SIRT2 also appears to play a different role in PD from other SIRT family members. In vitro and in vivo studies have shown that SIRT2 mediates exacerbation of alpha-synuclein toxicity in models of PD.^298^ NAD + metabolism is altered in sporadic PD patient-derived cells, which contributes to SIRT2 activation and subsequent decrease in the levels of acetylated α-tubulin.^297^ These results suggest that SIRT2 deletion was protective in PD models.

Collectively, these data support a protective role of SIRT1 and SIRT3 in PD-associated pathways, while SIRT2 might show different functions from the former two. Thus, further studies are required to investigate the role of SIRT2 in PD.

##### Huntington’s disease (HD)

HD is an incurable neurodegenerative disorder characterized by movement disorder, psychiatric symptoms, and cognitive decline. Brain-specific KO of SIRT1 results in exacerbation of brain pathology in a mouse model of HD, whereas overexpression of SIRT1 improves survival, neuropathology, and expression of BDNF in HD mice. Mechanistically, mutant huntingtin protein interferes with the CREB-regulated transcription coactivator 1-CREB interaction to repress BDNF transcription, and SIRT1 rescues this defect in vitro and in vivo; this finding suggests a key role of SIRT1 in transcriptional networks in HD brain and offers an opportunity for therapeutic development.^767^

HD has a complex pathogenesis mechanism, including protein aggregation and metabolic dysfunction. SIRT1 expression is increased in HD-affected brain regions, and metabolic pathways are altered in the hypothalamus of individuals with HD.^768^ An important finding is that the manipulation of sterol biosynthesis at the transcriptional level mimics SIRT2 inhibition, which demonstrates that the metabolic effects of SIRT2 inhibition are sufficient to diminish mutant huntingtin toxicity.^769^ This study demonstrated that inhibition of SIRT2 achieves neuroprotection in cellular and invertebrate models of HD. Therefore, both SIRT1 and SIRT2 play an important role in HD, and hence, the effect of SIRTs on HD needs to be further investigated.

##### Brain injury such as IRI and stroke

Brain injury, such as IRI and stroke, is a neurological disorder with high morbidity, high probability of mortality, and poor neurological outcome.^770^ The SIRT family is a highly potent therapeutic target to decrease IRI.^771^ SIRT1 plays an important role in neuroprotection against brain injury through oxidative, inflammatory, autophagy and apoptotic pathways.^214,772,773^ The regulation of autophagy proteins LC3-II and Beclin-1 by NAMPT was abolished in cultured SIRT1-KO neurons, thus suggesting that NAMPT promotes neuronal survival by inducing autophagy in a SIRT1-dependent manner during cerebral ischemia.^774^ SIRT1 deacetylates the RNA-binding protein quaking 6 and activates the transcription factor PGC-1α through post-transcriptional regulation of PPAR-γ expression, which significantly affects the synthesis of triglycerides in neurons of the cerebral IRI rat model, thereby inhibiting neuronal apoptosis.

Several studies have also shown the protective effects of mitochondrial SIRT3 and SIRT5 in IRI. Mitochondrial SIRT3 acts as a prosurvival factor to protect neurons from excitotoxic injury and exerts a protective role in ischemic stroke by regulating the HIF-1α/ vascular endothelial growth factor signaling pathway in astrocytes.^775,776^ Additionally, SIRT3 was found to be downregulated in response to cerebral IRI; therefore, strategies to enhance SIRT3 activity and activate the Wnt/β-catenin pathway could be therapeutic targets for treating cerebral IRI.^777^ SIRT5 has also been shown to mediate IR-induced brain damage by increasing the permeability of blood-brain barrier through degradation of the tight junction protein occluding.^778^

Notably, SIRT2 appears to have detrimental roles in an array of neurological disorders such as PD and HD. The current study demonstrated the neuroprotective effects of SIRT2 inhibition in ischemic stroke and identified the downregulation of the Akt/FoxO3a and MAPK pathways as intermediary mechanisms that might contribute to the reduction in apoptotic cell death by SIRT2 inhibition.^248^ In clinical practice, SIRT2 might serve as a marker of acute ischemic stroke (AIS) risk and prognosis. Serum SIRT2 expression was increased in patients with AIS as compared to that in non-AIS patients with high stroke risk factors. This finding supports the role of SIRT2 in facilitating disease monitoring and prognosis in patients with AIS.^779^

In conclusion, previous studies report the protective roles of SIRT1, SIRT3, and SIRT5 in IRI, while there is also evidence that SIRT2 appears to play a different role in IRI. More studies are required to elucidate the regulatory mechanisms and functional implications of the SIRT family in brain injury.

##### Other diseases

1. **Multiple sclerosis and amyotrophic lateral sclerosis (ALS)**
  Multiple sclerosis is an autoimmune-mediated neurodegenerative disease with characteristic foci of inflammatory demyelination in the brain, spinal cord, and optic nerves.^780^ ALS is also a neurodegenerative disease characterized by degeneration of upper and lower motor neurons, which results in muscle weakness and eventual paralysis, and it is also known as motor neuron disease.^781,782^ To date, ALS remains as an incurable and devastating disease. Drug development efforts are mostly based on SOD1 gene -G93A mice that present a very strong and early phenotype, allowing only a short time window for intervention.^783^ An increased expression of SIRT1 was observed in the cerebral cortex, hippocampal formation, thalamus, and spinal cord of symptomatic SOD1 (G93A) transgenic mice, but the mechanisms and functional implications of increased SIRT1 expression require elucidation.^784^ In human postmortem tissue, increased mRNA and protein levels of SIRT3 were found in the spinal cord in patients with ALS.^785^ Moreover, enhanced SIRT6 activity abrogates the neurotoxic phenotype of astrocytes expressing ALS-linked mutant SOD1, thus indicating that SIRT6 could serve as a potential therapeutic target to prevent astrocyte-mediated motor neuron death in ALS.^786^ These studies illustrated the potential beneficial role of SIRT1, SIRT3, and SIRT6 in ALS.
2. **Epilepsy**
  Epilepsy is a neurological disorder characterized by brain hyperexcitability and manifests as seizure.^787^ SIRT1 might represent a useful therapeutic target to rescue the expression of circadian rhythm genes and sleep patterns in patients with epilepsy.^788^ SIRT5 deficiency strikingly increased the mortality rate and severity of response to epileptic seizures, thus indicating that SIRT5 has a neuroprotective role in epileptic seizures and neurodegeneration.^789^ Mechanistically, SIRT1 protein expression could be inhibited by miR-128, and treatment with the SIRT1 agonist CAY10602 exerts neuroprotective effects on epilepsy.^233^ Similarly, SIRT3 could also protect neurons from kainic acid-induced excitotoxicity by mediating mitochondrial function with enhanced expression by inhibiting miR-134-5p.^790^ Overall, SIRT1, SIRT3, and SIRT5 appear to have a neuroprotective role in epilepsy. More molecular and clinical studies are required in the future to verify the effects of SIRTs on epilepsy.
3. **Cognitive deficits**
  Cognitive deficits are common in patients with conditions such as PD, epilepsy, and psychotic depression.^791–793^ SIRT1 is a recognized longevity gene and has been shown to be associated with aging and its related diseases. SIRT1 is an important protective gene against hippocampal atrophy and its induced cognitive impairment during aging.^794^ Surgery-induced downregulation of hippocampal SIRT1 participates in cognitive impairment after surgery by inhibiting the autophagy process and activating apoptosis.^795^ Additionally, exposure to fluoride could lead to cognitive impairment, and the underlying mechanisms might be related to oxidative stress and mitochondrial dysfunction. Chronic long-term exposure to fluoride causes neural/synaptic damage and cognitive impairment through mitochondrial dysfunction and its associated oxidative stress, which is mediated at least in part by SIRT3 inhibition in mouse brain.^796^ The natural bisphenol compound honokiol upregulated the expression of SIRT3 protein in vivo and in vitro, and its protective effect against oxidative stress and mitochondrial dysfunction could be abrogated by SIRT3 shRNA.^797^ To date, few studies have been conducted on the relationship between SIRTs and cognitive deficits, and more research is required to explore this association.
4. **Spinal cord injury (SCI)**
  SCI is a devastating condition with few effective treatments. Because posttraumatic inflammation contributes to the progression of neuronal degeneration, attenuating inflammation is important for reducing neural degeneration. The anti-inflammatory effect of SIRT1 has been reported to be involved in SCI.^798^ SIRT1 might have a neuroprotective effect by suppressing microglial activation and increasing the secretion of proinflammatory cytokines following SCI.^799^ After the trauma, spinal cord neurons were apparently damaged. Regulation of autophagy by the AMPK/SIRT1 pathway could restrain the damage of spinal cord neurons, which might be a potential intervention for SCI.^286^ SIRT6 might also play a vital role in the pathogenesis of SCI. Mechanistically, the upregulation of SIRT6 alleviated inflammation and oxidative stress and inhibited cell apoptosis in SCI.^800^ In terms of mechanism, multiple miRNAs such as miR-138-5p, miR-324-5p, and miR-30c have been reported to be involved in SCI by targeting SIRT1.^801,802^ These studies provide a promising biomarker of prognosis and therapy for spinal cord diseases.
5. **Neuroinflammation and neuropathic pain**
  Diverse causes of neuropathic pain are associated with excessive inflammation in both the peripheral and central nervous system, which might contribute to the initiation and maintenance of persistent pain.^803^ SIRTs might serve as a potential therapeutic target for treating neuropathic pain. SIRT1 and SIRT2 deacetylases are reported to exert neuroprotective effects on neuroinflammation.^61^ SIRT1 activation attenuated Mn-induced oxidative stress and neuroinflammation in adult mice.^804^ Overexpression of SIRT2 alleviates neuropathic pain and neuroinflammation.^805^ The SIRT2 inhibitor AK-7 exacerbates traumatic brain injury through a potential mechanism involving increased acetylation and nuclear translocation of NF-κB p65, resulting in the upregulation of NF-κB target genes and proinflammatory cytokines.^88^ Another study suggested a key protective role of microglial SIRT2 in amnesic deficits associated with neuroinflammation.^806^ SIRT2-deficient mice (SIRT2(−/−)) showed morphological changes in microglia and an increase in proinflammatory cytokines upon intracortical injection of LPS.^807^ SIRT3 also regulates mitochondrial oxidative stress response and neuroinflammation. SIRT3-induced Mst1-JNK-SRV2 signaling pathway protected against neuroinflammation-mediated cell damage in BV-2 microglia.^808^ LPS induces oxidative stress and neuroinflammation in BV2 cells, which might be mediated in part by the downregulation of triggering receptor expressed on myeloid cells 2 and SIRT3. Triggering receptor expressed on myeloid cells 2 overexpression ameliorates LPS-induced oxidative stress and neuroinflammation by enhancing SIRT3 function through NAD + .^809^ Here, SIRT1-3 show anti-neuroinflammatory effects. More research is required to elucidate whether these SIRTs affect neuroinflammation and neuropathic pain through the same or different mechanisms.

##### Conclusion

An increasing number of studies predict that the effects of various SIRTs on neurological diseases might be different or even contrasting. However, it is worth noting that positive intervention of SIRT activity, such as through upregulation of SIRTs-activating molecules, might have profound therapeutic benefits on various nervous system diseases. The long-term effects of decreased SIRT levels per se or in chronic neurodegenerative conditions is an important question for future studies. Therefore, in the research process, we should discover new mechanisms of action to elucidate the different results. Additionally, research should be based not only on cellular and animal models but also on the relationship between inflammatory SIRTs and diseases from human epidemiology.

#### SIRTs and endocrine system diseases

**Fig. 14:**
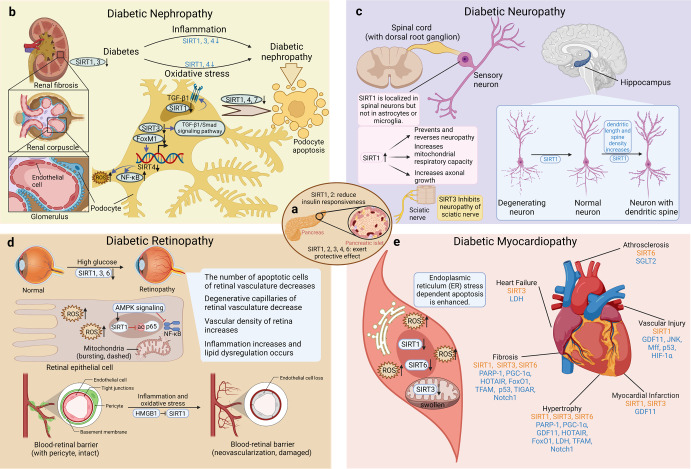
The roles of SIRTs in diabetes and related target organs injury. **a** SIRT1, SIRT2, SIRT3, SIRT4, and SIRT6 are associated with pathological processes in the occurrence and development of DM. SIRT1 and SIRT2 have dual functions, including both improving insulin sensitivity and reducing insulin responsiveness. SIRT3, SIRT4, and SIRT6 mainly exert protective effect on DM. **b** SIRT1, SIRT3, SIRT4, and SIRT7 play protective roles in diabetic nephropathy. Low levels of SIRT1 and SIRT3 are associated with renal fibrosis and reduced expressions of SIRT1, SIRT4, and SIRT7 are related to podocyte apoptosis. **c** Increasing SIRT1 expression can exert protective effect during the development of neuropathy in sensory neuron of spinal cord. Moreover, SIRT1 could also reverse neuron damage in hippocampus. In addition, SIRT3 may inhibit neuropathy in sciatic nerve. **d** SIRT1, SIRT3, and SIRT6 are reduced in the pathological process of diabetic retinopathy. Additionally, SIRT1 is reduced during the damage of blood-retinal barrier. **e** SIRT1, SIRT3, and SIRT6 act protective roles in the development of diabetic cardiomyopathy, which consists of heart failure, cardiac fibrosis, cardiac hypertrophy, myocardial infarction, vascular injury and atherosclerosis. https://biorender.com. HOTAIR HOX transcript antisense RNA, Mff mitochondrial fission factor, SGLT2 sodium-dependent glucose transporters 2

Endocrine system regulation is important for the maintenance of homeostasis, and it controls hormonal functions under physiological conditions and behaviors as well as adaptations to social environments.^810^ Endocrine system disorders lead to various diseases such as diabetes mellitus (DM), obesity, and metabolic syndrome, which causes heavy disease burden worldwide.^811^ The activation of SIRT proteins enhances metabolic efficiency and upregulates mitochondrial oxidative metabolism, which are important for metabolic balance of human body.^46^ A growing number of studies have shown that SIRTs exert vital effects on maintaining metabolic health and controlling the occurrence and development of endocrine system diseases such as DM (Fig. 14),^128,812^ diabetic complication (Fig. 14),^813–815^ obesity,^816^ and metabolic syndrome.^817^

##### DM

Globally, more than 425 million people are living with DM, and its prevalence is expected to increase at least 50% by 2045.^818^ Worldwide, DM is the leading cause of blindness, nontraumatic lower extremity amputations, peripheral neuropathy, and end-stage kidney diseas.^819–822^ Numerous reports have suggested that SIRTs, especially SIRT1-3, SIRT5, and SIRT6, are associated with biological processes that participate in the development and progression of diabetes, such as glucose metabolism, mitochondrial function, and resistance against cellular stress.^26,823,824^ The expression of SIRTs in patients with DM has been reported inconsistently. The expression of SIRT1-3 is reduced in patients with DM,^825–827^ while the expression of SIRT5 and SIRT6 is elevated.^350,828^ Thus, the altered expression of SIRT proteins might affect the progression of DM.

SIRT proteins play important roles in the occurrence and development of DM by regulating glucose metabolism and maintaining insulin homeostasis.^6^ SIRT1 and SIRT2 have been found to have dual function in the development of DM, which might be due to the biological process occurring in the cells from different types of tissues or organs. For instance, SIRT1 overexpression could improve insulin sensitivity and reduce insulin resistance,^829,830^ while the downregulation of SIRT1 inhibits insulin-stimulated glucose transport in adipocytes in particular by inhibiting insulin signaling.^831^ Conversely, hepatic SIRT1 knockdown prevented fasting hyperglycemia by decreasing hepatic glucose production and increasing hepatic insulin responsiveness.^832^ SIRT2 could also promote glucose-dependent hepatic glucose uptake by deacetylating K126 of glucokinase regulatory protein.^833^ In contrast, the downregulation of SIRT2 ameliorated the reduced activity of Akt and increased insulin-stimulated glucose uptake in insulin-resistant neuro-2a cells.^834^ However, the detailed molecular mechanisms of these bilateral roles remain unclear and need further investigation.

SIRT3, SIRT4, and SIRT6 have been proven to exert a protective effect on DM. For example, SIRT3 KO severely impaired insulin-stimulated muscle glucose uptake, which further aggravated insulin resistance.^812^ Likewise, SIRT4 overexpression led to dyslipidemia, lipogenesis, and decreased fatty acid oxidation; this might be because SIRT4 can deactivate AMPK as well as directly inhibit insulin secretion at the cellular level.^835^ Moreover, SIRT6 induced PGC-1α acetylation and suppressed hepatic glucose production,^836^ and SIRT6 cooperated with p53 to deacetylate FoxO1 and transport FoxO1 from the nucleus to the cytosol, and suppressed the expression of gluconeogenic genes,^837^ all of which could alleviate diabetic hyperglycemia. Conversely, SIRT5 can promote the progression of DM. Experiments in two pancreatic β-cell lines (MIN6 and INS-1) suggest that SIRT5 inhibition facilitated pancreatic β-cell proliferation and insulin secretion.^350^ Moreover, SIRT5 negatively regulates the transcription of PDX1 through its deacetylase activity,^350^ and subsequently, the downregulation of PDX1 expression aggravates DM.^838,839^ These studies suggest that high expression of SIRT3, SIRT4, and SIRT6 and low expression of SIRT5 might exert protective effects on the development of DM.

In conclusion, there is limited research on the relationship between SIRT proteins and DM; most studies have shown that SIRT1-4 and SIRT6 exert protective effects on the development of DM, while SIRT5 promotes the progression of DM. However, other studies have found the downregulation of SIRT1 and SIRT2 contributes to improve DM. The difference in the effect of SIRT1 and SIRT2 on DM might be attributed to cells from different types of tissues or organs and required to be further clarified. Additionally, future studies could pay more attention to the role of SIRT proteins, especially SIRT7, in the development and progression of DM.

##### DM-related organ damage

The global epidemic of DM has led to a corresponding epidemic of complications of these disorders.^840^ Devastating macrovascular complications CVD and microvascular complications [such as diabetic kidney disease (DKD), diabetic retinopathy (DR), and diabetic neuropathy (DN)] lead to increased mortality, blindness, kidney failure, and an overall decreased quality of life in individuals with DM.^841^ SIRTs have been shown to have protective effects on the target organ damage caused by DM, such as diabetic cardiomyopathy (DCM),^842^ DKD,^814,815^ DR,^129^ and DN.^843^

1. **DKD**
  DKD is recognized as a severe complication of DM and a dominant pathogeny of end-stage kidney disease, which causes severe health problems and large financial burden worldwide.^844^ During the past two decades, the morbidity and mortality of DKD have been rising rapidly worldwide,^845^ and the age-standardized prevalence of DKD in men and women was 15.48/1000 and 16.50/1000, respectively, in 2017.^846^ SIRT1 shows a protective role in the development of DKD. In detail, high expression of SIRT1 effectively protects the kidney and slows down the progression of DKD.^814,815^ On the one hand, increased SIRT1 activity protects against DM-induced podocyte injury and effectively mitigates the progression of DKD.^814^ On the other hand, stimulation of SIRT1 expression and signaling in DM protects the kidney against oxidative stress and nephropathy.^815^ Mechanistically, SIRT1 exhibited its renal protective effects through deacetylation of the transcription factor p53^815^ and activation of the transcription factors FoxO3a and Nrf2.^815,847^ For example, SIRT1 attenuated nephropathy progression in diabetic mice by downregulating acetylated p53 expression and upregulating FoxO3a expression.^815^ Moreover, increasing SIRT1 activation by resveratrol in both in vivo and in vitro studies promoted resistance to diabetic renal fibrosis by activating Nrf2, a leucine transcription factor.^847^ SIRT1 can effectively reduce the damage caused by DKD and slow down the progression of DKD.^814,815^ Therefore, SIRT1 might become a potential target for the clinical treatment of DKD.
2. **DN**
  DN is the most prevalent diabetic complication, and at least 50% of individuals with diabetes develop DN over time.^840^ It substantially affects patients by increasing falls, thereby causing pain and reducing the quality of life.^848^ Accumulating evidence has demonstrated that SIRT1 modulates neuronal viability,^849^ neuronal differentiation,^849^ and synaptic plasticity,^850^ all of which are key factors largely linked to cognitive improvement. SIRT1 has also been proved to alleviate symptoms related to DN, including cognitive decline,^843^ neuropathic pain,^851^ and peripheral neuropathy.^852^ For instance, SIRT1 expression was decreased in the hippocampus of diabetic rats, which reduced dendritic length and spine densities and decreased TORC1, p-CREB, and BDNF protein levels, resulting in diabetes-related cognitive decline.^843^ Moreover, the upregulation of spinal SIRT1 relieved pain behavior, inhibited enhanced structural synaptic plasticity in diabetic rats and mice with diabetic neuropathic pain, and decreased the levels of synapse-associated proteins in diabetic neuropathic pain rats, diabetic mice, and high glucose-cultured spinal neurons.^851^ SIRT1 also regulated mitochondrial function in the peripheral nerve through PGC-1α, and the failure of the SIRT1-PGC-1α-mitochondrial transcription factor A (TFAM) signaling axis might result in the suppression of mitochondrial oxidative phosphorylation and development of peripheral neuropathy.^852^ Collectively, an understanding of the regulatory roles of SIRT1 proteins might help to develop them as promising therapeutic targets in DN treatment. However, recent studies mainly focus on SIRT1, and the molecular mechanisms of other SIRT proteins in regulating DN are still unclear and need further investigation.
3. **DR and DCM**
  DR is a common and specific microvascular complication of DM and remains the leading cause of preventable blindness in working-aged people.^853^ It is identified in one third of patients with DM and is associated with increased risk of life-threatening systemic vascular complications, including stroke, coronary heart disease, and heart failure.^853^ Current studies have shown that SIRT1 can alleviate DR;^129^ however, related studies are still limited. Previous studies have revealed that overexpression of SIRT1 prevents the increase in capillary cell apoptosis and formation of degenerative capillaries,^854^ reduces DM-induced inflammation in the retina, and improves DM-induced visual function impairment.^129^
  DCM is also a distinct form of heart disease that represents a major cause of death and disability in patients with diabetes, particularly in the more prevalent type 2 diabetes patient population.^855^ The activation of SIRT1 and SIRT3 contributes to inhibit the development of DCM. For example, SIRT1 activation inhibits ROS generation-induced oxidative stress and fibrosis, thereby attenuating DCM.^856^ The activation of SIRT3 also regulates fibrosis, inflammation, apoptosis, and oxidative stress in diabetic myocardial tissue^149^ and attenuates DCM through the reduction in p53 acetylation and TP53-induced glycolysis and apoptosis regulator expression together with upregulation of 6-phosphofructo-2-kinase/fructose-2,6-bisphosphatase isoform 3, which are the key regulators of phosphofructokinase and glycolysis.^842^ In contrast, SIRT3 deficiency aggravated hyperglycemic mitochondrial damage, increased ROS accumulation, promoted necroptosis, possibly activated the NLRP3 inflammasome, and finally exacerbated DCM in mice.^857^
  Therefore, SIRT1 and SIRT3 show positive effects in a variety of diabetic complications, including DKD, DN, DR, and DCM, which indicated that these two SIRTs could serve as promising therapeutic targets in the clinical treatment of DM-related target organ damage. However, the molecular mechanisms of other SIRTs in regulating diabetic complications are not fully understood and require further studies.

##### Obesity

The Global Burden of Disease Obesity Collaborators have estimated that more than 603.7 million adult individuals are obese.^858^ Elevated body mass index values were responsible for 4 million deaths in 2015.^858^ Severe obesity is associated with a state of chronic inflammation,^859^ which results in an increase in the incidence of type 2 diabetes, CVD, hepatic steatosis, airway disease, neurodegeneration, biliary disease, and certain cancers.^860^ These obesity-associated disorders are subsequently linked to reduced life expectancy and premature death.^861^ SIRTs act as deacetylases that could affect a variety of metabolic and inflammatory pathways, potentially improving health and extending lifespan.^816^ Therefore, SIRT proteins might play an important role in controlling obesity and reducing other diseases caused by obesity.

Accumulated evidence suggests that SIRT1 and SIRT3 could suppress obesity by inhibiting adipogenesis and stimulating energy expenditure.^862–864^ The 3ʹ-UTR of SIRT1 mRNA binds directly to miR-146b and promotes adipogenesis through SIRT1 downregulation,^862^ while inhibition of hypothalamic SIRT1 enhanced the activity of the hypothalamic-pituitary-thyroid axis, which stimulated energy expenditure.^863^ Moreover, high expression of SIRT1 and PGC-1α activated by AMPK subsequently increased citrate synthase activity and improved muscle mitochondrial respiration on a fatty acid-derived substrate.^865^ The increased expression of SIRT1 similarly reduced acetylation of PGC-1α and FoxO1, which was associated with attenuation of high fat diet-induced mitochondrial dysfunction, insulin resistance, and obesity.^866^ Additionally, overexpression of SIRT3 activated macroautophagy by activating the AMPK-ULK1 pathway, leading to smaller lipid droplet size and reduced lipid accumulation. Similarly, SIRT3 overexpression induced the formation of perilipin-1-heat shock cognate 71-kDa protein-lysosome-associated membrane protein 2 complex to activate chaperone-mediated autophagy and cause instability of lipid droplets in adipocytes.^864^

In contrast, SIRT2 and SIRT6 promote the occurrence and development of obesity. The SIRT2- PGC-1α regulatory axis is negatively regulated by HIF-1α, which negates the intrinsic pathways of fatty acid catabolism in adipocytes and creates a metabolic state that supports the development of obesity.^867^ SIRT6 overexpression was found to exacerbate diet-induced obesity by decreasing STAT3 acetylation and lowering pro-opiomelanocortin expression in the hypothalamus.^868^

Overall, these findings suggest that high expression of SIRT1 and SIRT3 and low expression of SIRT2 and SIRT6 produced a metabolic state that inhibited the development of obesity, thereby reducing the occurrence of obesity. Therefore, the strategy of developing SIRT activators/inhibitors has important clinical significance to prevent obesity and control the occurrence and development of obesity and related diseases.

##### Other metabolic disorders

SIRT proteins are correlated with the occurrence and development of other metabolic diseases. The expression of SIRT1 and SIRT6 is downregulated in lipid metabolism-related diseases,^869,870^ and the expression of SIRT1 is downregulated in metabolic syndrome,^178^ which exerts an adverse effect on metabolic health.

SIRT1 and SIRT6 exert a crucial effect on lipid metabolism and are involved in the improvement of hepatic steatosis and hypercholesterolemia by inhibiting inflammation and promoting histone deacetylation.^869,870^ For instance, modest overexpression of SIRT1 shows lower lipid-induced inflammation and almost entirely protects from hepatic steatosis by induction of antioxidant proteins MnSOD and Nrf1, possibly through stimulation of PGC-1α and lower activation of proinflammatory cytokines such as TNF-α and IL-6 through downregulation of NF-κB activity.^869^ SIRT6 overexpression improves hypercholesterolemia in diet-induced or genetically obese mice, and the underlying biological mechanism might be due to the recruitment of SIRT6 by FoxO3 to the SREBP2 gene promoter where SIRT6 deacetylates histone H3 at lysine 9 and 56, thereby promoting a repressive chromatin state.^870^

Moreover, SIRT1 could confront metabolic syndrome by inhibiting inflammation. Mechanistically, post-transcriptional stabilization of SIRT1 by HuR repressed inflammation and hyperglycemia and induced E-selectin release and endothelial cell activation to counter metabolic syndrome.^817^ These findings show the protective roles of SIRT1 and SIRT6 in the development of various metabolic disorders. Although limited studies have been conducted on this topic, the modulation of SIRT proteins is thought to play a crucial role in the development and progression of metabolic disorders and is expected to be a therapeutic strategy of metabolic disorders.

##### Conclusion

In this section, we have reviewed the role of different SIRT proteins in diverse endocrine system diseases, and current studies are mainly focused on SIRT1-3 and SIRT6. Generally, SIRTs play protective roles in the occurrence and progression of a variety of endocrine system diseases. Of note, SIRT1 and SIRT2 exert a dual effect on the progression of DM, while SIRT6 overexpression exacerbates diet-induced obesity. Therefore, clarifying the specific mechanism of SIRT1 and SIRT2 in DM or revealing the mechanisms underlying their different effects might be of great significance for the clinical treatment of DM. Overall, SIRT proteins are promising therapeutic targets, and the pharmacological modulation of SIRTs could be used to prevent and treat endocrine system diseases.

#### SIRTs and urogenital system diseases

**Fig. 15:**
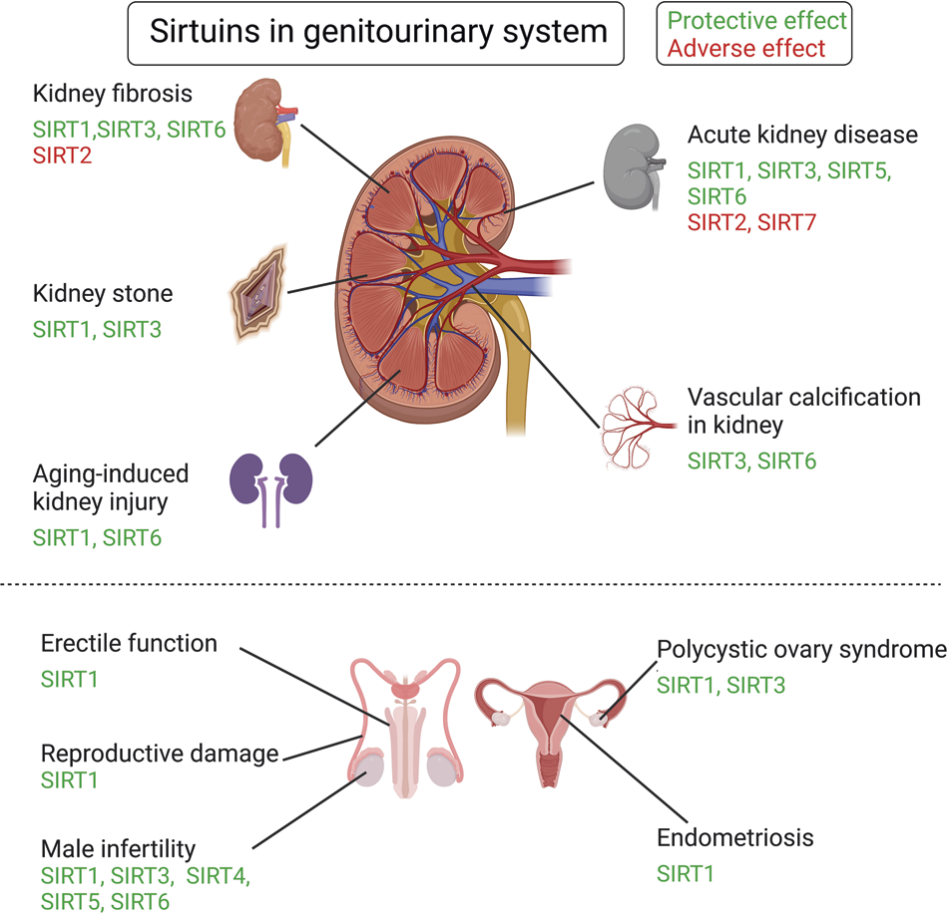
The roles of SIRTs in genitourinary system. SIRT protein family is involved in common of urogenital system including acute kidney disease, CKD (such as kidney fibrosis, kidney stone, aging-induced kidney injury, and vascular calcification in kidney), and genital system disease (mainly including erectile function, reproductive damage, male infertility, PCOS and endometriosis). SIRT1 play a protective effect in aforementioned disease. Moreover, the positive effects of SIRT3 and SIRT6 have been demonstrated in acute kidney disease, kidney fibrosis, vascular calcification in kidney, and male infertility. However, SIRT3 also play a protective role in kidney stone and PCOS, and SIRT6 is protectively associated aging-induced kidney injury. Besides, SIRT4-5 contribute to the remission of male infertility. Additionally, SIRT2 and SIRT7 can aggravate the occurrence of acute kidney disease, and SIRT2 also can aggravate the occurrence of kidney fibrosis. https://biorender.com

Urogenital system diseases include both urinary system diseases and genital system diseases which can contribute to the loss of some physiological functions, including reabsorption of nutrients, regulation of the balance of electrolytes and fluid, maintenance of acid–base homeostasis, and sexual reproduction.^871,872^ Thus, urogenital system diseases impose a serious economic and health burden on human development. Increasing evidence suggests that SIRT protein family activity and expression are associated with the occurrence and progression of various urogenital system diseases.^873–876^ Kidney disease is the most common urinary system disease, and can be divided into acute kidney disease and chronic kidney disease (CKD) according to the disease state.^877^ Therefore, in this section, we focus on the associations between the SIRT protein family and AKI, CKD, and genital system diseases (Fig. 15).

##### Acute kidney injury (AKI)

AKI is defined by a sudden loss of excretory function, in which slow deterioration of kidney function or persistent kidney dysfunction is associated with an irreversible loss of kidney cells and nephrons, which could lead to CKD.^878,879^ AKI mostly occurs as a complication of a single disease with a pooled incidence and mortality rate of 21%, respectively, and the incidence of AKI in intensive care units has increased in world regions over the past decades due to aging populations.^880–882^ Therefore, it is important to determine the molecular biological mechanisms of AKI.

Growing evidence has suggested that mitochondrial dysfunction is a major contributor to AKI.^151,254,883–885^ SIRT3-5 proteins, which are expressed in mitochondria, seem to play an important protective role in AKI.^886^ Among these, the protective role of SIRT3 has been reported to be related to improving mitochondrial function and ultimately improving apoptosis and eliminating ROS.^151,254,883–885^ For example, a sepsis-induced AKI model was constructed in wild-type and SIRT3 systematic KO mice. The results suggest that SIRT3 deficiency exacerbated histopathological and mitochondrial damage to the proximal tubules of the kidney. In addition, systematic KO of SIRT3 resulted in a significant increase in the apoptosis of kidney tubular epithelial cells, increased mRNA levels of Bax and Caspase-3, and decreased mRNA levels of Bcl-2.^254^ A previous study also demonstrated similar results, as SIRT3 deletion aggravated fatty acid oxidation dysfunction, resulting in increased apoptosis of kidney tissues and aggravated renal injury. Also, the activation of SIRT3 by honokiol increased ATP production, and reduced ROS and lipid peroxidation by improving mitochondrial function.^151^ Moreover, the overexpression of SIRT3 improved kidney function, modulated oxidative injury, repressed inflammatory damage, and reduced tubular epithelial cell apoptosis. SIRT3 overexpression attenuated ischemia-reperfusion-induced mitochondrial damage in renal tubular epithelial cells, as evidenced by decreased ROS production, increased antioxidant-sustained mitochondrial membrane potential, and inactivated mitochondria-initiated death signaling.^885^

Compared with the effects of SIRT3, although several studies have suggested the protective effect of SIRT1,^887–889^ the potential molecular mechanism regarding SIRT1 was inconsistent. More studies focused on different signaling pathways related to SIRT1 (such as the JNK signaling pathway and SIRT1/p53 up-regulated modulator of apoptosis/FoxO3a) rather than specific pathways. For example, a study suggested that, in vitro, SIRT1 attenuated the stress response by modulating the JNK signaling pathway, probably via deacetylation of the JNK phosphatase, DUSP16 of AKI.^887^ A previous study found that SIRT1/p53 up-regulated modulator of apoptosis/FoxO3a deacetylation by depleting miR-183-3p could improve renal tubulointerstitial fibrosis after AKI.^873^ Furthermore, kidney ischemia/reperfusion injury, which is a major cause of AKI, is associated with decreased AMPK phosphorylation and a five-fold increase in kidney SIRT1 expression. Activators of kidney AMPK might thus represent a novel therapeutic approach in patients susceptible to AKI.^889^ Moreover, the protection of NAD + in AKI is associated with SIRT1 expression and acts in a SIRT1-dependent manner. The NAD + /SIRT1/glycogen synthase kinase-3β/Nrf2 axis is an important mechanism that could protect against AKI and might be a potential therapeutic target in the treatment of AKI.^890^

There have been limited studies carried out on the associations between SIRT2, SIRT5-7 and AKI. Acetylation of MAPK phosphatase-1 was significantly increased in SIRT2-knockdown cells and decreased in SIRT2-overexpressed cells after cisplatin stimulation. SIRT2 systematic KO mice and SIRT2 transgenic mice showed amelioration and aggravation of renal injury, apoptosis, necroptosis, and inflammation induced by cisplatin.^891^ In addition, overexpression of SIRT5 and SIRT6 can repair kidney damage. For example, SIRT5 regulates the balance of mitochondrial versus peroxisomal fatty acid oxidation in proximal tubular epithelial cells to protect against AKI,^892^ and SIRT6 overexpression inhibited apoptosis induced by LPS and promoted autophagy in HK-2 cells.^312^ Previous studies found that SIRT7 deficient mice were protected against AKI, suggesting that this HDAC promotes tubular damage and kidney inflammation.^893^

In conclusion, the SIRT protein family could play an important role in AKI by regulating multiple cellular and physiologic processes including, apoptosis, oxidative stress, and mitochondrial function. Therefore, exploring treatment strategies using the SIRT protein family in AKI is a promising area.

##### Chronic kidney disease

CKD is characterized by progressive kidney dysfunction of at least three months duration, it affects about 10% of adults worldwide, and is ranked fourteenth in the list of leading causes of death.^894–896^ According to the World Health Organization estimates, 864,226 deaths (or 1.5% of deaths worldwide) were attributable to CKD in 2012.^896^ CKD arises from many heterogeneous disease pathways that alter the function and structure of the kidney irreversibly, over months or years. Diabetes and hypertension are the main causes of CKD in all high-income and middle-income countries, and many low-income countries.^896^ We describe details of associations between the SIRT protein family and both diabetic nephropathy and hypertensive nephropathy in the sections on endocrine system disease and cardiovascular system disease, respectively. In this section, we introduce the effects of the SIRT protein family on other types of CKD, including kidney fibrosis, kidney stones, aging-induced kidney injury, and vascular calcification (VC) in the kidney.

1. **Kidney fibrosis**
  Regardless of the initial cause of disease, kidney fibrosis is the final common pathway in the evolution of virtually all types of CKD, which could contribute to loss of kidney functions (such as filtering and a reabsorbing).^874,897,898^ Thus, kidney fibrosis remains an important clinical problem in both developed countries and developing nations.^899^ In 2001, more than 400,000 patients were receiving treatment for chronic kidney failure in the United States, and the cost of treating this problem was approximately $22.8 billion.^900^ Therefore, we discuss the functions and molecular mechanisms of the SIRT protein family in kidney fibrosis to prevent and reduce the disease burden.
  Among the mechanisms responsible for kidney fibrogenesis, the TGF-β signaling pathway is known to play a pivotal role in kidney tubulointerstitial fibrosis, which stimulates autocrine and paracrine released connective tissue growth factor (CTGF).^901,902^ Previous studies reported the effects of the SIRT protein family on TGF-β signaling pathway in kidney fibrosis.^903–907^ For example, overexpression of SIRT1 abolished TGF-β1-induced cell apoptosis and fibrosis, and suppressed CTGF expression via stimulation by TGF-β1 in mouse kidneys with unilateral ureteral obstruction (UUO).^903^ Similarly, a previous study also investigated the role of the SIRT1 activator, SRT1720, in UUO-induced tubulointerstitial fibrosis. The administration of SRT1720 increased SIRT1 levels and partially attenuated UUO-induced kidney fibrosis and apoptosis, and inhibited the levels of TGF-β1/CTGF.^904^ Moreover, genetic knockdown and chemical inhibition of SIRT2 attenuated TGF-β1-induced fibroblast activation and mouse double minute 2 protein expression.^905^ Furthermore, SIRT3 KO mice were susceptible to hyper-acetylated mitochondrial proteins and to severe kidney fibrosis. Pyruvate dehydrogenase E1α, which is the primary link between glycolysis and the tricarboxylic acid cycle, is hyper-acetylated at lysine 385 in tubular epithelial cells after TGF-β1 stimulation and is regulated by SIRT3.^906^ With regard to SIRT6, a study investigated the effect of proximal tubule-specific SIRT6 KO on UUO-induced kidney tubulointerstitial inflammation and fibrosis which suggested that the SIRT6 activator MDL-800 mitigated UUO-induced kidney tubulointerstitial inflammation and fibrosis. In an in vitro experiment, MDL-800 decreased the TGF-β1-induced activation of myofibroblasts and ECM production by regulating SIRT6-dependent β-catenin acetylation and the TGF-β1/Smad signaling pathway.^907^ The identification of strategies to prevent and/or treat fibrotic CKD is a daunting challenge, and no treatment is specifically targeted at kidney fibrosis.^908^ The effects of the SIRT protein family on the TGF-β signaling pathway may identify new targets for therapeutic intervention in kidney fibrosis.
  In addition, apart from the TGF-β signaling pathway, other molecular mechanisms of kidney fibrosis regarding the effects of the SIRT protein family were also investigated. For example, SIRT1 attenuated kidney fibrosis by repressing HIF-2α;^813^ Endothelial SIRT1 deficiency induced nephrosclerosis through downregulation of matrix metalloproteinase-14, and restoration of matrix metalloproteinase-14 expression in SIRT1-depeleted mice improved the angiogenic and matrilytic functions of the endothelium, prevented kidney dysfunction, and attenuated nephrosclerosis;^909^ SIRT1 inhibited Ang II type 1 receptor and NF-κB expression in kidney fibroblasts and these mechanisms might play roles in alleviating UUO-induced damage.^910^
  It is worth noting that downregulation of SIRT1 and SIRT2 might inhibit kidney interstitial fibroblast activation and attenuate kidney interstitial fibrosis in obstructive nephropathy. SIRT1/2 activity may contribute to kidney fibroblast activation and proliferation as well as kidney fibrogenesis through activation of epidermal growth factor receptor and platelet-derived growth factor receptor-β signaling. Blocking SIRT1/2 activation might have therapeutic potential for the treatment of CKD.^911^
  Collectively, most studies showed that SIRT1-3 and SIRT6 were protective in the development of kidney fibrosis. However, one study showed that the downregulation of SIRT1 and SIRT2 contributed to improving this disease. The specific mechanism of these different effects of SIRT1 and SIRT2 on kidney fibrosis requires further clarification by more intensive studies.
2. **Other chronic kidney injuries**
  With regard to other chronic kidney injuries, a previous study mainly focused on the effects of the SIRT protein family on kidney stones, aging-induced kidney injury, and VC in the kidney.
  Limited studies have indicated that kidney stones showed downregulated expression of SIRT3 and SIRT1. Human peripheral blood monocytes from patients with kidney stones showed decreased SIRT3 expression, but increased FoxO1 acetylation compared with the normal controls,^51^ and the protective effect of SIRT3 could be mediated by activation of the nuclear factor erythroid 2-related factor/heme oxygenase-1 pathway.^912^ A previous study suggested that suppressing SIRT1 expression promoted calcium oxalate monohydrate-induced crystal-cell adhesion and exacerbated cell injury.^913^
  Evidence of the effect SIRT1 and SIRT6 on aging-induced kidney injury is limited. SIRT1-induced deacetylation of HIF-1α might have protective effects against tubulointerstitial damage in aged kidney.^914^ A previous study found that reduction of podocyte SIRT1 led to aggravated aging-induced glomerulosclerosis and albuminuria. At the molecular level, knockdown of SIRT1 in podocytes was associated with reduced activation of the transcription factors PGC-1α/PPARγ, FoxO3, FoxO4, and p65 NF-κB, through SIRT1-mediated deacetylation.^915^ Moreover, SIRT6-deficient mice exhibited kidney hypertrophy with glomerular enlargement and proteinuria. In vitro, knockdown of SIRT6 in cultured primary murine podocytes induced shape changes with loss of process formation and cell apoptosis.^916^
  VC is common in CKD and contributes to CVD. At the molecular level, soluble epoxide hydrolase interacted with SIRT3, which might destabilize SIRT3 and accelerate the degradation of SIRT3. Deletion of soluble epoxide hydrolase might preserve the expression of SIRT3, and thus maintain mitochondrial ATP synthesis and morphology, significantly suppressing calcification of VSMCs.^875^ In addition, SIRT6 is markedly downregulated in patients with CKD and VC. At the molecular level, SIRT6 suppressed the osteogenic transdifferentiation of VSMCs via regulation of runt-related transcription factor 2.^917^ A previous study also indicated that bone marrow mesenchymal stem cell-derived exosomes inhibited high phosphate-induced aortic calcification and ameliorated renal function via the SIRT6-high mobility group box 1 deacetylation pathway.^918^
  The pathophysiology of CKD is complex and the etiologies diverse. There are still various unexplored associations between the SIRT protein family (such as SIRT2, SIRT4, SIRT5, and SIRT7) and different CKDs. Thus, these associations require more in-depth investigation. It could be implied that SIRT1 is an important survival factor and a potential therapeutic target in CKD.

##### Genital system diseases

To date, only a few studies have explored the SIRT protein family and genital system diseases. Two studies have emphasized the protective effect of resveratrol in erectile function and reproductive damage (caused by nicotine), which could positively modulate SIRT1.^919,920^ Moreover, through an improved level of SIRT1, polyunsaturated fatty acids supplementation attenuates oxidative damage in testis by reinforcing the antioxidant defense system.^876^ In addition, to investigate SIRT1 regarding adjuvant strategies in the treatment of male infertility, dysregulation of SIRT1 and mitochondrial SIRT (SIRT3-5) genes were associated with human male infertility.^921,922^ In female genital system disease, a limited number of studies have paid attention to polycystic ovary syndrome (PCOS) and endometriosis. PCOS patients had higher SIRT1 levels than healthy controls^923^ and involvement of the SIRT1/AMPK axis in autophagy activation in PCOS.^924^ SIRT3 deficiency in granulosa cells of PCOS patients might potentially induce impaired oocytes in PCOS.^925^ Furthermore, previous studies only explored the association between SIRT1 and endometriosis. For example, one study suggested that SIRT1 was over-expressed in eutopic endometrium of women with endometriosis and likely participates in the pathogenesis of endometriosis.^926^ Another two studies demonstrated that resveratrol has therapeutic potential^601^ and miRNA-34a^927^ might provide a potential biomarker for endometriosis therapeutics.

In summary, current studies on the SIRT protein family and genital system disease are still in their infancy, and more research is needed in the future to explore these associations.

##### Conclusion

In conclusion, current studies have successfully highlighted the critical role of SIRT1 in urogenital and genital system diseases. However, at the molecular level, previous studies did not concentrate on certain pathways; thus, the mechanism of the effect of SIRT1 was inconsistent between different studies. Furthermore, although other SIRT proteins have not been as extensively studied as SIRT1, the important effect of these proteins in urogenital disease should not be ignored. The association between the SIRT protein family and urogenital disease could still be a new direction for further research.

#### SIRTs and motor system diseases

Diseases of the motor system focus on abnormal bone metabolism and diseases resulting from skeletal muscle dysfunction, mainly including OA, osteoporosis, intervertebral disc degeneration (IDD) and skeletal muscle atrophy. In addition to the body’s own self-regulatory mechanisms, exogenous factors such as aging, mechanical stimulation, estrogen, and obesity are involved in the process of bone metabolism and skeletal muscle function.^928^ SIRTs are considered promising regulatory genes for bone and skeletal muscle metabolism, involved in processes such as differentiation of bone marrow MSCs, osteoblast viability, skeletal muscle fiber type conversion, endoplasmic reticulum stress and atrophy.^929^ Therefore, in this section, we focus on the functions of SIRTs in diseases of the locomotor system and the regulatory roles.

##### OA

OA is the most common joint disease, and is a type of degenerative disease.^930^ Chondrocyte senescence and apoptosis, ECM degradation with synovial inflammation, and dysfunction of the subchondral bone are the core pathological changes in OA.^931^ SIRTs may have different roles in influencing chondrocyte activity. Notably, SIRT1 is the best studied SIRT in OA, and negatively regulates important cellular biological processes impairing chondrocytes activity, including apoptosis and ECM degradation. For example, SIRT1 may reduce apoptosis and ECM degradation in OA chondrocytes via the Wnt/β-catenin signaling pathway to counteract aging-induced OA.^932^ Furthermore, SIRT1 is regulated by the circ0001103/miR-375 axis, which attenuates IL-1β-induced chondrocyte apoptosis and ECM degradation.^933^ In addition, SIRT1 can influence mitochondrial function, defense oxidative stress and inhibit senescence of chondrocytes. SIRT1 can reverse homocysteine-induced deleterious changes in chondrocytes that lead to OA via the SIRT1/PGC-1α/PPAR-γ cascade, including mitochondrial dysfunction and accumulation of oxidative stress.^934,935^ SIRT1 also improves the resistance of cartilage to oxidative stress by inhibiting epidermal growth factor receptor ubiquitination, thereby alleviating OA.^936^ Moreover, although there are many mechanisms affecting cellular senescence, SIRT1 can inhibit chondrocyte senescence and OA by negatively regulating the Wnt/β-catenin signaling pathway.^937^

SIRT2, SIRT3, and SIRT6 are also involved in the development of OA. Similar to SIRT1, SIRT2 and SIRT6 also play protective roles in disease progression. For instance, SIRT2 protects against the progression of OA by inhibiting degradation of the ECM by preventing the acetylation of p65.^89^ Moreover, SIRT6 can inhibit the senescence of chondrocytes by negatively regulating the NF-κB-mediated inflammatory response.^81^ However, SIRT3 was shown to have dual roles in disease progression. In detail, SIRT3 inhibited chondrocyte degeneration by maintaining mitochondrial homeostasis.^938^ SIRT3 alleviated OA by improving the resistance of cartilage to oxidative stress. Mechanistically, SIRT3 restored acetylation-dependent SOD2 activity in human OA cartilage.^939^ On the contrary, SIRT3 overexpression promoted OA chondrocyte apoptosis and reduced cell proliferation, finally resulting in OA progression.^940^ The molecular mechanism of the opposite effect of SIRT3 on OA is unclear, and is worth further study.

Overall, SIRT1-3, and SIRT6 have different effects on the viability and function of chondrocytes, and they play important roles in the occurrence and development of OA. Given that their complex mechanisms are not fully understood, more in-depth studies are needed on the interaction of SIRTs with cartilage, synovium, bones and joints. In particular, SIRT1 has an important role in the development of OA and is expected to be a therapeutic target for the treatment of OA in the future.

##### Osteoporosis

Osteoporosis is defined as a systemic skeletal disease characterized by low bone mass and deterioration of bone tissue microarchitecture, which increases bone fragility and fracture susceptibility.^941^ Bone exhibits continuous self-renewal, with replacement of old bone by new bone through osteoclast-mediated bone resorption and osteoblast-mediated bone formation, thereby repairing microstructural damage to bone, a process called bone reconstruction.^942^ In dynamic bone reconstruction, SIRTs not only promote osteoblast differentiation and inhibit osteoclast differentiation, but also inhibit osteoclast bone resorption, ensuring a positive balance between bone metabolism and increased bone mass through multiple pathways.^943^ SIRT1 KO mice have a low bone mass phenotype.^944^ Therefore, due to the confluence of cellular aging, energy metabolism and bone metabolism, SIRTs are of great significance in the study of osteoporosis pathogenesis.

Oxidative stress and aging are important factors that regulate the osteogenic differentiation process, and these can also be regulated by SIRT1 and thus are anti-osteoporosis.^945^ For instance, SIRT1 overexpression increased osteoblast osteogenesis through FoxO3a deacetylation and oxidative stress inhibition.^946^ Overexpression of SIRT1 might also reduce oxidative stress through the FoxO1 and β-catenin signaling pathways.^222^ In addition, SIRT1 plays a protective role in osteoporosis by regulating bone metabolism. For example, SIRT1 is regulated by the HIF-1α signaling pathway, which deacetylates sclerostin and activates the Wnt/β-catenin signaling pathway, leading to increased bone anabolism in osteoporosis.^947^ In postmenopausal osteoporosis, SIRT6 has been found to inhibit age-related bone loss by stabilizing ER alpha in preosteoblastic cells.^948^ Moreover, SIRTs not only regulate oxidative stress and aging signaling pathways to resist osteoporosis, but can also be activated by small molecule drugs such as resveratrol to affect bone metabolism. In osteoporosis, SIRT1 is activated by resveratrol and subsequently restores the levels of serum markers alkaline phosphatase and osteocalcin by inhibiting the NF-κB signaling pathway, which has a protective effect against osteoporosis.^949,950^

In brief, SIRT1 and SIRT6 can inhibit the development and progression of osteoporosis by resisting oxidative stress, aging and regulating bone metabolism. However, more in-depth and detailed studies are still needed to elucidate the regulatory mechanisms of other SIRTs on osteoporosis and to explore their clinical application value in the future.

##### IDD

IDD is an important pathological basis for degenerative spinal diseases, which manifests as increased degradation of the central nucleus pulposus matrix, thickening of the peripheral annulus fibrosus, and thinning and calcification of the cartilage endplates.^951^ SIRTs can inhibit the pathological process of IDD by inhibiting inflammation, cellular senescence, oxidative stress, and maintaining mitochondrial function. mRNA and protein expression levels of SIRT1 in degenerative nucleus pulposus tissues of intervertebral discs were reduced compared with control tissues and decreased with increasing disease severity.^952^ This suggests that there might be a protective effect of SIRT1 on IDD progression. Mechanistically, SIRT1 can resist the inflammatory response during IDD by inhibiting the transcriptional activity of NF-κB.^58^ Moreover, SIRT1 might inhibit disc degeneration by suppressing phosphorylation of activin 1 subunits c-Fos and c-Jun.^953^ It seems that SIRT1 might become a biological target for the treatment of IDD.

Furthermore, SIRT2, SIRT3, and SIRT6 have protective roles in the development of IDD. For example, SIRT2 reversed the action of IL-1β by inhibiting the p53/p21 pathway, inhibited oxidative stress and cellular senescence, and thus prevented the degradation of nuclear myeloid cells in IDD.^954^ It was also found that SIRT3 maintains nucleus pulposus cell homeostasis to prevent IDD mainly by regulating mitochondrial oxidative stress levels.^955,956^ In addition, SIRT6 mainly inhibits the inflammatory response and cellular senescence during IDD by inhibiting the transcriptional activity of NF-κB pathways.^957^

In conclusion, SIRTs, including SIRT1-3, and SIRT6, are involved in negative regulation of disease progression in IDD; however, the number of studies are limited. With the continuous discovery of acting molecules and the identification of deep molecular mechanisms, SIRTs are expected to become important targets in the prevention and treatment of IDD.

##### Skeletal muscle atrophy

Skeletal muscle atrophy, which is the accelerated degradation of skeletal muscle proteins, mainly involves a variety of chronic diseases, aging, and long-term muscle inactivity.^958^ SIRTs inhibit skeletal muscle atrophy and are associated with mechanisms such as mitochondrial dysfunction, autophagy and metabolism. For example, SIRT1 inhibited drug-induced mitochondrial dysfunction and thus alleviated skeletal muscle atrophy by activating its downstream signaling PGC-1α.^959^ Moreover, SIRT2 effectively inhibited the autophagic flux, thus maintaining protein metabolism homeostasis in skeletal muscle.^960^ SIRT3-mediated cellular metabolism has an inhibitory effect in skeletal muscle atrophy. Ang II caused skeletal muscle atrophy, and SIRT3 deficiency enhanced Ang II-induced fiber type transformation and mitochondrial metabolic reprogramming, exacerbating skeletal muscle atrophy.^961^ The incidence of skeletal muscle atrophy and sarcopenia is increasing year by year.^962^ As the research on SIRTs in skeletal muscle physiological and pathological processes continues to advance, SIRTs could be used as targets for the prevention and treatment of skeletal muscle-related diseases.

##### Conclusion

SIRTs are key nodes in several degenerative diseases of aging, including OA, osteoporosis, and IDD. SIRTs play a key role in bone homeostasis and can maintain the balance between bone formation and resorption by regulating the ratio of osteoblasts to osteoclasts. SIRTs enhance the viability of osteoblasts under unfavorable conditions by resisting senescence, inhibiting apoptosis and promoting autophagy. Therefore, given the critical role of the SIRTs pathway in bone homeostasis, it is likely to be a potential therapeutic target, laying a solid foundation for further studies in the future.

#### SIRTs and aging

Aging is associated with impaired adaptive and homeostatic mechanisms, leading to susceptibility to environmental or internal stresses with degeneration of multiple organ systems.^963^ Extensive studies have clearly revealed that SIRTs are important regulators of aging, which involves several biological processes, such as cellular senescence, metabolic regulation, genome fidelity, nutrient sensing, and circadian rhythms.^964^

It has long been known that mammalian aging is associated with cellular senescence, and SIRTs could play key roles in antagonism of aging and cellular senescence.^965^ For example, the activation of SIRT1 by La Ribonucleoprotein 7, a 7SK RNA binding protein, could ameliorate cellular senescence and aging through dampening p53 and NF-κB (p65) transcriptional activity.^966^ Besides, SIRT6 inhibition shortened human VSMC lifespan and induced senescence, associated with telomeric histone H3K9 hyperacetylation and p53 binding protein 1 binding, while SIRT6 overexpression preserved telomere integrity, delayed cellular senescence.^201^ Additionally, SIRTs could exert regulatory effects on aging by regulating cellular homeostasis in fundamental pathways such as genomic stability, nutrient sensing, and protein homeostasis.^964^ For instance, SIRT1 redistribution on chromatin induced by DNA damage, could promote DNA repair, enhance genomic stability, and suppress age-dependent transcriptional changes.^967^ SIRT3 deficiency resulted in the detachment of genomic lamina-associated domains from the nuclear lamina, increased chromatin accessibility and aberrant repetitive sequence transcription, and thereby leading to senescence phenotypes of human mesenchymal stem cells.^968^ Moreover, SIRT6 promoted resistance to DNA damage, suppressed genomic instability in mouse cells via deacetylation of Polβ, a base excision repair factor, and prevent the development of several progeroid pathologies.^35^ In addition to genomic stability, nutrient sensing is also known to be an important factor affecting aging.^969^ Evidence suggested that SIRT1 could control the gluconeogenic/glycolytic pathways in liver in response to fasting signals and modulate aging.^970^ The SIRTs-related roles in aging are also associated with the regulation of protein homeostasis. The related studies showed that the SIRT-activating compounds-resveratrol could attenuate copper-induced senescence by improving cellular proteostasis.^971^ Furthermore, the involvement of SIRTs in aging could be mediated by their roles in circadian rhythms.^972^ For instance, SIRT1 constituted a reciprocal negative regulation loop with the periodic gene, Period 2, and thus modulated circadian-clock maintenance and aging gene expression.^973^ SIRT6 deacetylated Period 2 and thus regulated circadian rhythms which were associated with aging and age-related diseases.^974^

Therefore, SIRTs have very important regulatory roles in aging through participating in diverse biological processes. Moreover, growing evidence has shown that SIRTs might be attractive anti-aging molecules involved in improving health, although it is still under debate and has not been fully defined.^975^ From this perspective, further studies are needed to uncover the exact roles and mechanisms of SIRTs in the aging process.

### SIRT modulators

In view of the dual involvement of SIRTs in many biological processes, many laboratories have developed both SIRT inhibitors and activators, which might act as tools for studying SIRT function and potentially as treatments for different conditions and diseases. Generally, activators have better therapeutic potential than inhibitors. This might partly be attributed to higher target specificity in the enzyme family and fewer side effects.^976^ However, compared to inhibitors, the number of activators is small. The following sections describe in detail the most relevant SIRT inhibitors and activators identified so far.

#### SIRT activators

**Fig. 16:**
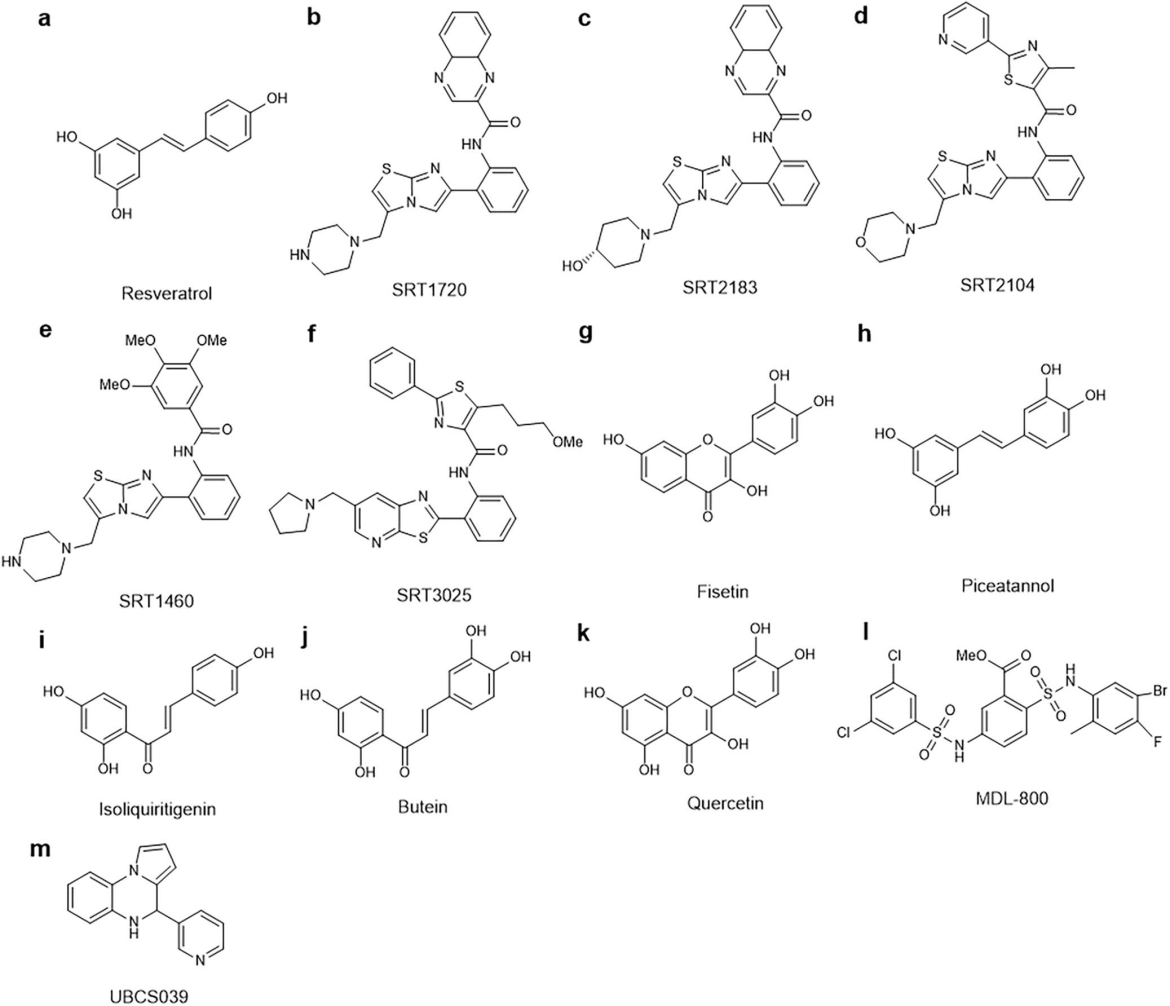
Structures of most relevant SIRT activators

SIRT activators are mainly classified into natural polyphenols and nonrelated synthetic SIRT activators (Supplementary Table 1). The structures of these activators are shown in Fig. 16. Among them, resveratrol, a polyphenol commonly found in grapes and red wine, was the first SIRT1 activator identified in 2003.^977^ Resveratrol as an allosteric activator of SIRT1 can increase its activity by 50% (EC_1.5_) at 46.2 μM and extend the lifespan of many organisms, ranging from yeast to mammals.^977^ Evidence showed that resveratrol supplementation could help to reduce fasting glucose, insulin, and insulin resistance, increase high-density lipoprotein-cholesterol levels and total antioxidant capacity, and upregulate PPAR-γ and SIRT1 in the peripheral blood mononuclear cells of type 2 DM patients with coronary heart disease.^978^ Several other polyphenols, structurally related to resveratrol, were also found to activate SIRTs, including the chalcones butein and isoliquiritigenin, the flavones fisetin and quercetin, and the stilbene piceatannol.^977^ The compounds fistein and butein increased lifespan length in the yeast *Saccharomyces cerevisiae* by 33% and 5%, respectively,^977^ whereas quercetin increased the lifespan of the nematode *Caenorhabditis elegans* grown in 200 mM by approximately 20%.^979^ In addition, butein was reported to attenuate sepsis-induced brain injury through alleviation of cerebral inflammation, oxidative stress and apoptosis by SIRT1 signaling activation.^980^ Although piceatannol and isoliquiritigenin did not produce significant effects on lifespan, they were believed to activate prolonged survival.^981^ Isoliquiritigenin repressed the proliferation, migration, and invasion of NSCLC cells in vitro.^825^ Furthermore, in experimental diabetic neuropathy, isoliquiritigenin could reduce oxidative damage and alleviate mitochondrial impairment by SIRT1 activation.^982^

As natural compounds did not show high activity on SIRT1, more potent compounds with a greater substrate-binding affinity for SIRT1 have been synthesized. SRT compounds, such as SRT1460 (EC1.5 = 2.9 μM),^983^ SRT1720 (EC1.5 = 0.16 μM),^983^ SRT2104 (EC1.5 = 0.43 μM),^984^ SRT2183 (EC1.5 = 0.36 μM),^983^ and SRT3025 (EC1.5 < 1 μM),^985,986^ were identified in 2007 as selective SIRT1 activators, which were more potent than resveratrol.^983^ They played important roles in the treatment of physiological and pathological conditions.^983,987,988^ For example, employment of SRT compounds in diet-induced and genetically obese mice improved insulin sensitivity and glucose tolerance, stimulated mitochondrial biogenesis, and regulated lipid metabolism, thus had beneficial effects on weight loss.^983^ Due to these promising activities, some SRT compounds have been evaluated in various clinical trials for the treatment of different conditions and diseases.^989^ SRT2104 was the most common intervention for healthy participants and type 2 diabetes patients in randomized controlled trials (RCTs).^8,990,991^

The development of SIRT6 activators was initially stimulated by early studies showing that free fatty acids containing 14–18 carbons acted as weak SIRT6 activators.^992^ UBCS039 is a pyrrolo[1,2-a]quinoxaline reported as the first synthetic activator of SIRT6 deacetylase activity (EC_50_ = 38 μM).^993^ Evidence shows that UBCS039 induced a time-dependent activation of autophagy and induced deacetylation of SIRT6-targeted histone in several human tumor cell lines.^994^ The bis benzenesulfonamide-based prodrug MDL-800 is also reported to be a potent and selective SIRT6 activator with an EC_50_ value of 10.3 μM.^995^ MDL-800 decreased both H3K9ac and H3K56ac at a concentration of 10 µM and showed a dose-dependent effect in Bel7405, PLC/PRF/5, and Bel7402 cell lines at 24 h and 48 h.^995^ Additionally, MDL-800 decreased TGF-β1-induced activation of myofibroblast and ECM production by regulating SIRT6-dependent β-catenin acetylation and the TGF-β1/Smad signaling pathway.^907^

#### SIRT inhibitors

**Fig. 17:**
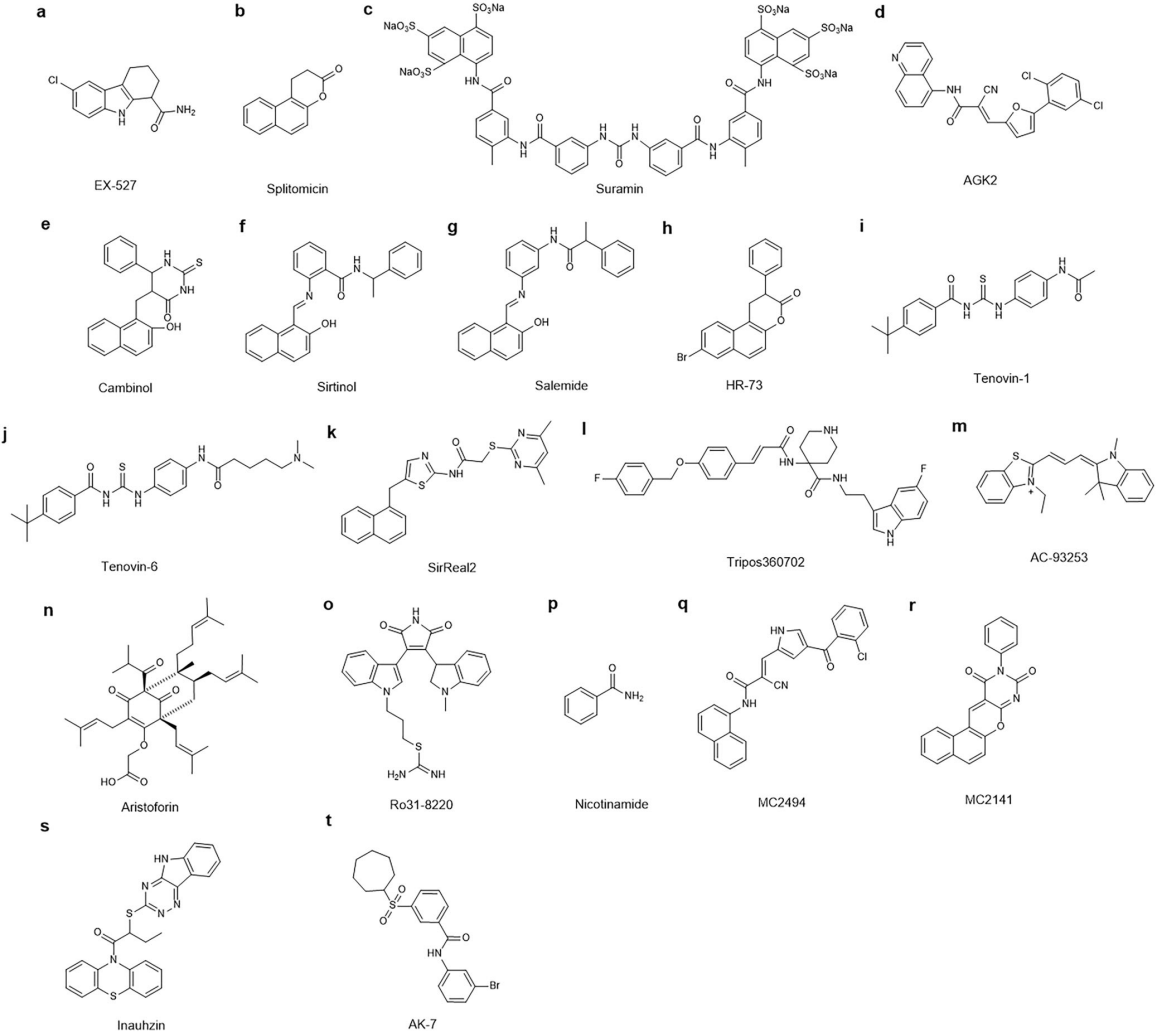
Structures of most relevant SIRT inhibitors

Compared to SIRT activators, more studies have been conducted on SIRT inhibitors. A range of potent inhibitors were identified through a variety of development strategies, such as mechanism/structure based, or simply by virtual screening.^976^ Most studies focused on the inhibition of human SIRT1 and/or SIRT2. These available inhibitors are divided into several structural groups based on their mechanism of action and structural features (Supplementary Table 2). Figure 17 shows the structures of these inhibitors.

##### Nicotinamide and its analogs

Nicotinamide riboside (NR) and nicotinamide mononucleotide (NMN) are important precursors of NAD, in that NAD biosynthesis involves the conversion of nicotinamide to NMN and subsequent conversion of NMN to NAD.^996^ And the production of NMN is the key rate-limiting factor in mammalian NAD biosynthesis.^996^ Thus, NR and NMN might affect SIRT activity mainly by affecting the synthesis of NAD. Nicotinamide and its analog, AK-7, are reported to be SIRT inhibitors. Of these, nicotinamide is the endogenous inhibitor of SIRTs, which is formed from NAD + during catalysis. Nicotinamide inhibits SIRT1 and SIRT2 with IC_50_ values of approximately 120 μM and 100 μM for SIRT1 and SIRT2, respectively.^997,998^ Nicotinamide can inhibit the growth and viability of human prostate cancer cells through inhibition of SIRT1.^999^ In addition, it blocks proliferation and induces apoptosis of chronic lymphocytic leukemia cells.^1000^ AK-7, a benzamide (a nicotinamide mimic)-containing compound, shows selective SIRT2 inhibition.^1001^ An in vivo study showed that AK-7 improved behavioral and neuropathological phenotypes, prolonged survival, and improved HD neuropathology in R6/2 HD mice.^1002^ Furthermore, AK-7 limited the ability of adoptively transferred antigen-specific CD4 + T cells to cause autoimmune encephalomyelitis in mice and limited disease in lupus-prone MRL/lpr mice.^1003^ This might support the development of SIRT2 inhibitors as potential therapeutics for these diseases.

##### β-naphthol-containing inhibitors

β-naphthol acts as a key group for several SIRT inhibitors, including splitomicin, sirtinol, salermide, HR-73, and cambinol. Both sirtinol and splitomicin were identified through cell-based screens of more than 1000 compounds in yeast.^1004,1005^ Splitomicin inhibits human SIRT1 and SIRT2 with an IC_50_ value of 96 μM and 113 μM in vitro, respectively.^9^ Treatment with this molecule reduces deacetylase activity, enhances tissue factor mRNA expression in stimulated endothelial cells, and enhances NF-kB/p65 nuclear translocation.^1006^ In addition, evidence showed that splitomicin could reversed both ischemic preconditioning-mediated lysine deacetylation and ischemic preconditioning-induced cardioprotection.^1007^ Sirtinol (SIRT1 IC_50_ = 131 μM;^1008^ SIRT2 IC_50_ = 38-58 μM^1005,1008^) has been shown to induce apoptotic and autophagic cell death in MCF-7 human breast cancer cells.^1009^ Sirtinol induced senescence-like growth arrest in human LC H1299 cells and induced senescence-like growth arrest as well as apoptotic and autophagic cell death breast cancer MCF-7 cells.^1009,1010^ Structure-activity relationship studies on sirtinol resulted in improved analogs such as salermide, which has a greater inhibitory effect on SIRT1 and SIRT2 than sirtinol.^1011^ Salermide was reported to induce the reactivation of proapoptotic genes that were aberrantly repressed in cancer cells by SIRT1-mediated H4K16 deacetylation.^1011^ Also, salermide had potent antiproliferative on human leukemia MOLT4 cell lines, human breast MDA-MB-231, and colon RKO cancer cell lines and played an inhibitory role in colorectal carcinoma cancer stem cells.^1012^ HR-73 was identified as a splitomicin derivative, which inhibits the activity of SIRT1 in vitro with an IC_50_ lower than 5 μM.^1013^ It can decrease human immunodeficiency virus transcription through Tat acetylation.^1013^ Cambinol, a β-naphthol derivative, is the most promising SIRT inhibitor in this class of compounds.^1014^ It inhibits human SIRT1 and SIRT2 with IC_50_ values of 56 and 59 μM in vitro, respectively,^240^ and reduces the expression of poorly differentiated markers α-fetoprotein and glypican, and impairs cell migration in a dose-dependent manner.^1015^ It was also reported to reduce the expression of N-Myc protein and up-regulate the expression of the other SIRT1 target genes including early growth response 1, Kv channel interacting protein 4, and phospholipase C beta 1.^1016^

##### Indole derivatives

Large-scale fluorescence screening led to the emergence of pure indole Sir2 inhibitors in 2005, which are the only series of compounds with a simple indole as the scaffold identified to date.^1017^ These compounds include EX-527, AC-93253, inauhzin, and Ro31–8220. They act as inhibitors of SIRT1, which enhance cell survival and p53 acetylation.^1018^ EX-527, also called Selisistat, is the first known selective (over SIRT2/3) and cell-permeable SIRT1 (IC_50_ = 0.098 μM) inhibitor.^1019^ Evidence showed that EX-527 decreased tumor growth in endometrial and LC cells xenografted mice.^469,1020^ Additionally, EX-527 could decrease the viability of control HHUA cells and the survival of HEC151 cells and reduce cisplatin resistance in HEC1B cells with mutated and non-functional p53.^469^ AC-93253, a compound containing a modified indole ring, preferentially inhibits SIRT2 (IC_50_ = 6 μM)^1021^ and triggers the downregulation of melanoma progression markers and the inhibition of melanoma cell proliferation.^1022^ Another indole derivative, inauhzin, inhibits the deacetylase activity of SIRT1 with an IC_50_ value of 0.7-2 μM.^1023^ Inauhzin has potent anticancer activity and represses the growth of xenograft tumors derived from human LC H460 and colon cancer HCT116 cells harboring p53.^1023^ Also, inauhzin was found to induce ribosomal stress and the RPL11/RPL5- murine double minute 2 (MDM2) interaction, activating p53, and suppress cancer cell growth by dually targeting SIRT1 and inosine monophosphate dehydrogenase 2.^1024^ SIRTs use NAD as a co-substrate, whereas kinases use ATP as a co-substrate. Given that both NAD and ATP contain an adenosine moiety, kinase inhibitors might inhibit SIRTs. For example, a nM PKC inhibitor, Ro31–8220, shows inhibitory activity against SIRT1 and SIRT2, with IC_50_ values of 3.5 μM against SIRT1 and 0.8 μM against SIRT2.^1025^ In a human neuroblastoma cell line, Ro31–8220 was found to reduce PKC activity and the tau phosphorylation pattern.^1026^

##### SIRT-rearranging ligands (SirReals)

A family of aminothiazoles, basically known as SirReals, was found to act as a potent selective inhibitor of SIRT2. Of these, SirReal2 is a potent SIRT2 inhibitor (IC_50_ = 0.14 μM) with minimal effects on SIRT1 and SIRT3.^1027^ According to X-ray crystallography, SirReal2 exerts its potency and selectivity based on a ligand-induced structural rearrangement of the SIRT2 active site and interacts with residues in an unknown binding pocket located near the zinc-binding domain, known as the “selective pocket”.^1027^ The SIRT2 inhibition capability of SirReal2 has been confirmed in chondrocytes by the induction of several acetylations of H3.^1028^ SirReal2 was reported to increase the levels of phosphorylated Cx43 on S368 and the levels of acetylated MEK1/2, decrease the membrane localization of Cx43 between cumulus cells, and increase the Cx43 acetylation levels of cumulus-oocyte complexes.^1029^

##### Tenovins

Through phenotypic screening of 30,000 drug-like small molecules able to activate p53 and decrease tumor growth, Lain et al. discovered two compounds that were SIRT1 inhibitors: tenovin-1 and its more water soluble analog tenovin-6.^1030^ The poor water solubility of tenovin-1 prevents the accurate determination of an IC_50_ value, whereas IC_50_ values of tenovin-6 with better water solubility have been reported as follows: SIRT1 IC_50_ = 21 μM; SIRT2 IC_50_ = 10 μM;^1030^ SIRT3 IC_50_ = 67 μM.^1030^ Both compounds decrease tumor growth in vitro at one-digit micromolar concentrations, and delay tumor growth in vivo without significant general toxicity.^1030^ In the BL2 and ARN8 mouse xenograft model, tenovin-1 could reduce tumor growth,^1030^ while tenovin-6 was found to delay the growth of xenograft tumors derived from ARN8 cells.^1030^

##### Other SIRT inhibitors

Many other types of compounds have been reported as SIRT inhibitors. Some of them are worth mentioning. Suramin, a polyanionic urea derivative, was originally used as an adenosine receptor antagonist for the treatment of trypanosomiasis and has antiviral and anticancer activity.^1031^ It was later found to be a potent SIRT inhibitor with an IC_50_ of 297 nM, 1150 nM, and 22 μM for SIRT1, SIRT2, and SIRT5, respectively.^1032,1033^ Suramin has multiple biological effects, such as protection against disc degeneration, perturbation of mitochondrial membrane potential and ATP levels.^10,1034^ Aristoforin, a phloroglucinol derivative, was shown to inhibit SIRT1 (IC_50_ = 7 μM) and SIRT2 (IC_50_ = 21 μM).^1035^ AGK2 is a selective SIRT2 inhibitor (IC_50_ = 3.5 μM) identified from a concentrated compound library.^1036^ The design of MC2494 is inspired by AGK2, in which the 2,5-dichlorophenyl-substituted furan ring is replaced by a pyrrole bearing a 2-chlorobenzoyl moiety at the C4 position. MC2494 has been reported as a micromolar pan-SIRT inhibitor and regulated mitochondrial function in a leukemia cell line.^1037^ As the result of cambinol manipulation, MC2141 was identified in 2010 and was the prototype of a class of benzodeazaoxaflavins that inhibited SIRT1/2 in the low micromolar range.^1038^ Tripos 360702 showed SIRT2 inhibitory activity with IC_50_ values of 51 μM in a test, and could be considered a novel inhibitor of SIRT2.^1039^

#### Conclusion

After summarizing the activators and inhibitors of the SIRT protein family described above, we find that a substantial amount of progress has been made in past decades. However, as research on different types of SIRT modulators has been unbalanced and the clinical potential of these modulators in treating different diseases has been insufficient, there is still progress to be made. Currently, inhibitors of SIRT1/2 are relatively adequate, whereas there are no good inhibitors of SIRT3-7 to date, especially SIRT4 and SIRT7. Additionally, with regard to SIRT activators, a great deal of work has been conducted in the identification of molecules targeting SIRT1. Thus, further studies are needed to investigate activators and inhibitors of other SIRTs rather than SIRT1, which will eventually unlock the full therapeutic potential of SIRT molecules. We believe that SIRT modulators are a field worthy of research, the SIRT protein family will eventually pay off.

### RCTs of SIRT proteins

**Fig. 18:**
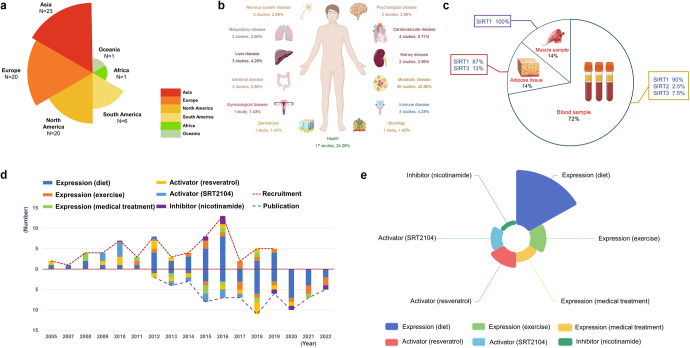
Characteristics of included randomized controlled trials by (**a**) regions; (**b**) condition of subject; (**c**) examination of tissue and samples; (**d**) years of recruitment and publication; (**e**) interventions

We conducted an electronic search of relevant RCTs in PubMed (up to June 23, 2022) without restrictions. Additionally, relevant clinical trial registration sites were comprehensively examined, such as ClinicalTrials.gov, ISRCTN registry, EU Clinical Trials Register, and Iranian Registry of Clinical Trials. Literature retrieval was performed in duplicate by two independent reviewers. A total of 63 published RCTs were included, of which 43 studies mainly examined the effects of different interventions on SIRT protein expression in human samples, and 20 studies focused on the impact of SIRT activators (resveratrol and SRT2104) and SIRT inhibitor (nicotinamide) on physiological function in different participants. The characteristics of these studies are shown in Fig. 18 and listed in detail in Tables 1–2.

**Table 1:** Summary of published clinical trials on the expression of Sirtuin

Trial [ref]	Year	Phase	Participant	Intervention/ Comparison	Sample size of intervention/ comparison	Outcome	Main findings
NCT02258438^1065^	2022	N/A	Overweight or obese adults	• MICRO (5 min brisk walking each hour for 9 h) • ONE (45 min/d continuous brisk walking bout) • SED (sedentary CON)	20 (cross-over)	Skeletal muscle mitochondrial respiration and molecular adaptations	ONE and MICRO enhanced SIRT signaling expression
IRCT201406183664N12^1066^	2022	N/A	NAFLD patients	• Turmeric powder (six 500-mg capsules) • Placebo (six placebo capsules, for 12 weeks)	23/23	Blood pressure and serum levels of SIRT1 and adiponectin	Turmeric effectively improved SIRT1 levels in patients with NAFLD
NCT01003392^1067^	2022	N/A	Healthy adults	• Pure Arabica coffee • Blended (Arabica + Robusta) coffee (450 to 600 mL/day for 8 weeks)	20/33	Blood SIRT1, lipids, and homocysteine	Both Arabica and blended coffees increased serum SIRT1 concentration
U1111-1237-8231^1068^	2022	N/A	CKD patients	• RT (3 times per week) • RT with BFR (3 times per week, for 6 months) • CON group	35/35/35	Kidney function	SIRT1 increased in the RT and RT + BFR groups
NCT02480504^1069^	2021	N/A	Abdominally obese subjects	• Intermittent CR (consumed 400/600 kcal (female/male) on two non-consecutive days with normal energy intake rest of the week) • Continuous CR (reduced their energy intake evenly for seven days, for one year)	48/54	SIRT1 concentrations	Effects on SIRT1 concentrations after 1 year of CR are sex and BMI-related. Intermittent CR regimen affected SIRT1 to a stronger extent than continuous CR
CTRI/2017/05/008589^1046^	2021	N/A	RA	• Yoga practice (five times a week for 120 min duration per session) • Non-yoga group (maintain their normal day to day physical activities with no change, for 8 weeks)	35/35	Changes in disease activity and functional status	The mRNA expression levels of SIRT1 were not found to be different statistically in the yoga vs. non-yoga group
DRKS00014322^1070^	2021	N/A	Healthy elderly participants	• Exercise (a warm-up and two passes of a strength endurance circuit for 12 weeks) • Exercise and dietary counseling (dietary counseling prior to initiation of the exercise program) • Exercise and CO supplementation (maintain their habitual diet supplemented with capsules providing 2.0 g of CO per day) • CON	14/8/9/9	SIRT activities (SIRT1, SIRT3, and SIRT5 in blood)	The activity of SIRT1 and SIRT3 increased in response to the exercise intervention
CAAE: 73,585,317.0.0000.5440^1050^	2021	N/A	Non-diabetic obese women	• RYGB (three analyzes were carried out, one day before surgery and 3 and 6 months after the surgical procedure)	13	ER‑stress and inflammation on subcutaneous adipose tissue	At 3 and 6 months after RYGB, the expression of SIRT1 and SIRT3 increased compared to the baseline. After 3 and 6 months, the expression of SIRT1 was positively correlated with the BMI changes in the same period.
IRCT20090822002365N23^1071^	2021	N/A	Obese women with mild to moderate depressive symptoms	• Co- supplementation group (receiving a 50,000 IU VD soft gel weekly + a 250-mg magnesium tablet daily) • VD (receiving a 50,000 IU VD soft gel weekly + a magnesium placebo daily) • Magnesium group (receiving a VD placebo weekly + a 250-mg magnesium tablet daily) • CON (receiving a VD placebo weekly + a magnesium placebo daily, for 8 weeks)	25/26/26/25	Anthropometric indices, depressive symptoms, serum levels of BDNF, inflammation, and SIRT1	SIRT1 increased significantly in the 3 intervention groups. VD plus magnesium supplementation has beneficial influences on SIRT1
IRCT20141025019669N13^1072^	2021	N/A	T2D patients	• Oral ellagic acid (180 mg once daily) • Placebo (a capsule containing wheat flour once daily, for 8 weeks)	21/21	IR and Fetuin-A and serum SIRT1	Ellagic acid supplementation significantly increased SIRT1 levels compared with the placebo group
IRCT201604202365N11^1073^	2020	N/A	Obese T2D patients	• VD (50,000 IU/week) • Placebo (50,000 IU/week, for 8 weeks)	42/43	Serum 25-OH VD, SIRT1, Irisin, HbA1c, IR indexes, fasting blood sugar, and serum insulin	The increase of serum SIRT1 in the intervention group was significant. VD supplementation may improve T2D by decreasing HbA1c and increasing SIRT1 and irisin in VD deficient T2D patients
IRCT20091114002709N50^1042^	2020	N/A	Overweight or obese patients with PCOS	• Curcumin (500 mg three times daily) • Placebo (1500 mg/day of maltodextrin, for 12 weeks)	34/33	Oxidative stress enzymes, SIRT1 gene expression	Curcumin non-significantly increased gene expression of SIRT1
IRCT2016061128392N1^1074^	2020	N/A	T2D patients	• Cinnamon (three capsules of 1 g cinnamon extract daily) • Placebo (microcrystalline cellulose daily, for 8 weeks)	20/19	Expression of systemic inflammation factors, NF-κB, and SIRT1	Cinnamon supplementation has no beneficial effects in reduction of SIRT1 levels in T2D patients
IRCT201512102017N26^1040^	2020	N/A	CAD	• Crocin (30 mg/d) • Saffron aqueous extract (30 mg/d) • Placebo (for 8 weeks)	22/23/20	Gene expression of SIRT1, 5'- AMPK, LOX1, NF-κB, and MCP-1	Crocin may have beneficial effects on CAD patients by increasing the gene expression of SIRT1 and AMPK.
IRCT2015080823559N1^1075^	2020	N/A	COPD	• CLA (a soft gel capsule contains CLA 3.2 g daily) • Placebo (the same amount soft gel capsule, for 6 weeks)	40/42	Forced expiratory volume in one second, BODE index, serum levels of IL-6 and SIRT1	Serum levels of SIRT1 significantly increased in the supplementation group
IRCT20131117015424N2^1076^	2020	N/A	Obese adults	• α-LA (2 capsules containing 600 mg α-LA 1 h before lunch and dinner along with a CR diet) • Faradic (performed Faradic exercise for 8 weeks and 3 sessions of 60 min per week) • α-LA + faradic (received both 2 capsules daily containing 600 mg and Faradic exercise along with a weight loss regimen) • CON (receive 2 placebo capsules containing 600 mg wheat flour with CR diet)	25/25/25/25	Anthropometric measurements, serum levels of vascular endothelial growth factor, NO, SIRT1, and PGC1-α	SIRT increased significantly in the α-LA + Faradic group compared to the control group. It is possible that α-LA and Faradic have synergic anti-obesity effects and exert their effects through increasing the serum level of SIRT and PGC
IRCT20091114002709N51^1077^	2020	N/A	UC	• Selenomethionine (200 g/day) • Placebo (capsules contained rice flour, for 10 weeks)	50/50	Expression of SIRT1 and PGC-1α genes	Selenium supplementation caused a significant decrease in the inflammatory response of the colon by a significant increase in the expression of the SIRT1 gene
REF/2016/01/010500^1043^	2020	N/A	RA	• YBLI (yogic practices for five times a week for 120 min duration per session) • Non-yoga group (follow normal day to day physical activities with no change in the routine, for 8 weeks)	33/33	Changes in disease activity; The levels of psycho-neuro-immune axis markers and the expression patterns of following genes: IL-6, TNF-α, NF-κB 1, TGF-β, and CTLA4; Change in QOL of RA patients.	A marked improvement in the mind-body communicative markers was seen, which is indicated by increased levels of BDNF, DHEAS, β endorphins, and SIRT1, followed by 8 weeks of YBLI in RA patients
IRCT2016042717254N5^1078^	2019	N/A	Overweight or obese T2D patients	• GC (3 g of GC powder per day) • Placebo (3 g of rusk powder per day, for 10 weeks)	41/42	Physical activity level, dietary intake, anthropometric measurements, glycemic indices, blood lipids, and SIRT1 levels	GC can decrease HbA1c, insulin level, IR, and TG level via increase in SIRT1 concentration in T2D patients
IRCT201608223320N13^1079^	2019	N/A	Obese subjects with VD deficiency	• VD group (receiving weight loss diet + a bolus dose of 50000 IU cholecalciferol) • Placebo group (receiving weight loss diet + placebo pearls contained edible paraffin, every Friday right after lunch, for 12 weeks)	22/22	Changes in TC, LDL-C, TG and HDL-C and SIRT1	No significant effect of VD supplementation in combination of energy restriction on serum lipids profile and SIRT1 in obese subjects with VD deficiency was found
REF/2014/09/007532^1044^	2019	N/A	Parents of retinoblastoma patients	• YBLI program (five sessions per week for 12 weeks)	86	Psychological stress and QOL	YBLI led to a significant increase in the levels of SIRT1
IRCT20161102030649N1^1041^	2019	N/A	CAD	• Crocetin (one capsule of 10 mg per day) • Placebo (one capsule of placebo per day, for two months)	24/21	The atherogenesis related markers like SIRT1, LOX1, ICAM1, VCAM1, and MCP-1; the clinical outcomes, lipid profile, dietary intake, appetite, and h-FABP	The expression of SIRT1 gene statistically changed between the studied groups at the end of the trial. The relative increase in the gene expressions of SIRT1 in isolated PBMCs in the crocetin group were significant at the end of the trial in comparison with the placebo
NCT02886169^1080^	2018	N/A	Health employees	• HFM + SIO (100 g of buttered bread and sweetened coffee, also included 15 mL of commercial SIO) • HFM (100 g of buttered bread and sweetened coffee with a 2-week washout period)	42 (cross-over)	Carbohydrate metabolism and gene expression of SIRT1	A higher concentration of fasting triacylglycerides and SIRT1 expression at 4 hours post SIO. SIRT1 expression correlates with postprandial insulin sensitivity
NCT01754792^1081^	2018	N/A	Obese subjects	• PEB for 12 weeks	13	PBMCs, VAT and SAT	The consumption of a PEB increased SIRT1 protein expression in PBMCs
NCT03439592^1082^	2018	N/A	Obese patients with pre-diabetic condition/normo-glycemic condition	• A hypocaloric diet added to metformin therapy (850 mg twice a day) • A hypocaloric diet plus placebo	20/38	Inflammatory cytokines and SIRT1 levels in subcutaneous abdominal fat	An inverse correlation was found between subcutaneous fat expression of SIRT1 and myocardial performance index
IRCT2015121317254N4^1083^	2018	N/A	Obese patients with NAFLD	• GC (3 g/day) • Placebo (toast flour 3 g/day, for 3 months)	43/44	Serum SIRT1 and inflammation	GC supplementation could improve some biomarkers related to fatty liver including inflammation and SIRT1 in overweight/obese NAFLD patients
REF/2014/09/007532^1045^	2018	N/A	MDD patients	• YMLI (included sessions five days per week for 12 weeks) • CON	29/29	Changes in severity of MDD. Changes in the levels of blood biomarkers related to neuroplasticity	YMLI significantly increased SIRT1. Increased SIRT1 and telomerase activity and decreased cortisol significantly predicted this association
IRCT201206144010N8^1084^	2018	N/A	T2D patients	• PJ (250 mL daily) • Placebo (250 ml corresponding control beverages of similar color and energy content daily, for 12 weeks)	22/22	Plasma concentrations of soluble ICAM-1, soluble VCAM-1, and soluble E-selectin; NF-κB p65 and SIRT1 in the PBMC.	Compared with the placebo group, SIRT1 was significantly higher in the PJ group
NCT01714479^1085^	2017	N/A	Health adults	• LC + CHO + EAA (treadmill walking, wearing a vest equal to 30% of body mass + 46 g CHO and 10 g EEAs) • CE + CHO + EAA (CE + 46 g CHO and 10 g EAAs) • LC + CON (treadmill walking, wearing a vest equal to 30% of body mass + non-nutritive drinks) • CE + CON (CE + non-nutritive drinks)	6/7/5/7	Mitochondrial Biogenesis-Related Gene Expression	SIRT1 expression postexercise was higher for CON than for CHO + EAA treatments
NCT01939782^1086^	2017	N/A	Healthy volunteers and T2D patients	• Breakfast and lunch • Only lunch	32 (cross-over)	Clock gene expression and postprandial glycemia	In healthy individuals, the expression level of SIRT1 was lower after breakfast. In individuals with T2D, SIRT1 only slightly, but significantly, decreased after breakfast. In healthy individuals, the expression level of SIRT1 was higher after lunch on breakfast and lunch day
NCT02253732^1087^	2017	N/A	Early/mid-stage PD patients and sedentary seniors	• 3-month combined strength-endurance supervised exercise training program	11 (PD patients) + 11 (sedentary seniors)	REE, glucose metabolism, adiposity, and ^31^P- muscle energy metabolism	Improvements in fasting glycemia were positively associated with muscle function and the expression of SIRT1
NCT02261545^1088^	2017	N/A	T2D patients	• n-3 PUFA supplement (3 soft gels daily) • Placebo (3 soft gels daily, for 10 weeks)	44/41	Circulating level of adiponectin and mRNA expression of AdipoR1, AdipoR2 and SIRT1	No significant changes were observed for SIRT1 expression
NCT00429195^1089^	2016	N/A	MetS patients	• HSFA • HMUFA • LFHCC diets supplemented with long-chain n-3 PUFA (LFHCC supplemented with high-oleic sunflower seed oil capsules) for 12 weeks	17/18/20/20	Advanced glycation and product metabolism	Consumption of HMUFA and LFHCC n-3 diets produced higher SIRT1 mRNA levels compared to the other diets
NCT00692237^1047^	2016	N/A	T2D patients	• Sildenafil (100 mg/day) • Placebo (for 12 weeks)	29/25	Anthropometric and metabolic parameters	Up-regulation of SIRT1, a known target of miR-22-3p, was found in both serum and subcutaneous fat in sildenafil-treated subjects. Treatment with PDE5 inhibitors in humans and murine models of diabetes improves VAT, targeting SIRT1 through a modulation of miR-22-3p expression
NCT01363141^1090^	2016	N/A	Obese individuals with the MetS	• AGE diet • Regular diet	51/49	IR	The L-AGE diet markedly enhanced the protective factors SIRT1
NCT02132091^1091^	2015	N/A	Healthy non-obese participants	• IF + antioxidant (vitamin C 500 mg twice each day and vitamin E 400 IU/day) • IF + placebo capsules	17 (cross-over)	Expression of genes reflecting aging and oxidative stress; dietary satisfaction; diet compliance	A marginal increase (2.7%) was detected in SIRT3 expression due to the IF diet
NCT01765946^1048^	2015	N/A	Prediabetic patients	• Metformin (1500 mg/day) • Placebo (for 2 months)	19/19	Effects of metformin on metabolic parameters, longevity pathway effectors, AMPK activation, chromatin accessibility of the SIRT1 promoter, telomere length, and the N-glycan profile	Metformin increased SIRT1 expression
NCT02011906^1092^	2015	N/A	CAD patients	• OE (4 g/day of n-3 fatty acids and 400 IU of vitamin E) • OP (4 g/day of n-3 fatty acids and vitamin E placebo) • PP (received both n-3 fatty acids and vitamin E placebo soft gels) for 2 months	21/20/19	SIRT1 and PGC-1α gene expression and serum levels of antioxidant enzymes	Gene expression of SIRT1 and PGC-1α increased significantly in the OE group. Supplementation of n-3 fatty acids in combination with vitamin E may have beneficial effects on CAD patients by increasing gene expression of SIRT1 and PGC-1α
NCT02122575^1093^	2015	N/A	Healthy volunteers	• 24-hour fast/ then was fed a fixed-calorie meal	19	NLRP3 inflammasome activation	In a human macrophage line, depletion of the mitochondrial-enriched SIRT deacetylase SIRT3 increased NLRP3 inflammasome activation in association with excessive mitochondrial ROS production. Nutrient levels regulate the NLRP3 inflammasome, in part through SIRT3-mediated mitochondrial homeostatic control
PACTR201407000856135^1049^	2015	N/A	Obese patients with/without T2D	• Obese patients without T2D received fenofibrate 160 mg/day • Obese patients with T2D received fenofibrate 160 mg/day • Obese patients with T2D received fenofibrate (160 mg/day) and pioglitazone (15 mg/day), for eight weeks	15/15/14	Serum SIRT1 and fetuin A	SIRT1 levels in obese patients with T2D were significantly lower than its levels in obese patients. Fenofibrate, alone and in combination with pioglitazone, significantly increased SIRT1 level
ACTRN12613000874718^1094^	2015	N/A	Middle-aged, inactive men	• SSG (3d/week) • CYC (3d/week) • Normal activity and dietary patterns, for 8 weeks	10/11/11	The efficacy to improve risk-factors associated with the prevention of T2D	There were no differences within or between conditions for protein content of SIRT1
NCT01890070^1095^	2014	N/A	Healthy volunteers	• Baseline (fasting) • FRW (fasting + 250 mL red wine) • MM • MMRW (MM + 250 mL red wine) • McD • McD + 250 mL red wine	24 (cross-over)	Oxidized LDL level, oxidative and inflammatory gene expression	SIRT2 expression increased significantly in comparison of FRW versus MMRW. The value of the Pearson coefficient shows a positive correlation between SIRT2 and catalase expression in McD and MMRW as well as a negative correlation between expression of SIRT2 and C-C motif ligand 5 in MM and McD
NCT00691210^1096^	2013	I	Relapsed/refractory biopsy proven lymphoma patients	• A vorinostat fixed dose of 400 mg orally on days 1 to 14 of a 21-day cycle. Niacinamide was given orally on days 1 through 14 of a 21-day cycle and escalated as follows: 20 mg/kg, 40 mg/kg, 60 mg/kg, 80 mg/kg, and 100 mg/kg.	25	Maximal tolerated dose and dose-limiting toxicity of vorinostat and niacinamide in combination; Overall response rate and duration of response	Treatment of diffuse large B-cell lymphoma with the combination of SIRT and deacetylase inhibitors leads to synergistic cytotoxicity and acetylation of Bcl6 and p53

*α-LA* alpha-lipoic acid, *AdipoR* adiponectin receptor, *BDNF* brain-derived neurotrophic factor, *BFR* blood flow restriction, *BMI* body mass index, *BODE* BMI, airway obstruction, dyspnea, exercise tolerance, *CAD* coronary artery disease, *CE* cycle ergometry, *CHO* carbohydrate, *CKD* chronic kidney disease, *CLA* conjugated linoleic acid, *CO* calanus finmarchicus oil, *CON* control, *COPD* chronic obstructive pulmonary disease, *CR* caloric restriction, *CTLA4* cytotoxic T-lymphocyte-associated protein 4, *CYC* continuous stationary cycling, *DHEAS* dehydroepiandrosterone sulfate, *EAA* essential amino acid, *ER* endoplasmic reticulum, *GC* green cardamom, *HbA1c* hemoglobin A1c, *HDL-C* low high density lipoprotein cholesterol, *h-FABP* heart-type fatty acid binding protein, *HFM* high-saturated fat, *HMUFA* high monounsaturated fatty acid, *HSFA* high saturated fatty acid, *ICAM1* intercellular adhesion molecule 1, *IF* intermittent fasting, *IR* insulin resistance, *L-AGE* restricted AGE intake, *LC* load carriage, *LDL* low density lipoprotein, *LDL-C* low-density lipoprotein cholesterol, *LFHCC* low-fat, high-complex carbohydrate, *LOX1* lectin-like oxidized LDL receptor 1, *McD* McDonald’s meal, *MDD* major depressive disorder, *MetS* metabolic syndrome, *MM* Mediterranean meal, *NAFLD* nonalcoholic fatty liver disease, *NO* nitric oxide, *PBMC* peripheral blood mononuclear cell, *PCOS* polycystic ovarian syndrome, *PD* Parkinson’s disease, *PDE5* phosphodiesterase type 5, *PEB* pinitol-enriched beverage, *PJ* pomegranate juice, *PUFA* polyunsaturated fatty acid, *QOL* quality of life, *RA* rheumatoid arthritis, *REE* resting energy expenditure, *RT* resistance training, *RYGB* Roux-en-Y gastric bypass, *SAT* subcutaneous adipose tissue, *SIO* sacha inchi oil, *SIRT* sirtuin, *SSG* small-sided game, *T2D* type 2 diabetes, *TC* total cholesterol, *TG* triglyceride, *UC* ulcerative colitis, *VAT* visceral adipose tissue, *VCAM1* vascular cell adhesion molecule 1, *VD* vitamin D, *YBLI* yoga-based lifestyle intervention, *YMLI* yoga-and meditation-based lifestyle intervention

**Table 2:** Summary of published clinical trials on Sirtuin activators

Trial [ref]	Year	Phase	Participant	Intervention/ Comparison	Sample size of intervention/ comparison	Outcome	Main Findings
NCT02245932^1056^	2020	N/A	COPD	• Resveratrol (150 mg/day) • Placebo (for 4 weeks)	11/10	Mitochondrial function	Muscle mitochondrial biogenesis regulators SIRT1 was not improved by resveratrol. An unexpected decline was shown in lean mass with resveratrol supplementation in patients with COPD
IRCT20181029041490N1^978^	2019	N/A	Patients with T2D and CHD	• Resveratrol (500 mg/day) • Placebo (for 4 weeks)	28/28	IR	Resveratrol upregulated PPAR-γ and SIRT1 in PBMC of T2DM patients with CHD
IRCT201511233664N16^1058^	2018	N/A	NAFLD patients	• The CR diet (prescribed low-calorie diet) • Resveratrol (600 mg/day) • Placebo capsules (600 mg/day starch, for 12 weeks)	30/30/30	Anthropometric indices, metabolic parameters, and serum SIRT1 levels	No significant changes were seen in SIRT1 levels in any group
NCT01668836^1052^	2018	N/A	Healthy subjects	• Resveratrol (500 mg/day) • CR (1000 cal/day, for 30 days)	24/24	Gene expression of SIRT1 and endogenous secretory receptor	Both resveratrol supplementation and CR stimulated SIRT1 serum concentrations
NCT02244879^1054^	2018	N/A	T2D patients	• Resveratrol (500 mg/day) • Resveratrol (40 mg/day) • Placebo (inert microcellulose) for 6 months	43/43/42	Association between changes in SIRT1 level and variation in H3K56ac value	Increased SIRT1 expression was associated with significant H3K56ac content reduction and increased serum antioxidant activity in T2D patients. SIRT1-mediated changes in the epigenome and in the antioxidant, response might impact on diabetes-associated risk factors
NCT01504854^7^	2017	II	Mild-moderate AD patients	• Resveratrol (500 mg orally once daily and a dose escalation by 500-mg increments every 13 weeks, ending with 1000 mg twice daily) • Placebo (matching placebo, for 52 weeks)	19/19	Safety and tolerability as well as effects on AD biomarkers and volumetric MRI	Resveratrol decreases CSF MMP9, modulates neuro-inflammation, and induces adaptive immunity. SIRT1 activation may be a viable target for treatment or prevention of neurodegenerative disorders
NCT01031108^990^	2017	I	T2D patients	• Oral SRT2104 (2.0 g/day) • Placebo (Sirtris Pharmaceuticals 2.0 g/day, for 28 days)	15/14	Pharmacokinetics of SRT2014; Cardiovascular effects of SRT2104; Endogenous fibrinolysis and monocyte and platelet activation; Metabolic effects	Short-term SIRT1 activation in humans is well tolerated and has predominantly neutral effects on markers of endothelial function and platelet-monocyte function
NCT01453491^1061^	2016	N/A	Patients with mild to moderate UC	• SRT2104 (500 mg/day) • SRT2104 (50 mg/day, for 8 weeks)	13/13	Colonic exposure, safety, and clinical activity of SRT2104	SRT2104 did not demonstrate significant clinical activity in mild to moderately active UC
NCT01031108^991^	2016	I	Healthy cigarette smokers and T2D patients	• Oral SRT2104 (2.0 g/day) • Placebo (2.0 g/day, for 28 days)	11/13 (healthy cigarette smokers);7/8 (T2D patients)	Pulse wave analysis and velocity; blood pressure	Compared to placebo, treatment with SRT2104 was associated with a significant reduction in augmentation pressure. SRT2104 may improve arterial compliance in otherwise healthy cigarette smokers and in people with T2D, without affecting resting measures of blood pressure
NCT01668836^1053^	2016	N/A	Healthy participants	• Resveratrol (500 mg/day) • Low-calorie diet (1000 cal/day, for 30 days)	24/24	Serum lipid parameter, glucose, insulin, oxidative stress, C-reactive protein, and SIRT1	CR and resveratrol significantly increased plasma concentrations of SIRT1
NCT01154101^1097^	2015	IIa	Stable plaque-psoriasis	• SRT2104 (250 mg/day) • SRT2104 (500 mg/day) • SRT2104 (1000 mg/day) • Placebo, for 84 consecutive days	9/12/11/7	The change in histological assessments of skin biopsies of psoriatic lesions; the assessment of effect of SRT2104 on Psoriasis Area Severity Index and Physician Global Assessment scores in patients with moderate to severe plaque psoriasis	Substantial improvement was found in 9 subjects following 84 days of treatment with SRT2104. Although absorption was relatively linear with dose, we did not observe a dose-response in the histology endpoint
NCT01014117^1055^	2015	N/A	Healthy, nonsmoking, male volunteers	• SRT2104 (2.0 g/day) • Placebo on days 1–6 and SRT2104 (2.0 g) on day 7 • Placebo, for seven consecutive days	8/8/8	LPS-induced IL-6 and IL-8 release; LPS-induced coagulation; LPS-induced leukocyte transcriptional responses	SRT2104 attenuated LPS-induced release of the cytokines IL-6 and IL-8. SRT2104 also reduced the LPS-induced acute phase protein response (C-reactive protein). SRT2104 inhibited activation of coagulation, as reflected by lower plasma levels of the prothrombin fragment F1 + 2. Activation of the vascular endothelium and the fibrinolytic system was not influenced by SRT2104
EudraCT number 2009-010720-26^1098^	2014	II	T2D patients	• SRT2104 (0.25 g/day) • SRT2104 (0.5 g/day) • SRT2104 (1.0 g/day) • SRT2104 (2.0 g/day) • Placebo (once daily, for 28 days)	45/46/45/45/46	Changes in fasting and post-prandial glucose and insulin	Treatment with SRT2104 for 28 days did not result in improved glucose or insulin control. Treatment with SRT2104 was associated with improvement in lipid profiles
NCT01150955^1059^	2014	N/A	Obese males	• Trans-resveratrol (500 mg three times per day) • Placebo (three times per day, for 5 weeks)	12/12	Effect of body composition and age on GH-stimulated STAT5b phosphorylation and IGF-1, SOCS2, and CISH mRNA in muscle and fat; The impact of resveratrol treatment on GH activity; Impact of inhibiting or knocking down SIRT1 on effects of GH in vitro.	Resveratrol administration had no impact on body composition, serum IGF-1, or GH signaling in vivo, and SIRT1 knock down or inhibition did not affect GH signaling in vitro
NCT01150955^1060^	2013	N/A	Obese but otherwise healthy men	• Trans-resveratrol (500 mg thrice daily) • Placebo (thrice daily, for 4 weeks)	12/12	Insulin sensitivity	Short-term supplementation with high-dose resveratrol is not associated with detectable physiological effects in obese subjects with modest IR
NCT01031108^8^	2013	I	Healthy volunteers	• SRT2104 (2.0 g/day) • Placebo (Sirtris Pharmaceuticals Inc, for 28 days)	24 (cross-over)	Lipid profile and vascular, endothelial, and platelet function	Compared with placebo, serum lipid profile improved during SRT2104 administration, with reductions in serum TC, LDL-C, and TG concentrations. SIRT1 activation may have a beneficial role in patients at risk of developing or with established cardiovascular disease
NCT00823381^1057^	2012	N/A	Non-obese, postmenopausal women	• Resveratrol supplementation (75 mg/day) • CR targeted to achieve a 5% weight loss within 12 weeks • Placebo, for 12 weeks	15/15/14	Metabolic function	Resveratrol did not affect its putative molecular targets, including AMPK and SIRT1, in either skeletal muscle or adipose tissue

*AD* Alzheimer’s disease, *AMPK* adenosine monophosphate-activated protein kinase, *CHD* coronary heart disease, *CISH* cytokine-inducible SH, *COPD* chronic obstructive pulmonary disease, *CR* caloric restriction, *CSF* cerebrospinal fluid, *GH* growth hormone, *H3K56ac* histone 3 acetylation at the 56 lysine residue, *IL* interleukin, *IR* insulin resistance, *LDL-C* low-density lipoprotein cholesterol, *LPS* lipopolysaccharide, *NAFLD* nonalcoholic fatty liver disease, *PBMC* peripheral blood mononuclear cell, *SIRT* sirtuin, *SOCS2* suppressor of cytokine signaling 2, *STAT5b* signal transducer and activator of transcription 5b, *T2D* type 2 diabetes, *TC* total cholesterol, *TG* triglyceride, *UC* ulcerative colitis

RCTs have been conducted on all continents, with Asia having the largest number of studies (*n* = 23), followed by Europe (*n* = 20) and North America (*n* = 12). Iran (*n* = 18), Italy (*n* = 5), and the United States (*n* = 12) ranked first in Asia, Europe, and North America, respectively. However, only one RCT was conducted in Oceania (Australia) and Africa (Egypt), respectively. These studies recruited from 2005 to 2019 and were published between 2012 and 2022. The peak years of study recruitment ranged from 2015 to 2016, whereas the majority of the studies were published between 2015 and 2020. Most studies examined SIRT protein expression in human samples, where blood samples (*n* = 40, 74.1%) were mostly used. Fat (*n* = 6, 11.1%) and muscle (*n* = 8, 14.8%) tissues were also used in several studies. Regardless of the tissue type, SIRT1 was the protein most focused on among the SIRT protein family. Some researchers evaluated SIRT3 protein expression in blood samples and adipose tissue.

Sixty-three RCTs included participants with more than 10 different diseases and conditions. Among them, the largest number of studies included participants with metabolic diseases (*n* = 30, 42.9%), including type 2 diabetes, obesity, and metabolic syndrome, followed by studies that recruited healthy participants (*n* = 17, 24.3%) such as healthy elderly participants, healthy employees, and healthy volunteers. A limited number of studies recruited participants with other diseases. For example, only three studies investigated a gynecological disease (polycystic ovarian syndrome), skin disease (systemic lupus erythematosus), and cancer (lymphoma), respectively.

With regard to the intervention/comparison in the study, about half of the studies (*n* = 34, 47.9%) focused on dietary interventions such as vitamin D and caloric restriction, with a few studies focusing on exercise interventions (*n* = 11, 15.5%) as well as drug and surgical treatments (*n* = 6, 8.5%). For example, supplementation of crocin or crocetin effectively improved gene expression of SIRT1 in coronary artery disease patients compared with the placebo.^1040,1041^ However, curcumin, administered to 67 overweight or obese patients with polycystic ovarian syndrome, led to a non-significant increase in SIRT1 expression, after 12 weeks compared to placebo.^1042^ With regard to exercise intervention, three RCTs in India highlighted that a yoga-based lifestyle intervention led to a significant increase in SIRT1.^1043–1045^ On the contrary, among 70 rheumatoid arthritis patients, the mRNA expression levels of SIRT1 were not found to be statistically different in the yoga vs. non-yoga group.^1046^ As for medical treatment, three RCTs demonstrated that treatment with sildenafil, metformin, fenofibrate alone or in combination with pioglitazone up-regulated SIRT1 gene expression.^1047–1049^ In addition, after Roux-en-Y gastric bypass, the expression of SIRT1 and SIRT3 increased compared to the baseline in 13 obese, non-diabetic patients.^1050^

Only 23.9% (*n* = 17) of published RCTs explored the effects of SIRT activators on physiological function. As a well-known SIRT activator,^1051^ resveratrol (*n* = 10, 14.1%) received more attention compared with SRT2104 (*n* = 7, 9.9%) in these studies. Several RCTs showed that resveratrol supplementation could effectively increase the expression or concentration of SIRT1.^978,1052–1054^ Moreover, resveratrol performed important physiological functions by activating SIRT1, such as beneficial effects on neuro-inflammation and adaptive immunity.^7^ Similarly, SRT2014 played an important role by activating SIRT1, such as reduction in endotoxin-induced cytokine release and coagulation activation.^1055^ However, some intervention studies reported that resveratrol did not affect its putative molecular target.^1056–1058^ For example, a double-blind randomized placebo-controlled proof-of-concept study conducted in the Netherlands suggested that the muscle mitochondrial biogenesis regulator SIRT1 was not improved by resveratrol.^1056^ A few studies suggested that significant clinical activities were not observed after supplementation with resveratrol and SRT2014.^1059–1061^

As shown in Table 2, three RCTs (4.2%) focused on the impact of nicotinamide, a known SIRT inhibitor,^976^ on physiological function in different patients.^1062–1064^ However, all three studies suggested that nicotinamide might not act through its putative molecular target. For example, findings from a long-term human clinical trial reported that NR supplementation did not affect SIRT activity in human skeletal muscle.^1064^ In addition, a clinical study evaluating the pharmacodynamics efficacy of nicotinamide as an inhibitor of SIRT revealed that over 12 months of nicotinamide treatment, no sustained inhibitions of SIRT activity were detected.^1063^ This might be attributed to the small sample size and short intervention duration in these three RCTs. Thus, further studies are needed to explore nicotinamide as a clinical therapeutic method by inhibiting SIRT activity.

Although several published RCTs have shown inconsistent findings, most studies have suggested that dietary, exercise, and drug interventions can enhance SIRT signaling, and SIRT activators played an important role in physiological functions by activating SIRTs. Given the important impact of the SIRT protein family on health and disease, the relatively small number of trials, study limitations and single study sites, further larger sample, longer intervention period, and multicenter RCTs are needed.

## Conclusion

Since the discovery of the SIRT family members, the understanding of this protein family has become increasingly comprehensive and profound. The studies summarized herein provide strong evidence that SIRTs play important roles in the body. Considering that the roles of SIRTs vary in different types of biological processes and human diseases, it will be of great significance to focus attention on the mechanisms regarding the seven SIRTs under different conditions and the specific function of each SIRT. Recent advances in technology (e.g., development of omics, gene KO and knockin) may facilitate elucidation of the specific molecular mechanisms of SIRTs, providing new perspectives for the pathogenesis of human diseases and targets for treatments. Clinical trials to verify the biomarkers and therapeutic potential of SIRTs are still lacking and are warranted in the future. Thus, this review has systematically highlighted the recent advances with respect to the role of SIRTs, which may aid the design of future research, and thereby reveal the diagnostic and therapeutic potential of SIRTs.

### Future directions

1. To further clarify the biological regulation mechanism of SIRTs in different kinds of diseases and health conditions, and the interaction relationship between different kinds of SIRTs.
2. To validate SIRTs as potential diagnostic and prognostic biomarkers for specific diseases at a large population level.
3. To develop and validate more specific agonists and inhibitors of different kinds of SIRTs, and to explore and confirm their efficacy and safety in disease prevention and treatment in basic and clinical studies.
4. SIRTs, such as SIRT7, which has been studied finitely, should be the focus of future research.
5. Studies incorporating a multidisciplinary perspective provide a more comprehensive understanding of the roles of SIRTs.

## Supplementary information

Supplementary Tables
