# Supplementary material for: The sirtuin family in health and disease
Source: Signal Transduct Target Ther. 2022 Dec 29;7:402. doi: 10.1038/s41392-022-01257-8 (PMC9797940; doi:10.1038/s41392-022-01257-8)
Supplement: Supplementary file 1 — Supplementary Tables [file 41392_2022_1257_MOESM1_ESM.docx]

**Supplementary Materials for**

**The sirtuin family in health and disease**

Qi-Jun Wu ^1, 2, 3, 4#^, Tie-Ning Zhang ^5#^, Huan-Huan Chen ^6^, Xue-Fei Yu ^2, 5^, Jia-Le Lv ^1, 2, 4^, Yu-Yang Liu ^1, 2, 4^, Ya-Shu Liu ^1, 2, 4^, Gang Zheng ^1, 2, 4^, Jun-Qi Zhao ^1, 2, 4^, Yi-Fan Wei ^1, 2, 4^, Jing-Yi Guo ^1, 2, 4^, Fang-Hua Liu ^1, 2, 4^, Qing Chang ^1, 2, 4^, Yi-Xiao Zhang ^7^, Cai-Gang Liu ^8*^, Yu-Hong Zhao ^1, 2, 4*^

1. Key Laboratory of Precision Medical Research on Major Chronic Disease, Shengjing Hospital of China Medical University, Shenyang, China.

2. Department of Clinical Epidemiology, Shengjing Hospital of China Medical University, Shenyang, China.

3. Department of Obstetrics and Gynecology, Shengjing Hospital of China Medical University, Shenyang, China.

4. Clinical Research Center, Shengjing Hospital of China Medical University, Shenyang, China.

5. Department of Pediatrics, Shengjing Hospital of China Medical University, Shenyang, China.

6. Department of Oncology, Shengjing Hospital of China Medical University, Shenyang, China.

7. Department of Urology, Shengjing Hospital of China Medical University, Shenyang, China.

8. Department of Cancer, Breast Cancer Center, Shengjing Hospital of China Medical University, Shenyang, China.

# These two authors contributed equally to this work.

Correspondence to: Cai-Gang Liu ([angel-s205@163.com](mailto:angel-s205@163.com)); Yu-Hong Zhao ([zhaoyuhong@sj-hospital.org](mailto:zhaoyuhong@sj-hospital.org)).

**This PDF file includes:**

**Tables S1 to S2**

**Table S1. Most relevant sirtuin activators**

| **Compound/drug [ref]** | **Modulated targets and effects on SIRT activity** | **Biological effects** |
| --- | --- | --- |
| **Natural polyphenols** | | |
| Resveratrol | SIRT1 EC_1.5_ = 46.2 μM ^1^  SIRT2 EC_1.5_ > 300 μM ^1^  SIRT3 EC_1.5_ > 300 μM ^1^ | 1. Reduced fasting glucose, insulin, and insulin resistance, increased high-density lipoprotein-cholesterol levels and total antioxidant capacity, and upregulated PPAR-γ and SIRT1 in the peripheral blood mononuclear cells of type 2 diabetes mellitus patients with coronary heart disease;^2^ 2. Reduced Aβ-dependent NF-κB activation in microglia and neuronal loss;^3^ 3. Increased the angiogenic response of endothelial cells.^4^ |
| Fisetin |  | 1. Provided neuroprotection against aging induced oxidative stress, apoptotic cell death, neuroinflammation, and neurodegeneration in rat brain;^5^ 2. Facilitated SIRT1-mediated deacetylation of PPARγ and Forkhead box protein O1, and enhanced the association of SIRT1 with the PPARγ promoter, leading to suppression of PPARγ transcriptional activity, thereby repressing adipogenesis;^6^ 3. Inhibited high glucose-induced ROS production in monocytes through the activation of SIRTs and forkhead box O3a.^7^ |
| Quercetin |  | 1. Potentiated insulin secretion, β-cells protected against oxidative damages;^8^ 2. Induced apoptotic and autophagic responses;^9^ 3. Inhibit cancerous cell proliferation and invasion through suppression of parathyroid hormone receptor 1 by reducing its expression, decreased cell viability and its adhesion and migration, and attenuated MMP-2 and MMP-9;^10^ 4. Reduced cell viability, enhanced apoptosis in invasive ovarian cancer cell lines, and enhanced mitochondrial-induced apoptotic pathways.^11^ |
| Piceatannol |  | 1. Restored cell viability and cell apoptosis under H_2_O_2_ injury;^12^ 2. Possessed the protective effects against bleomycin-induced pulmonary fibrosis due to the direct pulmonary protective effects which enhanced the effect of autophagy in vitro and in vivo and finally led to the decreased number of activated myofibroblasts;^13^ 3. Arrested colon cancer cells in the S-phase, and downregulated cyclin D1, B1 and cyclin-dependent kinases 4.^14^ |
| Isoliquiritigenin |  | 1. Repressed the proliferation, migration and invasion of non-small cell lung cancer cells in vitro;^15^ 2. Reduced oxidative damage and alleviated mitochondrial impairment by SIRT1 activation in experimental diabetic neuropathy;^16^ 3. Suppressed hyperglycemia-induced renal fibrosis and apoptosis, improved hyperglycemia-induced renal inflammation and oxidation, reduced HG-induced renal inflammation, oxidative stress, fibrosis, and apoptosis in NRK-52E cells, abrogated HG-induced inflammatory and oxidative injuries in NRK-52E cells via SIRT1;^17^ 4. Alleviated fatty acid-induced cellular lipid accumulation in vitro, improved glucose tolerance and energy metabolism in vivo, and reduced intracellular lipid content dependent on SIRT1.^18^ |
| Butein |  | 1. Attenuated sepsis-induced brain injury through alleviation of cerebral inflammation, oxidative stress and apoptosis by SIRT1 signaling activation;^19^ 2. Inhibited acetylation of P53 and protect nucleus pulposus cells against hyperglycaemia-induced apoptosis and senescence through SIRT1 activation.^20^ |
| **Synthetic SIRT activators** | | |
| SRT1460 | SIRT1 EC_1.5_ = 2.9 μM^1^  SIRT2 EC_1.5_ > 300 μM^1^  SIRT3 EC_1.5_ > 300 μM^1^ | 1. Inhibited cell viability and cell growth;^21^ 2. Reduced the infarct area of the heart, ameliorated cardiac dysfunction, weakened oxidative stress induced by myocardial ischemia/reperfusion injury.^22^ |
| SRT1720 | SIRT1 EC_1.5_ = 0.16 μM^1^  SIRT2 EC_1.5_ = 37 μM^1^  SIRT3 EC_1.5_ > 300 μM^1^ | 1. Downregulated the level of collagen I and α-SMA, increased PPARα and CPT1a level, and decreased lipid deposition;^23^ 2. Protected from diet-induced diabesity and promoted energy expenditure in metabolic tissues;^24^ 3. Reduced cell viability in vitro and ex vivo, increased the number of apoptotic cells, induced mitochondrial outer membrane permeabilization with the generation of mitochondrial reactive oxygen species and autophagy, and decreased the growth of human adult T-cell leukemia/lymphoma tumor xenografts in immunodeficient mice;^25^ 4. Inhibited the growth of bladder cancer in organoids and murine models through the SIRT1-HIF axis.^26^ |
| SRT2104 | SIRT1 EC_1.5_ = 0.43 μM^27^ | 1. Upregulated key ovulatory and angiogenic genes: prostaglandin endoperoxide synthase 2, epiregulin, fibroblast growth factor 2 and vascular endothelial growth factor A, and decreased viable cell numbers;^28^ 2. Extended survival of male mice on a standard diet and preserves bone and muscle mass;^29^ 3. Attenuated lipopolysaccharide-induced release of the cytokines interleukin-6 and interleukin-8, reduced the lipopolysaccharide-induced acute phase protein response (C-reactive protein), and inhibited activation of coagulation, as reflected by lower plasma levels of the prothrombin fragment F1+2.^30^ |
| SRT2183 | SIRT1 EC_1.5_ = 0.36 μM^1^  SIRT2 EC_1.5_ > 300 μM^1^  SIRT3 EC_1.5_ > 300 μM^1^ | 1. Suppressed glioma cell growth and destroyed neurospheres in vitro and induced glioma cell cycle arrest and apoptosis, accompanying by upregulation of the pro-apoptotic Bim and downregulation of Bcl-2 and Bcl-xL;^31^ 2. Inhibited the growth of ovarian cancer cells, increased the accumulation of BAX, cleaved-caspase 3 and cleaved-PARP, and decreased the level of anti-apoptotic Bcl-2 and Mcl-1;^32^ 3. Reduced TUNEL-positive apoptosis after H_2_O_2_ treatment, increased cyclooxygenase-2 expression in a dose-dependent manner.^33^ |
| SRT3025 | SIRT1 EC_1.5_ < 1 μM^21, 34^ | 1. Inhibited tumor growth in vivo, decreased viability mainly of SU86.86 cells in culture;^21^ 2. Attenuated proprotein convertase subtilisin/kexin type 9 secretion and its binding to LDL receptor, decreased plasma levels of LDL-cholesterol and total cholesterol, and reduced atherosclerosis.^35^ |
| UBCS039 | SIRT6 EC_50_ = 38 μM^36^ | 1. Induced deacetylation of SIRT6-targeted histone H3 sites, led to autophagosome accumulation, and induced autophagic flux in human cancer cells;^37^ 2. Triggered reactive oxygen species accumulation, activated the AMPK/ULK1 signaling pathway, and induced autophagy-associated cell death;^37^ 3. Alleviated lung injury in the mouse acute respiratory distress syndrome model and enhanced autophagy and M2 polarization in isolated alveolar macrophages.^38^ |
| MDL-800 | SIRT6 EC_50_ = 10.3 μM^39^ | 1. Inhibited the proliferation of human hepatocellular carcinoma cells via SIRT6-driven cell-cycle arrest and was effective in a tumor xenograft model;^39^ 2. Induced remarkable cell cycle arrest at the G0/G1 phase in non-small cell lung carcinoma HCC827 and PC9 cells, enhanced the antiproliferation of epidermal growth factor receptor tyrosine kinase inhibitors in osimertinib-resistant HCC827 and PC9 cells as well as in patient-derived primary tumor cells, and suppressed mitogen-activated protein kinase pathway;^40^ 3. Decreased the TGF-β1-induced activation of myofibroblast and extracellular matrix production by regulating SIRT6-dependent β-catenin acetylation and the TGF-β1/Smad signaling pathway;^41^ 4. Mitigated unilateral ureteral obstruction-induced renal tubulointerstitial inflammation and fibrosis.^41^ |

AMPK, AMP-activated protein kinase; HG, high glucose; LDL, low-density lipoprotein; MMP, matrix metalloproteinases; NF-κB, kappa-light-chain-enhancer of activated B cells; PPARγ, peroxisome proliferator-activated receptor gamma; ROS, reactive oxygen species; SIRT, sirtuin; TGF, transforming growth factor.

**Table S2.** **Most relevant sirtuin inhibitors**

| **Compound/drug [ref]** | **Modulated targets and effects on SIRT activity** | **Biological effects** |
| --- | --- | --- |
| **β-napthol-containing inhibitors** | | |
| Salermide | SIRT1 IC_50_ = 43 μM^42^  SIRT2 IC_50_ = 25 μM^42^ | 1. Induced apoptosis in MOLT4, KG1A, K562, SW480, Raji and nonsmall-cell lung cancer cells;^43, 44^ 2. Induced the reactivation of proapoptotic genes that were aberrantly repressed in cancer cells by SIRT1-mediated lysine 16 of histone H4 deacetylation;^44^ 3. Potent antiproliferative on human leukemia MOLT4 cell lines, human breast MDA-MB-231, and colon RKO cancer cell lines and potent against colorectal carcinoma cancer stem cells;^45^ 4. Protected against oculopharyngeal muscular dystrophy.^42^ |
| Splitomicin | SIRT1 IC_50_ = 96 μM^42^  SIRT2 IC_50_ = 113 μM^42^ | 1. Reversed both IPC-mediated lysine deacetylation and IPC-induced cardioprotection;^46^ 2. Enhanced TF mRNA expression in stimulated endothelial cells, reduced deacetylase activity, enhanced NF-κB/p65 nuclear translocation, increased TF activity in mouse carotid artery in vivo.^47^ |
| Cambinol | SIRT1 IC_50_ = 56 μM^48^  SIRT2 IC_50_ = 59 μM^48^  SIRT5: 42% inhibition at 300 μM^48^ | 1. Reduced the expression of N-Myc protein and up-regulated the expression of the other SIRT1 target genes including early growth response 1, Kv channel interacting protein 4 and phospholipase C beta 1;^49^ 2. Altered the morphology of 2 human hepatocellular carcinoma cell lines, reduced the expression of poorly differentiated markers α-fetoprotein and glypican, and impaired cell migration in a dose-dependent manner.^50^ |
| Sirtinol | SIRT1 IC_50_ = 131 μM^51^  SIRT2 IC_50_ = 38-58 μM^51, 52^ | 1. Inhibited viability of breast, lung, prostate and oral cancer cells;^53-56^ 2. Induced senescence-like growth arrest in human breast cancer MCF-7 cells and lung cancer H1299 cells;^53^ 3. Induced apoptotic and autophagic cell death in MCF-7 human breast cancer cells;^57^ 4. Induced significant growth inhibition or apoptosis in cells from adult T-cell leukemia-lymphoma patients and leukemic cell lines, especially human T-cell leukemia virus-related cell lines.^58^ |
| HR-73 | SIRT1 IC_50_ < 5 μM^59^ | 1. Decreased human immunodeficiency virus transcription through Tat acetylation.^59^ |
| **Indole derivatives** | | |
| EX-527 | SIRT1 IC_50_ = 0.098 μM^60^  SIRT2 IC_50_ = 19.6 μM^60^  SIRT3 IC_50_ = 48.7 μM^60^ | 1. Increased the cytotoxic effect of gemcitabine in vitro in PANC-1 cells;^61^ 2. Decreased the tumour growth of xenografted mice with endometrial and lung cancer cells;^62, 63^ 3. Decreased the viability of control HHUA cells and the survival of HEC151 cells and reduced cisplatin resistance in HEC1B cells with mutated and non-functional p53;^63^ 4. Protected against oculopharyngeal muscular dystrophy.^42^ |
| AC-93253 | SIRT1 IC_50_ = 45.3 μM^64^  SIRT2 IC_50_ = 6 μM^64^  SIRT3 IC_50_ = 24.6 μM^64^ | 1. Influenced the expression of the ABC melanoma gene cluster, blocked the cell cycle at G1 phase, and sensitized the MDA-MB-435S melanoma cell line to doxorubicin;^65^ 2. Increased the acetylation levels of α-tubulin in a dose-dependent manner and the amount of histone H4 acetylation at lysine 16, induced hyperacetylation of p53 in NCI-H460 cells, and selectively induce cytotoxicity in cancer cells.^64^ |
| Inauhzin | SIRT1 IC_50_ = 0.7- 2 μM^66^ (22331558) | 1. Inhibited cell proliferation, induced senescence and apoptosis of human cancer cell without genotoxicity, and repressed the growth of xenograft tumors derived from human lung cancer H460 and colon cancer HCT116 cells harbouring p53;^66^ 2. Decreased mitochondrial membrane potential, increased mitochondrial calcium ^2+^, raised the intracellular ROS level, and suppressed the growth in HCT116 and DLD-1 cells;^67^ 3. Induced ribosomal stress and the RPL11/RPL5-MDM2 interaction, activating p53, and suppressed cancer cell growth by dually targeting SIRT1 and inosine monophosphate dehydrogenase 2.^68^ |
| Ro31-8220 | SIRT1 IC_50_ = 3.5 μM^69^  SIRT2 IC_50_ = 0.8 μM^69^ | 1. Reduced the PKC activity and the tau phosphorylation pattern in a human neuroblastoma cell line;^70^ 2. Suppressed cell proliferation in androgen-dependent prostate cancer LNCaP cells;^71^ 3. Inhibited PKC and activated the c-Jun N terminal kinase, decreased the proportion of the cell growth-promoting Bcl-xL splice variant.^72^ |
| **SIRT-rearranging ligands** | | |
| SirReal2 | SIRT1 IC_50_ > 100μM^73^  SIRT2 IC_50_ = 0.14 μM^73^  SIRT3 IC_50_ > 100μM^73^ | 1. Led to tubulin hyperacetylation in HeLa cells and induced destabilization of the checkpoint protein BubR1;^73^ 2. Increased the levels of phosphorylated Cx43 on S368 and the levels of acetylated MEK1/2, decreased the membrane localization of Cx43 between cumulus cells, and increased the Cx43 acetylation levels of cumulus-oocyte complexes;^74^ 3. Increased the rates of oocyte activation, cytoplasmic fragmentation, and spindle defects;^75^ 4. Led to elevated intracellular reactive oxygen species accumulation, low ATP production, and reduced mitochondrial membrane potential in oocytes.^76^ |
| **Tenovins** | | |
| Tenovin-1 | Not determined due to lack of water solubility | 1. Improved the cytopathic effect-induced DENV2 and inhibited the release of progeny viruses, decreased the viral protein level and mRNA level, and inhibited the inflammatory response caused by DENV2, reducing the release of inflammatory factors during viral infection;^77^ 2. Reduced tumor growth in the BL2 and ARN8 mouse xenograft model.^78^ |
| Tenovin-6 | SIRT1 IC_50_ = 21 μM^78^  SIRT2 IC_50_ = 10 μM^78^  SIRT3 IC_50_ = 67 μM^78^ | 1. Delayed the growth of xenograft tumors derived from ARN8 cells;^78^ 2. Suppressed anchorage-independent growth of gastric cancer cells, induced different levels of apoptosis and phases of cell-cycle arrest depending on the cell lines with some manifesting gap 1 and others showing synthesis phase cell-cycle arrest, and induced autophagy or p53 activation in gastric cancer cells depending on the status of *TP53* gene;^79^ 3. Increased microtubule-associated protein 1 light chain 3 level in diverse cell types in a time- and dose-dependent manner;^80^ 4. Killed Ewing sarcoma cells in vitro and prohibited tumor growth and spread in an established xenograft model in zebrafish;^81^ 5. Detered the disease progression of chronic myelogenous leukemia in mice model.^82^ |
| **Nicotinamide and its analogues** | | |
| Nicotinamide | SIRT1 IC_50_ = 120 μM^82^  SIRT2 IC_50_ = 100 μM^83^  SIRT3 IC_50_ = 50 μM^82^  SIRT5 IC_50_ = 150 μM^82^  SIRT6 IC_50_ = 184 μM^84^ | 1. Blocked proliferation and induced apoptosis of chronic lymphocytic leukemia cells;^85^ 2. Inhibited the growth and viability of human prostate cancer cells through inhibiting SIRT1;^54^ 3. Led to cancer cell death in triple-negative breast cancer via mitochondrial dysfunction and activation of ROS by bifurcating metabolic pathways (reverse electron transport and lipid metabolism);^86^ 4. Impaired naive T cell effector transition but also effector T cells themselves, reduced mechanistic target of rapamycin complex 1 activity independently of nicotinamide adenine dinucleotide metabolism, decreasing IFNγ translation and regulating T cell transcriptional factors critical to effector/memory fate;^87^ 5. Delayed tumor growth in vivo and improved survival of melanoma-bearing mice.^88^ |
| AK-7 | SIRT2 IC_50_ = 15.5 μM^89^ | 1. Brain-permeability but limited metabolic stability;^89^ 2. Increased traumatic brain injury-induced microglial activation both in vivo and in vitro, accompanied by a large increase in the expression and release of inflammatory cytokines;^90^ 3. Limited the ability of adoptively transferred antigen-specific CD4+ T cells to cause autoimmune encephalomyelitis in mice and limited disease in lupus-prone MRL/lpr mice;^91^ 4. Improved the behavior and neuropathological phenotype and extended survival of R6/2 HD mice, ameliorated HD neuropathology in R6/2 mice, and reduced the polyglutamine aggregation in R6/2 brain.^92^ |
| **Other SIRT inhibitors** | | |
| Suramin | SIRT1 IC_50_ = 297 nM^93^  SIRT2 IC_50_ = 1150 nM^93^  SIRT5 IC_50_ = 22 μM^94^ | 1. Decreased the survival of navitoclax-treated SW480 cells, enhanced the amount of tumor suppressor miR-1-3p, and inhibited miR-1-3p binding to Staphylococcal Nuclease and Tudor Domain Containing 1 specifically;^95^ 2. Inhibited severe acute respiratory syndrome coronavirus 2 infection in cell culture by interfering with early steps of the replication cycle;^96^ 3. Inhibited Chikungunya virus replication by interacting with virions and blocking the early steps of infection;^97^ 4. Perturbed mitochondrial membrane potential and ATP levels, and triggered pyruvate accumulation and proline catabolism;^98^ 5. Attenuated intervertebral disc degeneration by inhibiting NF-κB signalling pathway.^99^ |
| Aristoforin | SIRT1 IC_50_ = 7 μM^100^  SIRT2 IC_50_ = 21 μM^100^ | 1. Antiproliferative activity on human umbilical vein endothelial cells;^100^ 2. Decreased multidrug resistance-associated protein 2 in photodynamic therapy (T0- and T6+) and affected intracellular accumulation of HY in HT-29 colon adenocarcinoma cells;^101^ 3. Induced significant accumulation of HY in HT-29 cells and HCT-116 cells;^102^ 4. Inhibited the proliferation of lymphatic endothelial cells derived from lung and dermis, induced cell cycle arrest of endothelial cells at lower concentrations, and induced apoptosis at higher concentrations.^103^ |
| AGK2 | SIRT1 IC_50_ > 50 μM^42^  SIRT2 IC_50_ = 3.5 μM^42^  SIRT3 IC_50_ > 50 μM^104^ | 1. Potent against glioblastoma multiforme cancer stem cells;^45^ 2. Blocked the therapeutic action of NR against nonalcoholic fatty liver disease pathologies and NR-induced Fndc5 deubiquitination/deacetylation;^105^ 3. Alleviated allergic asthma symptoms such as lung histological changes (immune cell and mast cell infiltration, collagen deposition, and α-smooth muscle actin expression) and serum Ig levels (IgE, ovalbumin-specific IgE, IgG1, and IgG2a), reduced the levels of pro-inflammatory cytokines (TNF-α, IL-1β, IL-4, IL-5, and IL-6) and inflammatory mediators (myeloperoxidase, eosinophil peroxidase, and TNF-α) in the bronchoalveolar lavage fluid and lung tissues;^106^ 4. Protective against Parkinson’s disease;^104, 107^ 5. Rescued dopaminergic neurons from α-synuclein toxicity in various Parkinson’s disease models.^104^ |
| Tripos 360702 | SIRT2 IC_50_ = 51 μM^108^ |  |
| MC2141 | SIRT1 IC_50_ = 9.8 μM^109^  SIRT2 IC_50_ = 12.3 μM^109^ | 1. Reduced the accumulation of PCNA in the cells, reduced the percentage of caspase-positive cells, and reduced the expression of p53 in the cells.^110^ |
| MC2494 | SIRT1 IC_50_ = 38.5 μM^111^  SIRT2 IC_50_ = 58.6 μM^111^ | 1. Displayed context-specific regulation in cancer cell cycle progression, regulated cancer cell cycle progression at molecular level, and inhibited cell migration in both MCF7 and MDA-MB-231 cells;^112^ 2. Displayed tumor-selective potential in vitro, in leukemic blasts ex vivo, and in vivo in both xenograft and allograft cancer models and displayed tumor-preventive activity by blocking 7,12-dimethylbenz(α)anthracene-induced mammary gland hyperproliferation in vivo;^111^ 3. Blocked mitochondrial biogenesis and function in terms of ATP synthesis and energy metabolism.^113^ |

fMLP, N-formyl-L-methionyl-L-leucyl-Lphenylalanine; HD, Huntington's disease; HY, hypericin; Ig, immunoglobulins; IL, interleukin; IPC, ischaemic preconditioning; NF-κB, kappa-light-chain-enhancer of activated B cells; NR, nicotinamide riboside; PKC, protein kinase C; ROS, reactive oxygen species; TF, tissue factor; TNF, tumor necrosis factor.

1. Milne, J.C. et al. Small molecule activators of SIRT1 as therapeutics for the treatment of type 2 diabetes. *Nature* **450**, 712-716 (2007).

2. Hoseini, A. et al. The effects of resveratrol on metabolic status in patients with type 2 diabetes mellitus and coronary heart disease. *Food Funct* **10**, 6042-6051 (2019).

3. Gan, L. & Mucke, L. Paths of convergence: sirtuins in aging and neurodegeneration. *Neuron* **58**, 10-14 (2008).

4. Potente, M. et al. SIRT1 controls endothelial angiogenic functions during vascular growth. *Genes Dev* **21**, 2644-2658 (2007).

5. Singh, S., Singh, A.K., Garg, G. & Rizvi, S.I. Fisetin as a caloric restriction mimetic protects rat brain against aging induced oxidative stress, apoptosis and neurodegeneration. *Life Sci* **193**, 171-179 (2018).

6. Kim, S.C. et al. Fisetin induces Sirt1 expression while inhibiting early adipogenesis in 3T3-L1 cells. *Biochem Biophys Res Commun* **467**, 638-644 (2015).

7. Kim, A., Lee, W. & Yun, J.M. Luteolin and fisetin suppress oxidative stress by modulating sirtuins and forkhead box O3a expression under in vitro diabetic conditions. *Nutr Res Pract* **11**, 430-434 (2017).

8. Youl, E. et al. Quercetin potentiates insulin secretion and protects INS-1 pancreatic beta-cells against oxidative damage via the ERK1/2 pathway. *Br J Pharmacol* **161**, 799-814 (2010).

9. He, Y. et al. Quercetin induces autophagy via FOXO1-dependent pathways and autophagy suppression enhances quercetin-induced apoptosis in PASMCs in hypoxia. *Free Radic Biol Med* **103**, 165-176 (2017).

10. Li, S. et al. Quercetin suppresses the proliferation and metastasis of metastatic osteosarcoma cells by inhibiting parathyroid hormone receptor 1. *Biomed Pharmacother* **114**, 108839 (2019).

11. Teekaraman, D., Elayapillai, S.P., Viswanathan, M.P. & Jagadeesan, A. Quercetin inhibits human metastatic ovarian cancer cell growth and modulates components of the intrinsic apoptotic pathway in PA-1cell line. *Chem Biol Interact* **300**, 91-100 (2019).

12. Liu, Y., Li, J., Ye, Z., Ma, T. & Li, Z. Protective Effects of Piceatannol against Selenite-Induced Cataract and Oxidative Damage in Rats. *Curr Eye Res* **47**, 1272-1278 (2022).

13. Sheng, H. et al. Antifibrotic Mechanism of Piceatannol in Bleomycin-Induced Pulmonary Fibrosis in Mice. *Front Pharmacol* **13**, 771031 (2022).

14. Wolter, F., Clausnitzer, A., Akoglu, B. & Stein, J. Piceatannol, a natural analog of resveratrol, inhibits progression through the S phase of the cell cycle in colorectal cancer cell lines. *J Nutr* **132**, 298-302 (2002).

15. Cui, Y. et al. Isoliquiritigenin inhibits non-small cell lung cancer progression via m(6)A/IGF2BP3-dependent TWIST1 mRNA stabilization. *Phytomedicine* **104**, 154299 (2022).

16. Yerra, V.G., Kalvala, A.K. & Kumar, A. Isoliquiritigenin reduces oxidative damage and alleviates mitochondrial impairment by SIRT1 activation in experimental diabetic neuropathy. *J Nutr Biochem* **47**, 41-52 (2017).

17. Huang, X. et al. Isoliquiritigenin prevents hyperglycemia-induced renal injuries by inhibiting inflammation and oxidative stress via SIRT1-dependent mechanism. *Cell Death Dis* **11**, 1040 (2020).

18. Zhang, L. et al. Administration of isoliquiritigenin prevents nonalcoholic fatty liver disease through a novel IQGAP2-CREB-SIRT1 axis. *Phytother Res* **35**, 3898-3915 (2021).

19. Zhu, Y. et al. SIRT1 activation by butein attenuates sepsis-induced brain injury in mice subjected to cecal ligation and puncture via alleviating inflammatory and oxidative stress. *Toxicol Appl Pharmacol* **363**, 34-46 (2019).

20. Zhang, Z. et al. The Sirt1/P53 Axis in Diabetic Intervertebral Disc Degeneration Pathogenesis and Therapeutics. *Oxid Med Cell Longev* **2019**, 7959573 (2019).

21. Chini, C.C. et al. SIRT1-Activating Compounds (STAC) Negatively Regulate Pancreatic Cancer Cell Growth and Viability Through a SIRT1 Lysosomal-Dependent Pathway. *Clin Cancer Res* **22**, 2496-2507 (2016).

22. Zhao, S. & Yu, L. Sirtuin 1 activated by SRT1460 protects against myocardial ischemia/reperfusion injury. *Clin Hemorheol Microcirc* **78**, 271-281 (2021).

23. Zhang, Y. et al. SIRT1 prevents cigarette smoking-induced lung fibroblasts activation by regulating mitochondrial oxidative stress and lipid metabolism. *J Transl Med* **20**, 222 (2022).

24. Feige, J.N. et al. Specific SIRT1 activation mimics low energy levels and protects against diet-induced metabolic disorders by enhancing fat oxidation. *Cell Metab* **8**, 347-358 (2008).

25. Kozako, T. et al. SRT1720 induces SIRT1-independent cell death in adult T-cell leukemia/lymphoma. *FEBS J* **289**, 3477-3488 (2022).

26. Tan, P. et al. SRT1720 inhibits the growth of bladder cancer in organoids and murine models through the SIRT1-HIF axis. *Oncogene* **40**, 6081-6092 (2021).

27. Yee Ng P. et al. The identification of the sirt1 activator srt2104 as a clinical candidate. *Lett. Drug design discov.* **10(9)**, 793-797 (2013).

28. Sapuleni, J., Szymanska, M. & Meidan, R. Diverse actions of sirtuin-1 on ovulatory genes and cell death pathways in human granulosa cells. *Reprod Biol Endocrinol* **20**, 104 (2022).

29. Mercken, E.M. et al. SRT2104 extends survival of male mice on a standard diet and preserves bone and muscle mass. *Aging Cell* **13**, 787-796 (2014).

30. van der Meer, A.J. et al. The Selective Sirtuin 1 Activator SRT2104 Reduces Endotoxin-Induced Cytokine Release and Coagulation Activation in Humans. *Crit Care Med* **43**, e199-202 (2015).

31. Ye, T. et al. Sirtuin1 activator SRT2183 suppresses glioma cell growth involving activation of endoplasmic reticulum stress pathway. *BMC Cancer* **19**, 706 (2019).

32. Sun, T. et al. SRT2183 impairs ovarian cancer by facilitating autophagy. *Aging (Albany NY)* **12**, 24208-24218 (2020).

33. He, W. et al. Sirt1 activation protects the mouse renal medulla from oxidative injury. *J Clin Invest* **120**, 1056-1068 (2010).

34. Sirtris Pharmaceuticals Inc.: **US8343997B2** (2013).

35. Miranda, M.X. et al. The Sirt1 activator SRT3025 provides atheroprotection in Apoe-/- mice by reducing hepatic Pcsk9 secretion and enhancing Ldlr expression. *Eur Heart J* **36**, 51-59 (2015).

36. You, W. et al. Structural Basis of Sirtuin 6 Activation by Synthetic Small Molecules. *Angew Chem Int Ed Engl* **56**, 1007-1011 (2017).

37. Iachettini, S. et al. Pharmacological activation of SIRT6 triggers lethal autophagy in human cancer cells. *Cell Death Dis* **9**, 996 (2018).

38. Wang, Q.L. et al. Sirtuin 6 regulates macrophage polarization to alleviate sepsis-induced acute respiratory distress syndrome via dual mechanisms dependent on and independent of autophagy. *Cytotherapy* **24**, 149-160 (2022).

39. Huang, Z. et al. Identification of a cellularly active SIRT6 allosteric activator. *Nat Chem Biol* **14**, 1118-1126 (2018).

40. Shang, J.L., Ning, S.B., Chen, Y.Y., Chen, T.X. & Zhang, J. MDL-800, an allosteric activator of SIRT6, suppresses proliferation and enhances EGFR-TKIs therapy in non-small cell lung cancer. *Acta Pharmacol Sin* **42**, 120-131 (2021).

41. Jin, J. et al. Loss of Proximal Tubular Sirtuin 6 Aggravates Unilateral Ureteral Obstruction-Induced Tubulointerstitial Inflammation and Fibrosis by Regulation of beta-Catenin Acetylation. *Cells* **11** (2022).

42. Pasco, M.Y. et al. Characterization of sirtuin inhibitors in nematodes expressing a muscular dystrophy protein reveals muscle cell and behavioral protection by specific sirtinol analogues. *J Med Chem* **53**, 1407-1411 (2010).

43. Liu, P.Y. et al. The histone deacetylase SIRT2 stabilizes Myc oncoproteins. *Cell Death Differ* **20**, 503-514 (2013).

44. Lara, E. et al. Salermide, a Sirtuin inhibitor with a strong cancer-specific proapoptotic effect. *Oncogene* **28**, 781-791 (2009).

45. Rotili, D. et al. Discovery of salermide-related sirtuin inhibitors: binding mode studies and antiproliferative effects in cancer cells including cancer stem cells. *J Med Chem* **55**, 10937-10947 (2012).

46. Nadtochiy, S.M., Redman, E., Rahman, I. & Brookes, P.S. Lysine deacetylation in ischaemic preconditioning: the role of SIRT1. *Cardiovasc Res* **89**, 643-649 (2011).

47. Breitenstein, A. et al. Sirt1 inhibition promotes in vivo arterial thrombosis and tissue factor expression in stimulated cells. *Cardiovasc Res* **89**, 464-472 (2011).

48. Heltweg, B. et al. Antitumor activity of a small-molecule inhibitor of human silent information regulator 2 enzymes. *Cancer Res* **66**, 4368-4377 (2006).

49. Marshall, G.M. et al. SIRT1 promotes N-Myc oncogenesis through a positive feedback loop involving the effects of MKP3 and ERK on N-Myc protein stability. *PLoS Genet* **7**, e1002135 (2011).

50. Portmann, S. et al. Antitumor effect of SIRT1 inhibition in human HCC tumor models in vitro and in vivo. *Mol Cancer Ther* **12**, 499-508 (2013).

51. Mai, A. et al. Design, synthesis, and biological evaluation of sirtinol analogues as class III histone/protein deacetylase (Sirtuin) inhibitors. *J Med Chem* **48**, 7789-7795 (2005).

52. Grozinger, C.M., Chao, E.D., Blackwell, H.E., Moazed, D. & Schreiber, S.L. Identification of a class of small molecule inhibitors of the sirtuin family of NAD-dependent deacetylases by phenotypic screening. *J Biol Chem* **276**, 38837-38843 (2001).

53. Ota, H. et al. Sirt1 inhibitor, Sirtinol, induces senescence-like growth arrest with attenuated Ras-MAPK signaling in human cancer cells. *Oncogene* **25**, 176-185 (2006).

54. Jung-Hynes, B., Nihal, M., Zhong, W. & Ahmad, N. Role of sirtuin histone deacetylase SIRT1 in prostate cancer. A target for prostate cancer management via its inhibition? *J Biol Chem* **284**, 3823-3832 (2009).

55. Kojima, K. et al. A role for SIRT1 in cell growth and chemoresistance in prostate cancer PC3 and DU145 cells. *Biochem Biophys Res Commun* **373**, 423-428 (2008).

56. Alhazzazi, T.Y. et al. Sirtuin-3 (SIRT3), a novel potential therapeutic target for oral cancer. *Cancer* **117**, 1670-1678 (2011).

57. Wang, J. et al. Sirtinol, a class III HDAC inhibitor, induces apoptotic and autophagic cell death in MCF-7 human breast cancer cells. *Int J Oncol* **41**, 1101-1109 (2012).

58. Kozako, T. et al. High expression of the longevity gene product SIRT1 and apoptosis induction by sirtinol in adult T-cell leukemia cells. *Int J Cancer* **131**, 2044-2055 (2012).

59. Pagans, S. et al. SIRT1 regulates HIV transcription via Tat deacetylation. *PLoS Biol* **3**, e41 (2005).

60. Broussy, S., Laaroussi, H. & Vidal, M. Biochemical mechanism and biological effects of the inhibition of silent information regulator 1 (SIRT1) by EX-527 (SEN0014196 or selisistat). *J Enzyme Inhib Med Chem* **35**, 1124-1136 (2020).

61. Zhang, J.G. et al. Sirtuin 1 facilitates chemoresistance of pancreatic cancer cells by regulating adaptive response to chemotherapy-induced stress. *Cancer Sci* **105**, 445-454 (2014).

62. Chen, G. et al. Suppression of Sirt1 sensitizes lung cancer cells to WEE1 inhibitor MK-1775-induced DNA damage and apoptosis. *Oncogene* **36**, 6863-6872 (2017).

63. Asaka, R. et al. Sirtuin 1 promotes the growth and cisplatin resistance of endometrial carcinoma cells: a novel therapeutic target. *Lab Invest* **95**, 1363-1373 (2015).

64. Zhang, Y. et al. Identification of a small molecule SIRT2 inhibitor with selective tumor cytotoxicity. *Biochem Biophys Res Commun* **386**, 729-733 (2009).

65. Karwaciak, I. et al. AC-93253 triggers the downregulation of melanoma progression markers and the inhibition of melanoma cell proliferation. *Chem Biol Interact* **236**, 9-18 (2015).

66. Zhang, Q. et al. A small molecule Inauhzin inhibits SIRT1 activity and suppresses tumour growth through activation of p53. *EMBO Mol Med* **4**, 298-312 (2012).

67. Sun, Y. et al. Inhibition of nuclear deacetylase Sirtuin-1 induces mitochondrial acetylation and calcium overload leading to cell death. *Redox Biol* **53**, 102334 (2022).

68. Zhang, Q. et al. The role of IMP dehydrogenase 2 in Inauhzin-induced ribosomal stress. *Elife* **3** (2014).

69. Trapp, J. et al. Adenosine mimetics as inhibitors of NAD+-dependent histone deacetylases, from kinase to sirtuin inhibition. *J Med Chem* **49**, 7307-7316 (2006).

70. Shim, K.H. et al. Small-molecule drug screening identifies drug Ro 31-8220 that reduces toxic phosphorylated tau in Drosophila melanogaster. *Neurobiol Dis* **130**, 104519 (2019).

71. Shiota, M. et al. Inhibition of protein kinase C/Twist1 signaling augments anticancer effects of androgen deprivation and enzalutamide in prostate cancer. *Clin Cancer Res* **20**, 951-961 (2014).

72. Hai, Y. et al. A G-tract element in apoptotic agents-induced alternative splicing. *Nucleic Acids Res* **36**, 3320-3331 (2008).

73. Rumpf, T. et al. Selective Sirt2 inhibition by ligand-induced rearrangement of the active site. *Nat Commun* **6**, 6263 (2015).

74. Xu, D., He, H., Liu, D., Geng, G. & Li, Q. A novel role of SIRT2 in regulating gap junction communications via connexin-43 in bovine cumulus-oocyte complexes. *J Cell Physiol* **235**, 7332-7343 (2020).

75. Xu, D. et al. SIRT2 functions in aging, autophagy, and apoptosis in post-maturation bovine oocytes. *Life Sci* **232**, 116639 (2019).

76. Xu, D. et al. SIRT2 Inhibition Results in Meiotic Arrest, Mitochondrial Dysfunction, and Disturbance of Redox Homeostasis during Bovine Oocyte Maturation. *Int J Mol Sci* **20** (2019).

77. Wan, Y. et al. Tenovin-1 inhibited dengue virus replication through SIRT2. *Eur J Pharmacol* **907**, 174264 (2021).

78. Lain, S. et al. Discovery, in vivo activity, and mechanism of action of a small-molecule p53 activator. *Cancer Cell* **13**, 454-463 (2008).

79. Ke, X., Qin, Q., Deng, T., Liao, Y. & Gao, S.J. Heterogeneous Responses of Gastric Cancer Cell Lines to Tenovin-6 and Synergistic Effect with Chloroquine. *Cancers (Basel)* **12** (2020).

80. Yuan, H., Tan, B. & Gao, S.J. Tenovin-6 impairs autophagy by inhibiting autophagic flux. *Cell Death Dis* **8**, e2608 (2017).

81. Ban, J. et al. Suppression of deacetylase SIRT1 mediates tumor-suppressive NOTCH response and offers a novel treatment option in metastatic Ewing sarcoma. *Cancer Res* **74**, 6578-6588 (2014).

82. Yuan, H. et al. Activation of stress response gene SIRT1 by BCR-ABL promotes leukemogenesis. *Blood* **119**, 1904-1914 (2012).

83. Tervo, A.J. et al. An in silico approach to discovering novel inhibitors of human sirtuin type 2. *J Med Chem* **47**, 6292-6298 (2004).

84. Hu, J., He, B., Bhargava, S. & Lin, H. A fluorogenic assay for screening Sirt6 modulators. *Org Biomol Chem* **11**, 5213-5216 (2013).

85. Audrito, V. et al. Nicotinamide blocks proliferation and induces apoptosis of chronic lymphocytic leukemia cells through activation of the p53/miR-34a/SIRT1 tumor suppressor network. *Cancer Res* **71**, 4473-4483 (2011).

86. Jung, M. et al. Nicotinamide (niacin) supplement increases lipid metabolism and ROS-induced energy disruption in triple-negative breast cancer: potential for drug repositioning as an anti-tumor agent. *Mol Oncol* **16**, 1795-1815 (2022).

87. Agliano, F., Karginov, T.A., Menoret, A., Provatas, A. & Vella, A.T. Nicotinamide breaks effector CD8 T cell responses by targeting mTOR signaling. *iScience* **25**, 103932 (2022).

88. Scatozza, F. et al. Nicotinamide inhibits melanoma in vitro and in vivo. *J Exp Clin Cancer Res* **39**, 211 (2020).

89. Taylor, D.M. et al. A brain-permeable small molecule reduces neuronal cholesterol by inhibiting activity of sirtuin 2 deacetylase. *ACS Chem Biol* **6**, 540-546 (2011).

90. Yuan, F. et al. SIRT2 inhibition exacerbates neuroinflammation and blood-brain barrier disruption in experimental traumatic brain injury by enhancing NF-kappaB p65 acetylation and activation. *J Neurochem* **136**, 581-593 (2016).

91. Hisada, R. et al. The deacetylase SIRT2 contributes to autoimmune disease pathogenesis by modulating IL-17A and IL-2 transcription. *Cell Mol Immunol* **19**, 738-750 (2022).

92. Chopra, V. et al. The sirtuin 2 inhibitor AK-7 is neuroprotective in Huntington's disease mouse models. *Cell Rep* **2**, 1492-1497 (2012).

93. Trapp, J. et al. Structure-activity studies on suramin analogues as inhibitors of NAD+-dependent histone deacetylases (sirtuins). *ChemMedChem* **2**, 1419-1431 (2007).

94. Schuetz, A. et al. Structural basis of inhibition of the human NAD+-dependent deacetylase SIRT5 by suramin. *Structure* **15**, 377-389 (2007).

95. Lehmusvaara, S., Haikarainen, T., Saarikettu, J., Martinez Nieto, G. & Silvennoinen, O. Inhibition of RNA Binding in SND1 Increases the Levels of miR-1-3p and Sensitizes Cancer Cells to Navitoclax. *Cancers (Basel)* **14** (2022).

96. Salgado-Benvindo, C. et al. Suramin Inhibits SARS-CoV-2 Infection in Cell Culture by Interfering with Early Steps of the Replication Cycle. *Antimicrob Agents Chemother* **64** (2020).

97. Albulescu, I.C. et al. Suramin Inhibits Chikungunya Virus Replication by Interacting with Virions and Blocking the Early Steps of Infection. *Viruses* **12** (2020).

98. Zoltner, M. et al. Suramin exposure alters cellular metabolism and mitochondrial energy production in African trypanosomes. *J Biol Chem* **295**, 8331-8347 (2020).

99. Liu, Z.M. et al. Suramin attenuates intervertebral disc degeneration by inhibiting NF-kappaB signalling pathway. *Bone Joint Res* **10**, 498-513 (2021).

100. Gey, C. et al. Phloroglucinol derivatives guttiferone G, aristoforin, and hyperforin: inhibitors of human sirtuins SIRT1 and SIRT2. *Angew Chem Int Ed Engl* **46**, 5219-5222 (2007).

101. Semelakova, M., Jendzelovsky, R. & Fedorocko, P. Drug membrane transporters and CYP3A4 are affected by hypericin, hyperforin or aristoforin in colon adenocarcinoma cells. *Biomed Pharmacother* **81**, 38-47 (2016).

102. Semelakova, M., Mikes, J., Jendzelovsky, R. & Fedorocko, P. The pro-apoptotic and anti-invasive effects of hypericin-mediated photodynamic therapy are enhanced by hyperforin or aristoforin in HT-29 colon adenocarcinoma cells. *J Photochem Photobiol B* **117**, 115-125 (2012).

103. Rothley, M. et al. Hyperforin and aristoforin inhibit lymphatic endothelial cell proliferation in vitro and suppress tumor-induced lymphangiogenesis in vivo. *Int J Cancer* **125**, 34-42 (2009).

104. Outeiro, T.F. et al. Sirtuin 2 inhibitors rescue alpha-synuclein-mediated toxicity in models of Parkinson's disease. *Science* **317**, 516-519 (2007).

105. Li, D.J. et al. NAD(+)-boosting therapy alleviates nonalcoholic fatty liver disease via stimulating a novel exerkine Fndc5/irisin. *Theranostics* **11**, 4381-4402 (2021).

106. Kim, Y.Y. et al. AGK2 ameliorates mast cell-mediated allergic airway inflammation and fibrosis by inhibiting FcepsilonRI/TGF-beta signaling pathway. *Pharmacol Res* **159**, 105027 (2020).

107. Garske, A.L., Smith, B.C. & Denu, J.M. Linking SIRT2 to Parkinson's disease. *ACS Chem Biol* **2**, 529-532 (2007).

108. Tervo, A.J. et al. Discovering inhibitors of human sirtuin type 2: novel structural scaffolds. *J Med Chem* **49**, 7239-7241 (2006).

109. Rotili, D. et al. Benzodeazaoxaflavins as sirtuin inhibitors with antiproliferative properties in cancer stem cells. *J Med Chem* **55**, 8193-8197 (2012).

110. Sirotkin, A.V. et al. Comparison of the effects of synthetic and plant-derived mTOR regulators on healthy human ovarian cells. *Eur J Pharmacol* **854**, 70-78 (2019).

111. Carafa, V. et al. RIP1-HAT1-SIRT Complex Identification and Targeting in Treatment and Prevention of Cancer. *Clin Cancer Res* **24**, 2886-2900 (2018).

112. Carafa, V. et al. Enzymatic and Biological Characterization of Novel Sirtuin Modulators against Cancer. *Int J Mol Sci* **20** (2019).

113. Carafa, V. et al. The Pan-Sirtuin Inhibitor MC2494 Regulates Mitochondrial Function in a Leukemia Cell Line. *Front Oncol* **10**, 820 (2020).
